# Comparing sociocultural features of cholera in three endemic African settings

**DOI:** 10.1186/1741-7015-11-206

**Published:** 2013-09-18

**Authors:** Christian Schaetti, Neisha Sundaram, Sonja Merten, Said M Ali, Erick O Nyambedha, Bruno Lapika, Claire-Lise Chaignat, Raymond Hutubessy, Mitchell G Weiss

**Affiliations:** 1 Department of Epidemiology and Public Health, Swiss Tropical and Public Health Institute, Socinstrasse 57, PO Box, 4002, Basel, Switzerland; 2 University of Basel, Petersplatz 1, 4003 Basel, Switzerland; 3 Public Health Laboratory Ivo de Carneri, PO Box 122, Wawi, Chake-Chake, Pemba, Zanzibar, United Republic of Tanzania; 4 Department of Sociology and Anthropology, Maseno University, Private Bag, Maseno, Kenya; 5 Department of Anthropology, University of Kinshasa, PO Box 127, Kinshasa XI, Democratic Republic of Congo; 6 Global Task Force on Cholera Control, World Health Organization, 20 Avenue Appia, 1211 Geneva 27, Switzerland; 7 Initiative for Vaccine Research, World Health Organization, 20 Avenue Appia, 1211 Geneva 27, Switzerland

**Keywords:** Endemic cholera, Sociocultural features, Community study, Eastern Africa

## Abstract

**Background:**

Cholera mainly affects developing countries where safe water supply and sanitation infrastructure are often rudimentary. Sub-Saharan Africa is a cholera hotspot. Effective cholera control requires not only a professional assessment, but also consideration of community-based priorities. The present work compares local sociocultural features of endemic cholera in urban and rural sites from three field studies in southeastern Democratic Republic of Congo (SE-DRC), western Kenya and Zanzibar.

**Methods:**

A vignette-based semistructured interview was used in 2008 in Zanzibar to study sociocultural features of cholera-related illness among 356 men and women from urban and rural communities. Similar cross-sectional surveys were performed in western Kenya (n = 379) and in SE-DRC (n = 360) in 2010. Systematic comparison across all settings considered the following domains: illness identification; perceived seriousness, potential fatality and past household episodes; illness-related experience; meaning; knowledge of prevention; help-seeking behavior; and perceived vulnerability.

**Results:**

Cholera is well known in all three settings and is understood to have a significant impact on people’s lives. Its social impact was mainly characterized by financial concerns. Problems with unsafe water, sanitation and dirty environments were the most common perceived causes across settings; nonetheless, non-biomedical explanations were widespread in rural areas of SE-DRC and Zanzibar. Safe food and water and vaccines were prioritized for prevention in SE-DRC. Safe water was prioritized in western Kenya along with sanitation and health education. The latter two were also prioritized in Zanzibar. Use of oral rehydration solutions and rehydration was a top priority everywhere; healthcare facilities were universally reported as a primary source of help. Respondents in SE-DRC and Zanzibar reported cholera as affecting almost everybody without differentiating much for gender, age and class. In contrast, in western Kenya, gender differentiation was pronounced, and children and the poor were regarded as most vulnerable to cholera.

**Conclusions:**

This comprehensive review identified common and distinctive features of local understandings of cholera. Classical treatment (that is, rehydration) was highlighted as a priority for control in the three African study settings and is likely to be identified in the region beyond. Findings indicate the value of insight from community studies to guide local program planning for cholera control and elimination.

## Background

Cholera is an ancient enteric disease that originated from the Ganges delta [1]. It is caused by the bacterium *Vibrio cholerae* that exists in the aquatic environment independent from human hosts [2,3]. *V. cholerae* produces an enterotoxin, which is the direct cause of acute watery diarrhea in humans. Cholera is characterized by loss of large volumes of rice-water-like stool leading to severe dehydration and concurrent electrolyte depletion [4]. Case fatality rates without treatment may reach 50% [5]. Timely administration of oral rehydration solutions or infusions is the principal treatment [6].

### Global cholera burden and sub-Saharan Africa as a hotspot

Cholera case estimates officially reported to the World Health Organization (WHO) ranged between 190,000 and 320,000 for the years 2008, 2009 and 2010, and between 5,000 and 7,500 deaths were reported [7-9]. These figures, however, are highly under-reported because of limitations in surveillance, including case definitions, and also fear of trade-related and travel-related sanctions; they likely represent less than 10% of the true burden [10]. A recent study estimated the number of people at risk of endemic cholera globally at 1.4 billion, with an annual burden of endemic cholera of 2.8 million cases and 91,000 deaths [11]. Cholera thrives mostly in low-income and middle-income countries in Africa, Asia and the Caribbean [12].

According to the latest estimates, 39% of the population in sub-Saharan Africa lived without safe water in 2010 (vs 51% in 1990), with an urban share of 17% and a rural share of 51% [13]. Use of improved sanitation in the same region has been increasing from 26% to 30% since 1990. Similar to the estimates on water supply, there is also an urban/rural divide: 43% of urban people benefited from improved sanitation in 2010 versus only 23% in rural areas.

The public health burden of cholera is still intolerable in sub-Saharan Africa despite the above noted progress in the provision of safe water and sanitation. Consequently, and because of the recent huge outbreaks in Zimbabwe, Pakistan and Haiti, the 64th World Health Assembly adopted a new resolution in 2011 to strengthen the global fight against cholera [14].

The WHO recommends provision of sufficient, safe water and adequate sanitation and hygiene (WASH) as the mainstay to prevent cholera [15]. Official recommendations also include the use of oral cholera vaccines (OCVs) as a supplementary public health tool for preemptive or reactive control of cholera outbreaks [16].

### Professional versus community-reported burden of cholera

The burden of cholera may be characterized with reference to professional indicators, and it may also be studied with reference to the local vantage point of community experience. The public health importance of cholera with reference to professional indicators has been extensively studied (that is, disease-related morbidity and mortality, characterization and distribution of pathogens, classical epidemiologic risk factors, economic costs and so on) [2-4,11,17-19]. It is widely recognized that cholera can spread rapidly and easily within countries (for example, Kenya [20]) and across continents. Official WHO policy recommends the development of ‘national and subregional action plans that include cross-border collaboration […] to enhance multidisciplinary prevention, and preparedness and response activities’ for effective cholera control [15].

In contrast with public health professionals, communities may prioritize other issues. Lay people may care more about illness-related costs than morbidity, and they may perceive the risk of illness with reference to their local rather than regional experiences. Community perceptions of the causes of illness may also differ from professional concepts, and this may affect their perceived relevance and value of recommended strategies for control. Neglecting or underestimating local sociocultural aspects of cholera and priorities for control may limit the effectiveness of interventions and control programs [21-23]. This point has been elaborated in a review of social science research on neglected tropical diseases of poverty, which highlights the ‘importance of community participation for the successful introduction, acceptability, and adherence of innovative vector control interventions and new drugs and diagnostics’ [24].

Notwithstanding the acknowledged importance of community-based studies, there are very few in Africa. Some have considered questions about perceived vulnerability and social and environmental aspects of cholera [23,25], but systematic assessment of cholera-related experience, meaning and behavior is lacking. In response to this dearth of community-based research, three sociocultural field studies were undertaken in a WHO initiative to examine local urban and rural features of cholera and community willingness to accept an OCV in eastern Africa. A project in Zanzibar (Tanzania) examined sociocultural features of cholera with a semistructured interview and estimated anticipated acceptance and uptake of OCVs in endemic areas in 2008/2009 [26]. Two additional surveys using an almost identical instrument were conducted in 2010 in endemic settings in western Kenya and southeastern Democratic Republic of Congo (SE-DRC). These three databases on community views of cholera have been analyzed with a focus on site-specific similarities and differences [27,28] (Merten S, Manianga C, Weiss MG, Lapika B, unpublished data). A second set of analyses has examined sociocultural determinants of anticipated OCV acceptance in all three settings [29-31], and of OCV uptake in Zanzibar [32,33].

The aim of the present work is to review and systematically compare local cholera-related recognition, risk perceptions, experience, and meaning in endemic African settings. Knowledge of prevention, help-seeking behavior and perceived vulnerabilities are also considered. Data come from the three cross-sectional interview-based surveys mentioned above. Particular attention is given to site (that is, urban vs rural) and gender-specific features. Given the vastness of eastern Africa, it is expected that some features may be common while others may be locally distinctive, and that a systematic assessment of their distribution in cholera-endemic communities is likely to be relevant for informing a regionally tailored cholera control strategy.

## Methods

### Study settings by country

Study sites for the survey in Zanzibar were chosen in 2008 based on a review of the local cholera burden and deliberations between the Ministry of Health and mass vaccination campaign implementers. Study sites in western Kenya and SE-DRC were selected in 2010 based on (i) epidemiological data collected from recent cholera outbreaks, (ii) comparability of urban and rural sites with reference to the survey sites in Zanzibar and (iii) considerations regarding the security of the research team (SE-DRC) and accessibility (western Kenya). The following is a brief description of the urban and rural study sites in each setting, including the national and local cholera situation and related control activities around the time of the surveys. More details can be found in the individual publications mentioned previously.

#### *SE-DRC: Katanga province, southeastern Democratic Republic of Congo*

The survey took place in Kasenga district, in DRC’s southeastern Katanga province. The first waves of the current seventh cholera pandemic reached DRC in 1974 (then called Zaïre). DRC has reported outbreaks of cholera every year since 1990. The eastern part of the country, which borders the African great lakes region, has traditionally been a focus of cholera, and Katanga province is among the four most affected provinces [34,35]. In 2010, 13,884 cases were reported by the WHO for DRC [9]. In 2011, the United Nations Office for the Coordination of Humanitarian Affairs (OCHA) reported 22,233 cases, with 2,701 cases (12%) coming from Katanga province [36]. In the absence of a proper water and sanitation infrastructure in the area, cholera transmission dynamics and severity of outbreaks are mainly driven by environmental (for example, seasonal rainfall, the El Niño phenomenon) and human factors, such as travel and trade [37].

Primary healthcare in DRC is in principle free, but user fees in the form of a low flat rate or full cost recovery have also been introduced in certain areas [38]. Even if no formal user fees are expected, patients will rarely be treated anywhere in the country if they do not pay informal fees; this and the fact that health centers are often out of stock and understaffed reflects the poor state of the public healthcare system in DRC.

Cholera control activities have been mainly reactive with a focus on patient treatment in the epidemic-prone lakes region. Public awareness and information campaigns have been conducted after outbreaks in bigger cities in southern Katanga region (that is, Lubumbashi, Mbuji-Mayi, and so on) [34]. Outbreak response usually includes setting up of treatment centers organized by governmental and non-governmental institutions.

The small town of Kasenga, located 208 km east of the provincial capital Lubumbashi, was chosen as the urban site. Kasenga is situated on the Luapula River that shares a border with Zambia. Kasenga is divided into eight districts (known as ‘quarters’) with a total population of 27,000 inhabitants on a surface area of 10 km^2^. The town is predominantly populated by Bemba-speaking people. Kasenga is the terminus for land and water transportation systems in the area, including buses from Lubumbashi and boats from Pweto, located north of Kasenga on Lake Mweru. Residents of Kasenga depend mainly on three means of livelihood: agriculture, fishing, and commerce. The urban site in SE-DRC was in a quarter of Kasenga called Mwalimu, which was inhabited by approximately 10,300 inhabitants and is characterized by a high density of buildings. No sanitation is available and a water supply that works only sporadically has been installed only in the last few years.

The island of Nkolé, situated approximately 120 km downstream from Kasenga in Lake Mweru, was chosen as the rural site. Approximately 7,000 mostly Bemba people live in Nkolé; they are mainly engaged in agriculture and fisheries. One part of the population is seasonally migrating to cultivate their fields along the river between November and March when fishing is usually prohibited.

Availability of sound data on cholera morbidity is a problem in the area. A manual review of case registers over the last 3 years at the health posts serving both study sites confirmed the seasonal influence as there were more cholera episodes during the rainy season. Kasenga was itself identified as ‘sanctuary’ for cholera outbreaks among six other cities in eastern DRC [39]. The true cholera burden, however, is likely to be higher because of under-reporting due to the limited accessibility of health services during the rainy season and due to rumors accompanying cholera outbreaks in the past.

#### *Western Kenya: Nyanza province*

Both survey sites in Kenya were in Nyanza province, which borders Lake Victoria. The Lake Victoria region has been regularly affected by the current cholera pandemic since 1977/1978. Cholera outbreaks have disproportionately affected Nyanza province in recent years in comparison to the rest of Kenya [40]. More than 14,000 cholera-related hospital admissions were reported in the province in 1997/1998, with a case fatality rate (CFR) of 4% [41]. Another large cholera outbreak started in late 2007 in the aftermath of post-election violence, causing high mortality rates [42]. In 2009, more than 11,000 countrywide cases were reported to the WHO [8].

Apart from public healthcare facilities, there are private healthcare facilities owned by different stakeholders, including multinationals such as the Aga Khan Foundation, faith-based organizations and individuals. They are thought to offer better services but are very expensive (minimum of KES500 per visit). After the large outbreak of 2009, control activities in the affected areas entailed health talks given to the communities. The health talks were intended to promote awareness of cholera, its mode of transmission, prevention and clinical presentations.

Cholera treatment centers (CTCs) are organized only during an outbreak and all the services are supposed to be free of charge. Private healthcare facilities too are not permitted to charge for treating cholera, according to government policy.

Urban interviews were performed in the provincial capital Kisumu, which is the third largest city in Kenya. In 2004, about 60% of Kisumu’s population lived in slums [43]. Migration into Kisumu from surrounding districts has been predominantly motivated by better resources and employment opportunities. Migrants are most likely to settle in the already impoverished slum areas.

The urban site was Nyalenda A, a slum in West Kolwa location of Kisumu district, characterized by a high population density (23,731 residents living on a 2.8 km^2^ surface area in 1999), poor planning, insufficient infrastructure and a severe shortage of basic facilities such as sanitation, safe water, sewerage and electricity [44]. Only one dispensary and one private clinic served this community at the time of the study. Disposal of solid waste is a major problem. The rural site in Siaya district comprised nine villages in the Kakum Kombewa sublocation. According to a 2007 survey, 3,729 people lived in 1,013 households in the study area [45]. No healthcare facility was available to villagers in the rural site in early 2010. Since then, however, three dispensaries have been constructed [46]. Access to the Siaya district hospital is difficult because of irregular motorized transport. A survey conducted in 2007 revealed that almost every homestead of Kakum Kombewa was dependent on water from unprotected sources and latrine coverage was 74% [45].

#### *Zanzibar archipelago, Tanzania*

The survey in Zanzibar, which is an archipelago 50 km off the coast of mainland Tanzania, was performed on its two major islands, Unguja and Pemba. Approximately 1.2 million people live in Zanzibar, which is a major tourist destination. It is believed that cholera reached Zanzibar as early as 1821 during the first pandemic [1], with subsequent outbreaks in later pandemics. In the current pandemic, cholera was reported for the first time in 1978 [47]. More than 13 outbreaks followed since then and the annual incidence rate reached 0.5 per 1,000 population between 1997 and 2007 [48]. Outbreaks usually follow a seasonal pattern (that is, they occur during flooding in the rainy season), and cholera incidence on the archipelago was shown to be positively influenced by rainfall and temperature [49]. Estimates from the Ministry of Health reported a total of 48 cases with a cholera diagnosis admitted to healthcare facilities in 2008, 736 cases in 2009 and 248 cases in 2010 [50-52].

The public healthcare system in Zanzibar is divided into three levels: primary, secondary and tertiary. Each of the two islands constitutes a zone, headed by a zonal medical officer. Over 100 primary healthcare units serve the population; these units are open during the day to outpatients, provide basic services and are within easy reach for over 90% of the population. Four primary healthcare centers (two per zone) operate on a 24-h basis. These centers can admit up to 30 patients.

Cholera is a recognized priority disease in Zanzibar and control activities follow national guidelines [53]. Once an outbreak has been declared, a concerted response follows that usually involves deployment of cholera treatment kits, personnel for outbreak investigation, clinical treatment and follow-up activities. CTCs are set up in government health facilities or schools close to communities where the outbreak has occurred. Medical treatment (that is, infusions, antibiotics, oral rehydration solution (ORS)) for suspected cholera patients is free; affected families mainly incur direct costs to feed the patient [19].

Chumbuni, in Urban district on Unguja Island, was selected as the urban study site for the survey; Mwambe, a village in Mkoani district on Pemba Island, was the rural study site. Chumbuni was inhabited by approximately 11,000 people at the time of the study [27]. This periurban slum-like extension of the capital of Zanzibar is characterized by a high population density of 15,300 people/km^2^ and brick houses with corrugated roofs. Mwambe was less densely populated (800 people/km^2^), with a population of approximately 8,000 people living in mud houses with thatched roofs in widely scattered hamlets.

### Survey design and instrument

The survey in Zanzibar was conducted from June to August 2008 in collaboration with the Ministry of Health and the Public Health Laboratory, Ivo de Carneri; the survey was followed by a mass vaccination campaign in early 2009. The survey in western Kenya was performed from March to May 2010 in collaboration with Maseno University, Kisumu. The survey in SE-DRC was conducted from August to September 2010 along with researchers from the Universities of Kinshasa and Lubumbashi.

A semistructured explanatory model interview based on the Explanatory Model Interview Catalogue (EMIC) framework [54] was used in all three settings. Each version was developed and adapted to the local context (see Additional files 1, 2, 3).

A random sample of adults was interviewed in each survey. The interview was introduced with a vignette telling the respondents a story about a local person with the key signs and symptoms of cholera. The vignette used in Zanzibar is available as an additional file in Schaetti *et al*. [27]. It was the basis for adaptation and use of vignettes in western Kenya and SE-DRC. Variables elicited from responses to questions about the condition depicted in the vignette were related to several domains: identification; perceived seriousness, fatality and past personal and household episodes of cholera; illness-related experience (operationalized as patterns of distress indicating priority symptoms and concerns about the illness); meaning (perceived causes); priorities for prevention (for example, hygiene and sanitation); behavior (help seeking at home and outside the household); and perceived vulnerability to the illness. Sociodemographic characteristics were also recorded. Interviews were conducted in Kiswahili (in all three settings), and additionally in CiBemba (SE-DRC) and Dholuo (western Kenya).

### Ethics statement

Ethics approval to conduct interviews was obtained by each individual study from the following review bodies: WHO Research Ethics Review Committee (western Kenya, Zanzibar), the University of Kinshasa (SE-DRC), the Kenya Medical Research Institute (western Kenya) and the Ministry of Health Ethics Committee of Zanzibar (Zanzibar). All participants had provided written informed consent prior to being interviewed.

### Approach to analysis

Interview items were analyzed according to both frequency and prominence. Calculation of the prominence was based on whether a category was mentioned spontaneously by the respondent in response to an open-ended question (assigned value of 2), only probing specific categories (assigned value of 1), and accounting for whether it was identified as paramount among all reported categories (additional value of 3). Each domain is presented in a table by setting, divided into panels for overall results, urban and rural site comparisons and gender comparisons. The three most prominent categories for each setting and any category with a significant difference between site and between genders are presented for the domains of illness experience, meaning, knowledge of prevention and behavior. Cross-setting comparisons of the three most prominent categories and site and gender comparisons were performed using the non-parametric Wilcoxon or Kruskal-Wallis test for ranked prominence data and the χ^2^ or Fisher’s exact test for proportions. More detailed and comprehensive data stratified by site for western Kenya and Zanzibar have been published elsewhere [27,28].

## Results

### Sample characteristics

Approximately equal numbers of men and women from urban and rural sites were interviewed in SE-DRC (n = 360), western Kenya (n = 379) and Zanzibar (n = 356). Detailed sample characteristics for all three settings have been presented elsewhere [27,29,31]. In summary, the community samples in all settings were mostly engaged in agriculture (>25%). This occupation was more frequent in the rural sites except in SE-DRC, where the rural site was a fishing village. Fishing was the major activity of 16.4% of people in SE-DRC (only men) and of 7.3% in Zanzibar (all men except one woman), primarily from the rural sites. Self-employment (that is, informal business, petty trading, and so on) was also an important occupation reported by more than 17% across all settings. Mean age was comparable across settings, varying between 32.8 and 38.8 years. Men were on average older than women in SE-DRC and Zanzibar, and rural people in western Kenya were older than urban people. Average household size was lowest in western Kenya (mean of 4.5) and higher in SE-DRC (6.2) and Zanzibar (6.8). Respondents in western Kenya and SE-DRC were predominantly Christian and respondents in Zanzibar were Muslim. Between 69% (Zanzibar) and 88% (western Kenya, SE-DRC) reported to have completed primary or secondary school. Men and urban respondents were better educated across all settings. A reliable income was reported least in SE-DRC (35.3%), and higher in western Kenya (47.8%) and Zanzibar (55.9%).

### Identification, perceived seriousness, fatality and past experience of cholera

The condition described in the cholera vignette was identified as cholera in local terms by 96% of respondents in SE-DRC, by 75% in western Kenya and by 88% in Zanzibar (*P* <0.001) (Table 1). There were no gender differences in illness recognition, and only a site difference in SE-DRC and Zanzibar. Almost every respondent in SE-DRC was afraid of death due to cholera; fewer respondents in western Kenya (50%) and Zanzibar (78%) expected a fatal outcome (*P* <0.001). Cholera was reported as ‘very serious illness’ by >8 out of 10 respondents in all settings. Almost half of the interviewed people in SE-DRC (44.4%) reported having witnessed a cholera episode within the household, and more so in the urban site. Among the 22.4% of respondents reporting this in western Kenya, urban residents and men were more prevalent; there was no gender difference in SE-DRC and Zanzibar. More rural residents were among the 15.5% who reported household episodes in Zanzibar.

**Table 1:** Identification, seriousness, fatality and past episodes of cholera in endemic areas of three African settings

**Category**	**SE-DRC, n = 360**			**Western Kenya, n = 379**			**Zanzibar, n = 356**		
Overall	%			%			%		
Identification of illness^a^***	96.4			75.2			88.2		
Seriousness^b^***	81.1			91.3			96.6		
Expected fatal outcome without treatment^c^***	99.7			49.9			77.5		
Past household episodes***	44.4			22.4			15.5		
Past personal episodes*	8.3			11.6			5.3		
Site comparison	Urban, %	Rural, %	*P* value	Urban, %	Rural, %	*P* value	Urban, %	Rural, %	
									*P* value
Identification of illness^a^	**93.9**	**98.9**	**0.020**	72.6	77.8	0.285	**95.5**	**80.8**	**<0.001**
Seriousness^b^	**85.6**	**76.7**	**0.030**	91.6	91.0	0.832	95.0	98.3	0.084
Expected fatal outcome without treatment^c^	99.4	100.0	0.317	**60.0**	**39.7**	**<0.001**	**84.4**	**70.6**	**0.002**
Past household episodes	**52.2**	**36.7**	**0.004**	**27.4**	**17.5**	**0.044**	**6.7**	**24.3**	**<0.001**
Past personal episodes	7.7	8.9	0.703	13.2	10.1	0.423	**2.8**	**7.9**	**0.035**
Gender comparison	Female, %	Male, %	*P* value	Female, %	Male, %	*P* value	Female, %	Male, %	
									*P* value
Identification of illness^a^	97.8	95.0	0.170	78.8	71.5	0.122	88.3	88.1	>0.999
Seriousness^b^	79.0	83.2	0.295	91.2	91.4	0.907	96.1	97.2	0.577
Expected fatal outcome without treatment^c^	99.5	100.0	0.320	52.9	46.8	0.151	74.9	80.2	0.257
Past household episodes	43.1	45.8	0.563	**15.5**	**29.6**	**0.003**	12.9	18.1	0.172
Past personal episodes	7.8	8.9	0.680	**8.3**	**15.1**	**0.053**	3.4	7.3	0.104

‘Overall’: comparison between settings based on the χ^2^ test (identification of illness and personal episodes) and Kruskal-Wallis test (seriousness, fatality and household episodes), *P <0.05, ***P <0.001. ‘Site comparison’ and ‘Gender comparison’: figures in bold designate significant differences at *P* <0.05 based on the Fisher’s exact test (identification of illness and personal episodes) and Wilcoxon test (seriousness, fatality and household episodes). Data for Southeastern Democratic Republic of Congo (SE-DRC) in ‘Overall’ section from Merten *et al*. [31]. Data for western Kenya in ‘Site comparison’ section from Nyambedha *et al. *[28].

Data for Zanzibar in ‘Overall’ and ‘Site comparison’ sections from Schaetti *et al. *[27].

^a^Identified as cholera in local language based on vignette.

^b^Coded as ‘very serious’.

^c^Coded as ‘usually fatal without treatment’.

### Patterns of distress: priority symptoms and psychosocial impact

Excluding symptoms that were mentioned in the vignette (that is, muscle cramps, vomiting, frequent and large amounts of rice-water-like stool), weakness was identified as the most prominent symptom in all settings (see Additional file 4). Unconsciousness (SE-DRC, Zanzibar) and sunken eyes (all three settings), which are signs of dehydration, were also prominently reported. Somatic symptoms were mainly differentiated between urban and rural sites in SE-DRC and Zanzibar. In SE-DRC, where urban community residents have more experience with cholera, signs of dehydration and rectal pain received greater prominence than in the rural site. In western Kenya, lack of awareness about additional symptoms for cholera, coded as ‘cannot say,’ was significantly higher than in SE-DRC and Zanzibar, and with a higher prominence in the rural site.

Cholera was perceived as having a significant impact on people’s lives in all three settings (Table 2). The social impact of cholera was mainly characterized by financial concerns that were manifested by people reporting loss of family income and interference with work-related activities in all three settings, albeit with significantly differing prominences. Men in SE-DRC and Zanzibar reported more negative influences of cholera on the household economy. In western Kenya, direct costs related to cholera episodes were not a priority overall, but a distinct urban concern.

**Table 2:** Comparison of the psychosocial impact of cholera in endemic areas of three African settings, by site and gender

**Category**	**SE-DRC, n = 360**					**Western Kenya, n = 379**					**Zanzibar, n = 356**				
Overall	Total reported		Prominence			Total reported		Prominence			Total reported		Prominence		
Interference with work/daily activities***	**96.9**		**1.64**			**98.7**		**2.19**			**96.9**		**2.35**		
Loss of family income***	**92.5**		**2.41**			**96.3**		**2.63**			**95.5**		**2.13**		
Sadness, anxiety, worry***	**97.8**		**2.70**			**98.2**		**1.84**			**97.5**		**1.89**		
Site comparison	Urban		Rural			Urban		Rural			Urban		Rural		
	Total reported	Prominence	Total reported	Prominence	*P* value	Total reported	Prominence	Total reported	Prominence	*P* value	Total reported	Prominence	Total reported	Prominence	*P* value
Costs (transport, food, drugs)	87.8	1.40	94.4	1.42	0.575	**97.9**	**1.51**	**87.3**	**1.28**	**<0.001**	97.2	2.07	96.0	1.67	0.142
Disruption of health services	68.9	0.96	71.7	0.84	0.229	55.3	0.57	51.9	0.58	0.627	**48.0**	**0.54**	**88.1**	**0.94**	**<0.001**
Interference with social relationships	**67.2**	**1.12**	**86.7**	**1.56**	**<0.001**	84.2	1.35	78.8	1.33	0.379	**65.4**	**0.82**	**74.6**	**1.28**	**<0.001**
Loss of family income	91.1	2.57	93.9	2.25	0.174	**97.9**	**2.94**	**94.7**	**2.31**	**<0.001**	98.3	2.11	92.7	2.16	0.463
Sadness, anxiety, worry	**97.8**	**2.43**	**98.3**	**2.96**	**<0.001**	100.0	1.90	96.3	1.78	0.064	**100.0**	**2.06**	**94.9**	**1.72**	**<0.001**
Gender comparison	Female		Male		*P* value	Female		Male		*P* value	Female		Male		*P* value
	Total reported	Prominence	Total reported	Prominence		Total reported	Prominence	Total reported	Prominence		Total reported	Prominence	Total reported	Prominence	
Disruption of health services	69.6	0.88	70.9	0.92	0.754	**46.9**	**0.47**	**60.5**	**0.69**	**0.004**	73.2	0.78	62.7	0.69	0.051
Interference with work/daily activities	**95.0**	**1.46**	**98.9**	**1.82**	**0.005**	98.5	2.07	98.9	2.32	0.085	95.5	2.36	97.2	2.33	0.704
Loss of family income	94.5	2.55	90.5	2.27	0.112	96.4	2.74	96.2	2.51	0.178	**93.9**	**1.96**	**97.7**	**2.31**	**0.007**

Categories ordered alphabetically, except for ‘cannot say’. Total reported = percentage of categories reported spontaneously and upon probing. ‘Prominence’ = mean prominence of categories based on how reported (spontaneous = 2, probed = 1, most troubling = 3). ‘Overall’: figures in bold designate top three prominent categories; comparison between settings based on the Kruskal-Wallis test, *** *P* <0.001. ‘Site comparison’ and ‘Gender comparison’: figures in bold designate significant differences at *P* <0.05 based on the Wilcoxon test. Data for Southeastern Democratic Republic of Congo (SE-DRC) in ‘Overall’ section from Merten *et al*. [31]. Data for western Kenya in ‘Site comparison’ section from Nyambedha *et al*. [28]. Data for Zanzibar in ‘Site comparison’ section from Schaetti *et al.*[27].

### Perceived causes

Problems with unsafe drinking water and a dirty environment in general, were the most common perceived causes for cholera in all settings (Table 3). However, locally embedded explanations (for example, witchcraft) were still widespread, especially in rural areas (SE-DRC and Zanzibar). Lack of latrines was also a prominent perceived cause in western Kenya (not elicited in Zanzibar). There were site differences in 10 out of 12 categories in Zanzibar, while only 3 categories in SE-DRC and 4 categories in western Kenya had significant urban/rural differences. A dirty environment was often reported more in urban sites of western Kenya and Zanzibar, which may reflect conditions that residents are unable to control. Flies were particularly prominent in Zanzibar, without differentiation between site and gender. Flies, which can act as disease vectors for cholera, were mostly mentioned in connection with food handling in Zanzibar.

**Table 3:** Comparison of perceived causes for cholera in endemic areas of three African settings, by site and gender

**Category**	**SE-DRC, n = 360**					**Western Kenya, n = 379**					**Zanzibar, n = 356**				
Overall	Total reported		Prominence			Total reported		Prominence			Total reported		Prominence		
Dirty environment***	**93.3**		**1.83**			**96.3**		**1.97**			**97.8**		**2.99**		
Drinking contaminated water***	**94.7**		**2.68**			**95.5**		**2.21**			**95.2**		**1.65**		
Eating unprotected/spoiled food***	**94.7**		**1.94**			93.1		1.75			94.9		1.44		
Flies*	95.3		1.44			96.0		1.30			**96.9**		**1.60**		
Lack of latrines^a^***	93.3		1.72			**95.8**		**1.79**			NA		NA		
Site comparison	Urban		Rural		*P* value	Urban		Rural		*P* value	Urban		Rural		*P* value
	Total reported	Prominence	Total reported	Prominence		Total reported	Prominence	Total reported	Prominence		Total reported	Prominence	Total reported	Prominence	
Contact with contaminated water	84.4	1.17	89.4	1.12	0.657	55.8	0.64	63.0	0.75	0.115	**85.5**	**1.07**	**91.0**	**1.58**	**<0.001**
Dirty environment	91.7	1.83	95.0	1.82	0.631	**97.9**	**2.29**	**94.7**	**1.63**	**<0.001**	**99.4**	**3.68**	**96.0**	**2.30**	**<0.001**
Drinking contaminated water	**93.3**	**2.34**	**96.1**	**3.02**	**<0.001**	97.4	2.18	93.7	2.23	0.778	96.1	1.65	94.4	1.66	0.384
Eating forbidden food	20.6	0.23	13.3	0.15	0.094	11.6	0.12	14.8	0.15	0.365	**27.4**	**0.27**	**54.8**	**0.48**	**<0.001**
Eating soil	**53.9**	**0.65**	**41.7**	**0.45**	**0.006**	60.0	0.60	52.4	0.52	0.136	**36.9**	**0.37**	**48.6**	**0.49**	**0.023**
Eating unprotected/spoiled food	92.8	1.89	96.7	1.99	0.100	95.3	1.75	91.0	1.74	0.432	**95.5**	**1.60**	**94.4**	**1.27**	**<0.001**
Flies	93.3	1.37	97.2	1.52	0.236	**96.3**	**1.35**	**95.8**	**1.25**	**0.004**	99.4	1.62	94.4	1.58	0.163
God’s will	40.0	0.56	42.2	0.55	0.668	8.9	0.09	7.4	0.07	0.586	**93.3**	**1.22**	**86.4**	**1.83**	**0.001**
Malaria	26.1	0.30	20.0	0.21	0.115	19.5	0.21	24.9	0.25	0.243	**15.1**	**0.15**	**48.0**	**0.49**	**<0.001**
Witchcraft	**47.8**	**0.64**	**69.4**	**0.86**	**<0.001**	9.5	0.09	11.6	0.12	0.494	**20.7**	**0.21**	**45.8**	**0.50**	**<0.001**
Worms	36.1	0.39	36.7	0.39	0.976	**23.2**	**0.24**	**39.7**	**0.40**	**0.001**	**13.4**	**0.13**	**46.9**	**0.47**	**<0.001**
Cannot say	4.4	0.11	3.9	0.14	0.638	**2.1**	**0.42**	**11.6**	**0.77**	**0.005**	**1.1**	**0.02**	**13.6**	**0.27**	**<0.001**
Gender comparison	Female		Male		*P* value	Female		Male		*P* value	Female		Male		*P* value
	Total reported	Prominence	Total reported	Prominence		Total reported	Prominence	Total reported	Prominence		Total reported	Prominence	Total reported	Prominence	
Dirty environment	92.3	1.77	94.4	1.89	0.251	**96.9**	**1.84**	**95.7**	**2.10**	**0.043**	97.8	2.91	97.7	3.07	0.334
Eating soil	51.9	0.61	43.6	0.49	0.092	**45.4**	**0.45**	**67.6**	**0.68**	**<0.001**	43.6	0.44	41.8	0.42	0.772
Cannot say	5.0	0.15	3.4	0.10	0.620	7.7	0.65	5.9	0.54	0.476	**11.2**	**0.22**	**3.4**	**0.07**	**0.005**

Categories ordered alphabetically, except for ‘cannot say’. ‘Total reported’ = percentage of categories reported spontaneously and upon probing. ‘Prominence’ = mean prominence of categories based on how reported (spontaneous = 2, probed = 1, most important = 3). ‘Overall’: figures in bold designate top three prominent categories; comparison between settings based on the Kruskal-Wallis test, *P <0.05, ***P <0.001. ‘Site comparison’ and ‘Gender comparison’: figures in bold designate significant differences at *P* <0.05 based on the Wilcoxon test. Data for Southeastern Democratic Republic of Congo (SE-DRC) in ‘Overall’ section from Merten *et al*. [31]. Data for western Kenya in ‘Site comparison’ section from Nyambedha *et al. *[28]. Data for Zanzibar in ‘Site comparison’ section from Schaetti *et al. *[27].

^a^Not elicited in Zanzibar.

### Knowledge of prevention and priority of hygiene and sanitation

Safe food and water and vaccines were prioritized for prevention in SE-DRC (Table 4). Although safe water was also a priority in western Kenya, respondents in western Kenya and Zanzibar identified sanitation issues (stool and garbage disposal) as priorities for prevention. Health education was reported with equal priority across all settings (*P* = 0.925). Differing ideas on prevention between sites and between men and women existed mainly in western Kenya, and least often in SE-DRC. Vaccination as a preference was not reported differently between sites and gender.

**Table 4:** Comparison of prevention options for cholera in endemic areas of three African settings, by site and gender

**Category**	**SE-DRC, n = 360**					**Western Kenya, n = 379**					**Zanzibar, n = 354**				
Overall	Total reported		Prominence			Total reported		Prominence			Total reported		Prominence		
Health education	92.5		1.75			**98.9**		**1.89**			**95.5**		**1.90**		
Safe disposal of garbage***	95.0		1.79			97.9		1.61			**98.6**		**2.09**		
Safe disposal of stool***	93.9		1.53			**97.9**		**1.81**			**98.3**		**2.09**		
Safe food***	**95.3**		**1.92**			98.4		1.67			97.5		1.66		
Safe water***	**93.3**		**2.06**			**98.2**		**1.93**			96.9		1.74		
Vaccines***	**87.2**		**2.00**			87.9		1.15			86.2		1.20		
Site comparison	Urban		Rural			Urban		Rural			Urban		Rural		
	Total reported	Prominence	Total reported	Prominence	*P* value	Total reported	Prominence	Total reported	Prominence	*P* value	Total reported	Prominence	Total reported	Prominence	*P* value
Health education	90.0	1.80	95.0	1.71	0.371	**98.9**	**2.27**	**98.9**	**1.51**	**<0.001**	96.6	1.93	94.3	1.88	0.581
Preventive drugs	59.4	0.87	65.0	1.04	0.331	**86.3**	**1.05**	**91.5**	**1.20**	**0.049**	**83.7**	**1.01**	**88.6**	**1.18**	**0.040**
Protection from supernatural influence^a^	**10.6**	**0.13**	**5.0**	**0.05**	**0.047**	**7.4**	**0.09**	**21.7**	**0.22**	**<0.001**	NA	NA	NA	NA	NA
Safe disposal of garbage	93.9	1.71	96.1	1.87	0.154	98.4	1.53	97.4	1.69	0.257	**99.4**	**2.51**	**97.7**	**1.66**	**<0.001**
Safe disposal of stool	91.7	1.53	96.1	1.54	0.911	**97.4**	**1.66**	**98.4**	**1.96**	**0.026**	99.4	1.96	97.2	2.21	0.301
Safe food	94.4	1.86	96.1	1.98	0.298	**98.9**	**1.76**	**97.9**	**1.58**	**0.002**	**98.3**	**1.78**	**96.6**	**1.53**	**0.001**
Gender comparison	Female		Male			Female		Male			Female		Male		
	Total reported	Prominence	Total reported	Prominence	*P* value	Total reported	Prominence	Total reported	Prominence	*P* value	Total reported	Prominence	Total reported	Prominence	*P* value
Health education	91.7	1.66	93.3	1.84	0.361	98.5	1.87	99.5	1.92	0.571	**95.5**	**1.72**	**95.4**	**2.10**	**0.014**
Protection from supernatural influence^a^	**5.0**	**0.05**	**10.6**	**0.13**	**0.044**	13.4	0.14	15.7	0.17	0.533	NA	NA	NA	NA	NA
Safe disposal of garbage	95.6	1.78	94.4	1.79	0.500	**97.4**	**1.52**	**98.4**	**1.70**	**0.026**	98.9	2.13	98.3	2.05	0.906
Safe food	94.5	1.98	96.1	1.86	0.206	98.5	1.69	98.4	1.65	0.888	**98.3**	**1.78**	**96.6**	**1.53**	**0.013**
Safe water	92.3	2.15	94.4	1.98	0.159	**98.5**	**2.04**	**97.8**	**1.82**	**0.019**	97.2	1.77	96.6	1.71	0.926

Categories ordered alphabetically. ‘Total reported’ = percentage of categories reported spontaneously and upon probing. ‘Prominence’ = mean prominence of categories based on how reported (spontaneous = 2, probed = 1, most useful = 3). ‘Overall’: figures in bold designate top three prominent categories; comparison between settings based on the Kruskal-Wallis test, *** *P* <0.001. ‘Site comparison’ and ‘Gender comparison’: figures in bold designate significant differences at *P* <0.05 based on the Wilcoxon test. Data for Southeastern Democratic Republic of Congo (SE-DRC) in ‘Overall’ section from Merten *et al*. [31]. Data for western Kenya in ‘Site comparison’ section from Nyambedha *et al. *[28].

^a^Not elicited in Zanzibar.

### Help-seeking behavior

Use of ORS and rehydration in general was a top priority for home-based treatment of cholera patients in all three settings (Table 5). Self-administration of drugs was an additional prominent treatment option in SE-DRC (mainly antibiotics) and western Kenya (tetracycline and metronidazole (Flagyl®) most frequently mentioned). Herbal treatment was the most prominent option in Zanzibar, with a rural preference. Less commonly reported practices were praying, which showed a higher prominence in urban than rural western Kenya, and drinking alcohol. The latter category, which was not elicited in the Muslim society of Zanzibar, was higher among men than women in SE-DRC and western Kenya.

**Table 5:** Comparison of home-based self-treatment options for cholera in endemic areas of three African settings, by site and gender

**Category**	**SE-DRC, n = 360**					**Western Kenya, n = 379**					**Zanzibar, n = 356**				
Overall	Total reported		Prominence			Total reported		Prominence			Total reported		Prominence		
Drinking water/liquids***	**55.6**		**0.86**			**89.4**		**2.00**			**69.1**		**1.32**		
Herbal treatment***	50.3		0.84			33.2		0.62			**66.3**		**1.79**		
ORS***	**92.8**		**3.26**			**87.1**		**2.20**			**66.3**		**1.45**		
Self-administered drugs***	**56.1**		**1.19**			**73.9**		**1.89**			58.4		1.30		
Site comparison	Urban		Rural		*P* value	Urban		Rural		*P* value	Urban		Rural		*P* value
	Total reported	Prominence	Total reported	Prominence		Total reported	Prominence	Total reported	Prominence		Total reported	Prominence	Total reported	Prominence	
Doing nothing at home	5.6	0.09	10.6	0.24	0.109	**0.5**	**0.03**	**5.3**	**0.12**	**0.020**	**27.9**	**1.25**	**19.8**	**0.53**	**0.005**
Drinking water/liquids	**66.7**	**1.07**	**44.4**	**0.64**	**<0.001**	87.9	1.93	91.0	2.07	0.493	**68.7**	**1.58**	**69.5**	**1.05**	**0.006**
Herbal treatment	50.0	0.79	50.6	0.89	0.912	30.0	0.61	36.5	0.63	0.292	**49.7**	**1.29**	**83.1**	**2.31**	**<0.001**
Prayers	44.4	0.80	50.6	0.74	0.497	**51.1**	**0.68**	**32.3**	**0.37**	**<0.001**	55.9	0.74	47.5	0.73	0.229
Self-administered drugs	**48.3**	**1.03**	**63.9**	**1.36**	**0.006**	76.8	1.95	70.9	1.83	0.251	**44.7**	**1.03**	**72.3**	**1.57**	**<0.001**
Gender comparison	Female		Male		*P* value	Female		Male		*P* value	Female		Male		*P* value
	Total reported	Prominence	Total reported	Prominence		Total reported	Prominence	Total reported	Prominence		Total reported	Prominence	Total reported	Prominence	
Alcoholic drink^a^	**4.4**	**0.05**	**11.2**	**0.12**	**0.019**	**4.1**	**0.06**	**10.8**	**0.14**	**0.014**	NA	NA	NA	NA	NA
Herbal treatment	47.0	0.78	53.6	0.91	0.147	**25.8**	**0.42**	**41.1**	**0.83**	**0.001**	70.9	1.92	61.6	1.67	0.149
ORS	91.7	3.27	93.9	3.26	0.790	**90.2**	**2.38**	**83.8**	**2.01**	**0.017**	65.4	1.36	67.2	1.53	0.492
Self-administered drugs	**50.3**	**1.07**	**62.0**	**1.32**	**0.023**	69.6	1.87	78.4	1.91	0.585	61.5	1.35	55.4	1.25	0.363

Categories ordered alphabetically. ‘Total reported’ = percentage of categories reported spontaneously and upon probing. ‘Prominence’ = mean prominence of categories based on how reported (spontaneous = 2, probed = 1, most helpful = 3). ‘Overall’: Figures in bold designate top three prominent categories; comparison between settings based on the Kruskal-Wallis test, *** *P* <0.001. ‘Site comparison’ and ‘Gender comparison’: Figures in bold designate significant differences at *P* <0.05 based on the Wilcoxon test. Data for Southeastern Democratic Republic of Congo (SE-DRC) in ‘Overall’ section from Merten *et al*. [31]. Data for western Kenya in ‘Site comparison’ section from Nyambedha *et al. *[28]. Data for Zanzibar in ‘Site comparison’ section from Schaetti *et al. *[27].

^a^Not elicited in Zanzibar.

*ORS* oral rehydration solution.

Healthcare facilities were universally mentioned in all three settings (Table 6), with an urban preference in western Kenya and Zanzibar and a rural preference in SE-DRC based on the assessment of the most preferred health provider. Other help-seeking practices were far less common: advice from friends and colleagues was the second most prominent category in western Kenya and Zanzibar, also preferred more by rural and male respondents. Visiting pharmacies/or purchasing over-the-counter drugs was the third most prominent category across all settings; it had the highest priority in western Kenya and was reported with a significantly higher prominence in rural than in urban Zanzibar.

**Table 6:** Comparison of outside help-seeking options for cholera in endemic areas of three African settings, by site and gender

**Category**	**SE-DRC, n = 360**					**Western Kenya, n = 379**					**Zanzibar, n = 356**				
Overall	Total reported		Prominence			Total reported		Prominence			Total reported		Prominence		
Faith healers	**19.4**		**0.31**			18.5		0.23			14.9		0.18		
Healthcare facility	**100.0**		**4.79**			**100.0**		**4.76**			**100.0**		**4.64**		
Informal help***	13.6		0.18			**54.6**		**0.72**			**55.9**		**0.86**		
Pharmacy/OTC***	**19.4**		**0.20**			**59.1**		**0.70**			**34.0**		**0.36**		
Site comparison	Urban		Rural		*P* value	Urban		Rural		*P* value	Urban		Rural		*P* value
	Total reported	Prominence	Total reported	Prominence		Total reported	Prominence	Total reported	Prominence		Total reported	Prominence	Total reported	Prominence	
Healthcare facility	**100.0**	**4.69**	**100.0**	**4.90**	**0.001**	**100.0**	**4.87**	**99.5**	**4.65**	**0.005**	**100.0**	**4.87**	**100.0**	**4.41**	**<0.001**
Informal help	15.6	0.22	11.7	0.13	0.263	**44.2**	**0.54**	**65.1**	**0.90**	**<0.001**	**38.5**	**0.52**	**73.4**	**1.21**	**<0.001**
Pharmacy/OTC	20.6	0.22	18.3	0.18	0.546	55.3	0.61	63.0	0.79	0.072	**27.4**	**0.27**	**40.7**	**0.44**	**0.007**
Traditional healers	9.4	0.14	4.4	0.09	0.075	12.1	0.16	17.5	0.19	0.187	**3.9**	**0.04**	**9.6**	**0.12**	**0.031**
Gender comparison	Female		Male		*P* value	Female		Male		*P* value	Female		Male		*P* value
	Total reported	Prominence	Total reported	Prominence		Total reported	Prominence	Total reported	Prominence		Total reported	Prominence	Total reported	Prominence	
Healthcare facility	100.0	4.77	100.0	4.82	0.976	100.0	4.79	100.0	4.72	0.475	**100.0**	**4.77**	**100.0**	**4.51**	**0.013**
Informal help	10.5	0.12	16.8	0.23	0.078	**49.5**	**0.62**	**60.0**	**0.83**	**0.026**	**50.8**	**0.69**	**61.0**	**1.03**	**0.014**
Traditional healers	5.0	0.08	8.9	0.15	0.159	**10.3**	**0.12**	**19.5**	**0.22**	**0.014**	7.8	0.08	5.6	0.07	0.418

Categories ordered alphabetically. ‘Total reported’ = percentage of categories reported spontaneously and upon probing. ‘Prominence’ = mean prominence of categories based on how reported (spontaneous = 2, probed = 1, most helpful = 3). ‘Overall’: figures in bold designate top three prominent categories; comparison between settings based on the Kruskal-Wallis test, *** *P* <0.001. ‘Site comparison’ and ‘Gender comparison’: figures in bold designate significant differences at *P* <0.05 based on the Wilcoxon test. Data for Southeastern Democratic Republic of Congo (SE-DRC) in ‘Overall’ section from Merten *et al*. [31]. Data for western Kenya in ‘Site comparison’ section from Nyambedha *et al. *[28]. Data for Zanzibar in ‘Site comparison’ section from Schaetti *et al. *[27].

*OTC* over-the-counter drugs.

### Vulnerability to the illness

Respondents in SE-DRC reported cholera as a condition affecting almost everybody (>84%) without differentiating much between sex, age and class (Table 7). This percentage was a little lower in Zanzibar (>74%). Respondents in western Kenya differentiated more between women and men and identified children and the poor as most vulnerable to cholera.

**Table 7:** Perceived vulnerability to cholera in endemic areas of three African settings

**Category**	**SE-DRC, n = 360**	**Western Kenya, n = 379**	**Zanzibar, n = 356**
Sex			
No differentiation	90.6	63.6	87.1
Women more vulnerable	6.9	24.5	10.4
Men more vulnerable	2.5	11.9	2.5
Age			
No differentiation	84.4	26.9	79.5
Adults more vulnerable	8.1	22.4	8.1
Children more vulnerable	7.5	50.7	12.4
Class			
No differentiation	86.3	44.9	74.4
Rich more vulnerable	0.6	2.9	0.0
Poor more vulnerable	13.1	52.2	25.6

*SE-DRC* Southeastern Democratic Republic of Congo.

## Discussion

Based on data from almost 1,100 interviews in 3 endemic settings, this paper represents the first systematic study of the nature and distribution of local sociocultural features of cholera in urban and rural communities at high risk in eastern Africa. The following points may be worth considering in planning local educational activities to increase public awareness of interventions for cholera control, and to advise communities of practical ways of preventing cholera and managing cases. Study findings may also be used to promote advocacy among decision makers for investment in strategies and action for better control or elimination of cholera.

### Implications for regional cholera control policy and action

This study identified more differences between urban and rural communities than between men and women across all domains. This suggests a need for an approach in program planning that is sensitive to setting-specific disparities in all three settings. Findings also indicate that local terms for cholera are recognized and adequately understood in all settings by over three-quarters of the surveyed populations. Use of these terms in health education and control activities is advisable.

There is a past collective experience and memory of cholera-related symptoms in all settings. In SE-DRC, where poverty levels are higher and the public health system is weaker than the other settings, people rely heavily on social networks since income-generating activities are less available. Thus, social networks may be more important in SE-DRC (and probably also in DRC in general) in order to meet needs. Cholera causes primarily a considerable economic impact in all settings represented by fears of absence from work or income-generating activities and reported loss of family income. Indirect costs were reported more than direct costs, probably reflecting the fact that the latter are usually covered by the healthcare system (during CTCs) or by non-governmental organizations in all three settings. In Zanzibar, laboratory-confirmed cholera patients reported almost three-quarters of private costs as indirect costs [19]. Differences in costs anticipated between rural and urban areas (different options for generating income) and genders (men more often generating a cash income) were not consistent across all settings.

In line with the high recognition of the clinical vignette and the reported severity and help seeking, infectious pathways of cholera are widely acknowledged, even though local causal explanations continue to coexist in all settings. The predominance of environmental and sanitation-related factors and ingestion of contaminated water or food hygiene as perceived causes points to interventions needed in infrastructure. Because of this relatively high overlap between biomedical facts and local ideas about cholera, future community interventions may preferably address environmental and infrastructural issues, rather than solely reemphasize education about causes of cholera. It cannot be ruled out, however, that for those who subscribe to traditional causes such as sorcery (primarily in DRC), classical control activities may not be sufficient.

The three settings differ considerably in terms of water supply and sanitation infrastructure. Use of improved drinking water sources (45%) and improved sanitation (24%) was lowest in 2010 in DRC [13]; the same indicators were higher in Kenya (59%, 32%) and Zanzibar (53%, 53%; numbers for Tanzania). Despite this relative variation, but in line with the commonly prioritized environmental and water and food-related perceived causes, the most prominent prevention options referred to infrastructural aspects (water and sanitation) in all settings.

Similar to the prominence of biomedical meanings, overall help seeking at home reflected a relatively high awareness about professional cholera treatment in all three settings. Rehydration to treat patients at home was prominently reported in all three settings. Antibiotics were the third most prominent treatment option in SE-DRC and western Kenya. They were more prominent in rural areas in SE-DRC and Zanzibar, even though rural villagers are generally poorer and antibiotics have to be purchased. This may be a consequence of limited access to health facilities in the rural areas, while at the same time indicating the availability of antibiotics in remote areas. This suggests that future interventions should primarily focus on rehydration, but also reconsider the role of antibiotics and their potential of being used inappropriately. Antibiotics are part of the treatment regimen as the WHO recommends antibiotics for severe cases [55] (though some recommend it also for moderate cases [56]), but indiscriminate use may jeopardize their effectiveness and was shown to induce resistance [57]. Antibiotic use also means out-of-pocket expenditures that may be better used for purchasing ORS packets or salt and sugar for home-made oral rehydration solutions.

Herbal treatment, which is still important today for diarrhea management in many African settings despite westernization and modernization, may potentially conflict with effective cholera treatment. Herbs were among the most important self-help options in rural Zanzibar; this may call for more emphasis about their potential to delay initiation of rehydration and the use of ORS. The lower priority for herbal treatment in SE-DRC and western Kenya may suggest less attention is needed in that regard for effective information, health education and communication campaigns.

While limited accessibility to health posts or CTCs may still hinder patients or caretakers from seeking care for cholera, the consistent and pronounced priority for professional treatment of cholera across the three settings is another important finding for policy makers in the region. The underutilization of health services may be explained by various factors, such as distance, perceived quality of care, competing obligations, recognition of a need for treatment, and so on. Further study of the role of these reasons locally would be relevant for cholera control.

Concerning questions about the vulnerability of some segments of the population to cholera, in western Kenya respondents were more likely to acknowledge differences. They were consistently less likely than respondents in the other settings to report ‘no differentiation,’ and they more frequently identified women or men, adults or children, and the poor as more vulnerable. The findings suggest both greater cultural sensitivity to vulnerability in general, and a tendency to generalize the vulnerability of women and children to cholera. Respondents at the other settings were less likely to distinguish the vulnerability of these specific subgroups. In the comparison of SE-DRC and western Kenya, this may reflect less access to health services and lower levels of overall development, which affect everyone. In Zanzibar, the finding may reflect less emphasis on vulnerability in health policy. However, this is unclear and the reasons for acknowledging the vulnerability of some groups require further study. In any case, the relative priority of the needs of the general population and of specific subgroups requires consideration in cholera control and for strategies to integrate services in the general health system. Acknowledgement of children and pregnant women as high-risk groups that should be prioritized for cholera vaccines [58] suggest more attention to the relative vulnerability of subgroups would be appropriate in health education in SE-DRC and Zanzibar.

Vaccination has also been recommended by the WHO as an additional measure to WASH in epidemic and endemic situations [16]. Although no vaccination campaigns have been conducted in the study sites before the surveys, the use of vaccines for prevention was identified by a majority in all settings and it received the highest priority in SE-DRC. Vaccine action was a matter of sufficient priority for the government of Zanzibar that it conducted an OCV mass vaccination campaign in 2009. Additional analyses on community demand for oral cholera vaccination confirm a high regard for vaccination with anticipated acceptance rates above 93% in the three surveys [29-31]. While an analysis of sociocultural determinants of anticipated OCV acceptance across the three settings using meta-analytical techniques is in preparation, the descriptive findings presented here indicate good prospects for future vaccination campaigns in the region. It should be noted, however, that a high acceptability, constituting only one component of access to health (care), does not directly relate to a high effectiveness. Unlike other multicountry comparisons of people’s ideas about illness and willingness to receive a not-yet-existing vaccine [59,60], finhdings from this study are directly relevant for public health practice. Two cholera vaccines are available and prequalified by the WHO, and planning is underway to increase their use in populations at risk [61].

This study is limited by the fact that findings are based on cross-sectional surveys, which do not take into account the possibility of changes in the studied domains over time. The strengths of this three-country comparison lie in the use of three individual community surveys that were planned and implemented in a highly consistent manner. All surveys used the same, although locally adapted, interview schedule; this enabled maximum comparability in the analysis of sociocultural features of cholera across the three settings.

## Conclusions

Based on this comprehensive review of local understandings of cholera-related diarrhea and priorities for cholera control in southeastern Democratic Republic of Congo, western Kenya and Zanzibar, local program planners are encouraged to intensify control activities in this region. Sustainable cholera control, let alone elimination, is only possible through improvements in the local water supply and sanitation infrastructure. Due to political and economic realities in the region, which are improving much too slowly, control of cholera continues to depend mostly on response activities (that is, ensuring timely rehydration through local treatment centers) in the foreseeable future. This study indicates that such an approach is likely to be very effective in areas with endemic cholera in eastern Africa. At the same time, regional decision makers may also consider using vaccines in populations at risk of recurrent cholera outbreaks; such intermediate activities would help mitigate morbidity and mortality while programs for improving water and sanitation are underway.

## Competing interests

The authors declare that they have no competing interests.

## Authors’ contributions

Conception and design of the study: CS, NS, SM and MGW. Analysis of data: CS, NS and SM. Writing of manuscript: CS, NS, SM and MGW. Revision of manuscript: all authors. All authors read and approved the final manuscript.

## Pre-publication history

The pre-publication history for this paper can be accessed here:

http://www.biomedcentral.com/1741-7015/11/206/prepub

## Supplementary Material

Additional file 1 EMIC interview for study of community views of cholera in southeastern Democratic Republic of Congo. Click here for file

Additional file 2 EMIC interview for study of community views of cholera in western Kenya. Click here for file

Additional file 3 EMIC interview for study of community views of cholera in Zanzibar. Click here for file

Additional file 4 Comparison of priority symptoms for cholera in endemic areas of three African settings, by site and gender. Click here for file
