# Supplementary material for: Comparing sociocultural features of cholera in three endemic African settings
Source: BMC Med. 2013 Sep 18;11:206. doi: 10.1186/1741-7015-11-206 (PMC4016292; doi:10.1186/1741-7015-11-206)
Supplement: Additional file 1 — EMIC interview for study of community views of cholera in southeastern Democratic Republic of Congo. [file 1741-7015-11-206-S1.pdf]

Etude sur les maladies diarrhéiques: catalogue d'interview selon un modèle explicatif (EMIC)

Université de Kinshasa, Département d'anthroplogie

avec le soutien de

l'Organisation Mondiale de la Santé et l'Institut Tropical et de Santé Publique Suisse

Version /swahili/française/cibemba du 28/07/2010 (final template, not enumerated)

**Date de l'interview (jj-mm-aaaa)**		**EMIC ID (RDCX-###)**	RDC __-___ ___ ___
**Heure du début de l'interview**			

Information générale/ubwishibishi bukalamba

Cocher ce qui convient: */ Cocher une case:seulement:*

**Sexe ubwamba**	1 F umwanakashi	2 M umwaume	**Age approximatif (années) imyaka**		**Site icende**	1 urbain Tauni	2 rural Umushi

Introduction

Asante kwa kukubali kuzungumza na mimi leo. Ningependa kukuuliza maswali kuhusu matatizo ya kiafya, ambayo yanaweza kuathiri jamii yako. Inawezekana kwamba unayajua matatizo haya au hujawahi kuyaona. Kwa vyovyote ningependa kujua mawazo yako kuhusu swala hilo. Lakini kwanza ningependa kukuuliza maswali kuhusu maisha yako.

Je vous remercie de m'avoir permis de m'adresser à vous aujourd'hui. Je vais vous poser des questions sur des problèmes de santé qui ont affecté quelques personnes de votre communauté. Il est possible que vous reconnaissiez certains de ces problèmes, mais d'autres peuvent vous être inconnus. Dans les deux cas, je voudrais obtenir votre point de vue à ce sujet. Vos réponses et réflexions, nous permettrons à mieux aider les personnes qui sont affectées par ce problème. Mais d'abord, nous avons quelques questions vous concernant.

Namutotela pakusuminisha ukuti nga mbaule nenu ubushiku bwe lelo. Ndemipusha pa bwafya bwasangilwe mu bumi bwa bantu abo mwikala nabo. Ubwafya bumo bumo na muwishiba lelo bumbi ta mubwishibe. Mu misango yonse ibili ndefwaya ukwishiba ifyo mulentontokanya pali uyu mulandu.Amasuko na matontokanyo yenu kuti ya afwa abantu abali no bwafya. Pantanshi ya fyonse tuli na mepusho kuli mweka

1 Informations socio-économique et démographique

1.1 Hali ya ndoa/ *état civil*/ icupo nangu ubushimbe

Cocher une seule case

1	*2*	*3*	*4*	*5*	*6*	*7*
Sijawahi kuoa au kuolewa/ *Jamais marié*/ sha upa nangu tabangupa	Nimeoa au nimeolewa/ *Marié*/ nalyupa nangu balingupa	Nimetengana / *Séparée*/ twalilekana	Nimeachika/ *Divorcée*/ balindubula	Tunaishi pamoja/ *Union libre/* tulekala pamo	Nimefiwa na mume au mke ndi *Veuf/ve/* mukamfwilwa	Siwezi kusema/ *Ne peut pas dire* teti nande

1.2 Ukubwa wa nyumba/ Taille du ménage/ Ubwingi bwa mu nganda

Idadi ya watu unaishi nao *Nombre de personnes vivant dans le ménage* Ubwingi bwa bantu mu nganda

1.3 Watoto wanaoishi katika nyumba yako/ Nombre d'enfants vivant dans le ménage/ Ubwingi bwa bana balikwikala ku nganda

/ Veuillez noter 0 dans la case si aucune réponse ne correspond/ Lemba 0 mukayanda nga takuli ubwasuko

Idadia ya watoto/ *Nombre d'enfants/* Ubwingi bwa bana	Chini ya miaka 5 / *<5 ans*/ panshi ya myaka 5	Miaka 5 mpaka 10/ *5-10 ans/* kutampa imyaka 5 ukufika imyaka10	Miaka 10 mpaka 17/ *10-17 ans/* ukutampa imyaka 10 ukufika imyaka 17
Wavulana/ *Garçons*/ abaume			
Wasichana/ *Filles*/ abanakashi			

1.4 Uhusiano na msimamizi wa nyumba/ *Relation avec le chef de famille*/ ifyo bali no mukalamba wa nganda

Cocher une case seulement/

1 Mwenyewe *Moi-même* Nebo	2 Mke/mume *époux/se* umulume/ umukashi	3 Wazazi *Parent* abafyashi	4 Ndugu *Frère* lupwa	5 Mtoto *Enfant* umwana	6 Uhusiano mwingine *Autre* umbi

/ Cocher le sexe du chefde famlle

Sexe	1 *Féminin* Umwana-kashi	2 *Masculin* Umwa-ume

1.5 Kazi au ajira/ *Situation professionnelle*/ incito

Cocher une case seulement

1 Kilimo/ *Agriculteur*/ ubulimi	*2* Uvuvi *Pêcheur*/ ukuloba	*3* Nimejiajiri/ *(not 1& 2) Independant (ni 1 ni 2)/* incito ya minwe	*4* Kuajiriwa rasmi *Salarié* /shibuteko	*5* Mama wa nyumbani/ / *ménagère* namayo
6 Mfanyakazi wa nyumbani shi /*Employee de maison/* mayanda nangu na	*7* Kibarua/ /*Tavail occasionel/* kalemba	*8* Mwanafunzi / *étudiant/e* umwana wa masomo	*9* Sifanji kazi/nimestaafu *Retraité / Sans activité professionnelle*/ shibomba/ ndi pasione	*10*Mengine, eleza *Autre à préciser/* incito imbi

1.6 Elimu /*Education/* amasambililo

le niveau d'éducation la plus élevée//

1 Sijasoma/ *Pas d'instruction/* sha sambilila	*2* Elimu ya msingi /*école primaire/* nalekele ku tampilo	*3* Elimu ya sekondari/ *école secondaire/* amasomo ya ku kusekondeli	*4* Elimu amali/ école professionelle/ amsomo ya minwe	*5* Koleji ku *Collège*/ shasambillila	*6* Chuo kikuu *études supérieures et l'universitaires* amasomo ya kulu

1.7 Miaka ya kusoma/ *Nombre d'année d'instruction*/ imyaka ya masambililo

Siwezi kusema/*Ne peut pas dire/* teti nsose

1.8 Dini /*Religion*/ ikilonganino

Cocher une case seulement

1 Mwislamu/ *Musulman*/ ndi musulman	*2* Mkristo/ *Chrétien*/ ndi mu kristu	*3* Mengine, eleza i*:* *Autre à préciser/* kilonganino kimbi *:*______________	*4* Sielezi/ *Ne veut pas se prononcer/* nshishibe

Denomination / autre details: ____

1.9 Uraia*// Nationalité*/ icalo

Cocher une case seulement

1 Kongomani/ *Congolaise*/ ndi mwina kongo	*2* Raia wa nchi nyingine, eleza/ *Autre à préciser*/ ndi mwikashi wa calo kimbi:___________________________________

1.10 Je, kipato cha watu wanaoishi hapa kwako ni cha uhakika? *Le revenu de votre ménage est-il consistant et régulier?* Ni shani amalipo ya bekashi yalilinga no kukonkana*?*

une case seulement, pas divulguée, signifie "pas certain"

Ndio/ Oui / e mukwai *3*	Labda/ Probable / te lyonse *2*	Hakijulikani/ Incertain / tafishibikwe *1*	Hapana/ *Non/* iyoo *0*

Narrative / transcrivez le récit:____

____

____

____

Si "oui" ou "probable" approfondissez comme suit, autrement continuer avec Q 1.12 :

1.11 Chanzo kuu cha mapato hapa kwako ni gani?
Quelles sont les sources princiales de revenu de votre ménage?
Ni munshi mu mwena ulupiya ku nganda yenu ?

transcrivez le récit;____

____

____

____

Cochez tout ce qui convient:

**Sources de revenue**/ umwakukwatila impiya	**/Moi-même/** ne mwine	**Autre membre de la famile/** balupwa
1 Kuajiriwa kwa mshahara/ *Salarié/e/* Shibuteko		
2 Kuajiriwa kwa namna nyingine *Emploie contre "nourriture"/* ndabombelela ifya kulya		
3 Kujiajiri mwenyewe katika sekta yoyote isiyo ya kilimo *Independant mais pas agriculteur/* incito imbi te bulimi		
4 Kuuza mazao ya kilimo ndashitisha ifilimo *Selling agricultural produce* Vente de produits agricoles		
5 Kuuza samaki na mazao ya baharini / *Vente de poisson /* ndashitisha isabi		
6 Kukodisha (nyumba, shamba, duka) /*Location*/ ukusonkesha inganda		
7 Msaada kututoka nje /*Argent reçu de l'extérieur du ménage*/ impiya poka kunse ya nganda		
8 Pensheni /*Rente de Pension/* pasione		
98 Nyinginezo, eleza / */Autre à préciser*/ imisango imbi :__ /______		
99 Siwezi kusema/ *Ne sait pas*/ shishibe		

1.12 Ni pesa ngapi ulitengeneza wewe mwenyewe mwezi uliopita? Na mume wako je? Na watu wengine wanaokaa kwako?
Combien d'argent avez-vous gagné par vos propres moyens le mois dernier? Qu'elle est la situation pour votre conjoint/e ou tout autre membre du ménage?
Ni mpiya shinga mwa kwete ku maka yenu mwe bene mu mwenshi wapitile ni shani ku ba kumwenu nabo

Narrative /Transcrivez le récit/lemba apa: ______________________________

____

____

____

Si il n'y a pas de revenu, entrez 0 dans la colonne FC; en cas de veuvage de la personne cochez " ne peut pas dire" à la question 2

**Revenu mensuel/ Impiya mukwata**	**FC**		
1 Zake mwenyewe<br>*Propre*<br>Shandi		Siwezi kusema<br>*Ne sait pas*<br>Nshishibe	
2 Za mume/mke ambaye bado yu hai<br>*Conjoint/e/*<br>Wiba wandi		Siwezi kusema/nimefiwa<br>*Ne sait pas / veuvage*<br>shishibe /mukamfwilwa	
3 Za watu wengine wanaoishi katika nyumba hii<br>*Revenu supplémentaire du ménage/*<br>Shifuma ku bantu bambi		Siwezi kusema<br>*Ne sait pas*<br>shishibe	

Introduction des vignettes /Tampilo ya fishimi/finyente

Nakushukuru kwa kukubali kuzungumza na mimi kuhusu shida za afya yanayoweza kuathiri jamii. Sasa ningependa kujua maoni yako kuhusu swala hilo. Nataka kuelewa vile unafikiria kuhusu mambo haya ya afya.Ni mawazo yako ninahaja nayo kwa hivyo uwe huru usione haya kunielezea maoni yako. Nitakuambia hadithi mbili tofauti ya watu ambao wana shida mbili tofauti.

Je suis ravie que vous soyez d'accord pour parler de quelques problèmes de santé qui pourraient affecter des personnes de votre communauté. Je voudrais comprendre ce que vous en pensez. Je suis interessé par vos propres idées. Ne soyez donc pas gené à me communiquer vos réponses. Je vais vous raconter deux histoires qui concernent des personnes qui ont deux problèmes de santé différents.

Ndi ne nsansa sana pantu mwasumina ukuti twambaule pa milandu ya bwikashi ku bantu abo mwikala nabo. Ndefwaya ukwishiba ifyo mulentontokanya. Eico mwi umfwa insoni pa kwasuka. Nalamushimikila ifinshimi fibili fya bantu babili abali no bwafya mu bwikashi bwabo.

2 Vignette A (adultes) Ikishimi kya bumo

Sikiliza hadithi ya Musonda/Ngoie ambaye juzi alikuwa na shida ya kiafya…
Laissez-moi vous raconter l'histoire de Musonda/Ngoie…
Umfweni ikishimi kya kwa Musonda/Ngoje uwali mailo nalwala…

2.1 Ugonjwa huu unaitwaje? (Ni jina gani utatumia kuelezea mtu mwingine hali ya ugonwa hii?
Quel est le nom de cette maladie? (quel mot utiliseriez-vous pour décrire cette maladie à quelqu'un d'autre?
Bulwele ki .Lishina ki mupela muntu umbi uulwele

Veuillez spécifier le mot, un résumé des termes ou une brève description reprenant les mots utilisés par le répondant/e. Si "autre" veuillez" préciser le terme et l'expliquer ci-après :

***Narrative / transcrivez le récit lemba apa:**____

____

____

____

**Types de diarrhées**	
1 Kuharisha kawaida /*Diarrhée normale*/ kupolomya	*5* Kipindupindu /*Choléra*/ kolera
2 Kuharisha maji /*Diarrhée acqueuse*/ kupolomya amenshi ayengi	*6* Mchanganyiko / *mixte*/ ifingi
3 Kuharisha marenda /*Diarrhée muqueuse*/ kupolomya ifya lulenda	*98* Mengineyo, eleza /*Autre à préciser*/ bumbi:
4 Kuharisha damu / *Diarrhée sanguinolante*/ kupolomya umulopa	*99* Siwezi kusema / *Ne sais pas*/ shishibe

veuillez coder le nom (une seule réponse possible) de la liste ci-dessus: [ ]

Pour les questions suivantes, veuillez utiliser le nom de la maladie tel que mentionné par la personne en lieu de maladie/problème, et veuillez utiliser le nom de la personne qui figure dans la vignette.

2.2 Je, unaweza kufikiria juu ya dalili zingine ambazo Musonda/Ngoie anaweza kuwa nazo na hazijatajwa hapo juu?
Est-ce que d'autres symptômes vous viennent à l'ésprit que Musonda/Ngoie pourrait présenter en plus de ceux déjà mentionés?
Kuli ifishibilo fimbi ifyo Musonda/Ngoie engakwata ifyo tatulumbwile

Veuillez résumer le réçit du répondant en utilisant ses propres mots : ________________________________

____

.En se basant sur le récit du répondant veuillez cocher ce qui convient sur la colonne Spon, colonne qui indique une réponse spontannée aux questions ouvertes ci-dessus. ***.*** *Continuez en suscitant les réponses des catégories non encore mentionnées par le répondant et cocher dans la colonne Prob, ce qui indique une réponse suscitée. Veuillez marquer d'une croix pour une réponse "non" ou " ne sait pas" lors d'une réponse suscitée. Les cellules grises ne doivent pas être suscitées.*

**Symptômes physiques**	**Spon**	**Prob**		**Spon**	**Prob**
1 Kusokotwa na tumbo<br>Douleurs fortes/ crampes abdominales/ Munda ukukalipa sana			*13* Kunyauka, kukauka ngozi<br>*Peau (déssèchée, plissée)/* ukuma umubili		
2 Kuumwa na tumbo<br>Douleurs abdominales faibles / incomfort/ Munda ukukalipa panono			*14* Kutokuwa na hamu ya kula<br>Manque d'appétit/ kukana lya		
3 Kuumwa na misuli<br>Crampes musculaires/ Amafupa ukukalipa			*15* Maumivu ya kitchwa Maux de tête/ umutwe ubukali		
4 Kutapika *Vomiting* Vômissements/ ukuluka			*16* Kuchafuka roho Nausées/ umulengu lengu		
5 Choo kingi kupita kiasi *Grande quantité de selles/* ukunya amafi ayengi sana			*17* Joto jingi mwilini *Fièvre/* umubili ukukaba		
6 Kuharisha mara kwa mara<br>*Emission fréquente de selles/* ukunyanya sana			*18* Udhaifu Faiblesse/ ukunaka		
7 Choo kama maji ya mchele *water-like stool*<br>Selles acqueuse riziforme/ amafi nga menshi ya laishi			*19* Kudunda roho Palpitations/ umutima ukutunta sana		
8 Choo yenye kamasi Mucus dans les selles/ amafi ya lulenda			*20* Kuchanganyikiwa Confusion des choses / ukupusanya ifintu		
9 Choo yenye damu amaf i ya mulopa *Selles sanguinolantes*			*21* Kupoteza fahamu Inconscience/ ukukanaishiba		
10 Maumivu sehemu ya kunyea<br>*Douleures rectales/ ubukali pa kunya*			*98* Dalili nyingine mwilini<br>Autres symptoms/ ifishibilo fimbi ku mubili		
11 Kiu kali Assoiffé/ikilaka sana			*99* Siwezi kusema Ne sais pas/ shishibe iyoo		
12 Macho kuingia ndani Yeux excavés/ amenso ukufonka					

Veuillez transcrire le récit suscité ______________________________

____

____

____

Si plus d'une catégorie sont cochées dans le tableau ci-dessus, alors continuez de questionner; autrement veuillez entrer le numéro de cathégorie ci-dessous et continuez avec la Q 2.4.

2.3 Je, kati ya hizo dalili ni ipi inaweza sumbua zaidi kuliko zote?
Parmi tous ces symptoms, lequel est celui qui vous incommode le plus?
Kufishibilo ifi fyonse ninshi imicusha sana

Transcrivez le récit:______________________________

____

____

veuillez coder la catégorie la plus incommodante de la liste numérotée des conditions de détresse ci-dessus ☐

2.4 Je, unafikiri [ugonjwa huu] yatamuathiri vipi Musonda/Ngoie kimawazo, kijamii na kiuchumi katika maisha yake ya kila siku?
Comment pensez-vous que cette (maladie) va affecter Musonda/Ngoie sur le plan émotionnel, social et financier dans sa vie quotidienne?
Mwishiba shani ati ubulwele kuti bwa mwikata Musonda/Ngoie mi milangwe mu bwikashi na mu busambashi?

***Veuillez résumer le récit des problèmes du répondant en utilisant ces propres mots:** ________________________________

____

____

En vous basant *sur le récit du répondant veuillez cocher ce qui convient sur la colonne Spon, colonne qui indique une réponse spontannée aux questions ouvertes ci-dessus.* ***.*** *Continuez en suscitant les réponses des catégories non encore mentionnées par le répondant et cocher dans la colonne Prob, ce qui indique une réponse suscitée. Veuillez marquer d'une croix pour une réponse "non" ou " ne sait pas" lors d'une réponse suscitée. Les cellules grises ne doivent pas être suscitées.*

**Impact sociale**	**Spon**	**Prob**		**Spon**	**Prob**
Impact social			Impact émotionnel		
1 Kutengwa na watu wengine<br>Isolement/ ukumutalusha			*6* Huzuni, kukosa raha, wasiwasi<br>Tristesse, anxiété , crainte/ ubukwishi ubulanda no mwenso		
2 Woga wa kuambukiza wengine<br>Crainte d'infecter d'autres/ umwenso wa kwambukisha bambi			Impact financier		
3 Kusitisha huduma za afya<br>Structures de santé saturées/ ku lupitalo takuli incende			*7* Kuongezeka kwa gharama za maisha (kusafiri, vyakula madawa) Coût (transport, nourriture, medicament)/ ukukosa kwa mikalile ifyakulya no muti		
4 Kuathiri shughuli za kila siku<br>Interférence avec le travail /activités quotidiennes/ fyaya nsuka ne ncito			*8* Kupoteza kipato cha familia<br>Perte de revenu du ménage/ ukulufya impiya ku nganda		
5 Kuathiri uhusiano na watu wengine katika jamii<br>Interferences au niveau social/ fyaya nsuka mu mushi			Autres		
			98 Mengine, eleza *:* Autre à préciser/ fimbipo:_____		
			99 Siwezi kusema *Cannot say* Ne sait pas/ shishibe iyoo		

Transcrivez le récit suscité: ________________________________

____

____

Si plus d'une catégorie sont cochées dans le tableau ci-dessus, alors continuez à questionner; autrement veuillez entrer le numéro de catégorie ci-dessous et continuez avec la Q 2.6

2.5 Kati ya matatizo yaliyotajwa hapo juu, ni tatizo lipi haswa linalosumbua zaidi kuliko zote?
Lequel des problèmes que vous avez mentionné ci-dessus, jugez-vous le plus inquiètant?
Bwafya ki pali ifi fyonse bukililepo?

Transcrivez le récit: ________________________________

____

____

____

:Veuillez coder la cathégorie la plus inquiétante de la liste numérotée ci-dessus [ ]

2.6 Unaonaje ubaya wa [ugonjwa huu] kwa Musonda/Ngoie?
Quelle est la gravité de (cette maladie) pour Musonda/Ngoie?
Bwafya ki ku bulwele bwakwa Musonda/Ngoie?

Veuillez cocher uniquement une case:

Mbaya sana<br>Très grave *sana sana 3*	Mbaya kiasi Moyennement grave sana *2*	Sidhani<br>incertain *tafishibikwe 1*	Si mbaya Pas grave *te sana 0*

Transcrivez le récit:____

2.7 Je, Musonda/Ngoie akikosa matibabu ya nje ya [ugonjwa huu] ni nini kitatendeka kwa afya yake?
Quelle est l'issue la plus probable de (cette maladie) pour Musonda/Ngoie sans traitement approprié en dehors du ménage?
Ninshi ingafikila Musonda/Ngoie nga taundepwe ku nganda?

Cocher uniquement une case:

Kifo habituellement mortel / imfwa *4*	Pengine inaweza kuleta kifo Quelque fois mortel / *kuti pambi afwa 3*	Hali mbaya sana, lakini keleta kifo / grave mais non mortel/ *teti afwe 2*	Sina uhakika Incertain/ shishibe iyoo *1*	Kupona vizuri na haraka Récupération rapide/ complete/ *kuti apusuka 0*

Transcrivez le récit:: ____

2.8 Je, wewe au jamii yako yeyote mumewahi pata [ugonjwa huu]?
Avez vous ou quelqu'un d'autre de votre ménage qui a déjà eu cette (maladie) ?
Muli mu nganda umbi uwali nobu bulwele?

Cocher uniquement une case:

Ndio/ *Oui/*<br>e mukwai<br>*3*	Inaweze kana /<br>*Probablement/* limbi<br>*2*	Hakuna hakika /<br>*Incertain* / shishibe<br>*1*	Hapana/ *Non/* iyoo<br>*0*

Transcrivez le récit:____

si "oui" ou "probablement" continuez de questionner, autrement continuer avec la Q 2.10

2.9 Alikuwa nani?
Qui était-ce?
Ali ni nani?

Cochez plusieurs

1 Mimi mwenyewe / *moi-même*/ nebo	*2* Mke/Mume *époux/épouse*/ umulume nangu mukashi	*3* Wazazi *parent* abafyashi	*4* Watoto/ *enfant*/ abana	*5* Dada/kaka/mdogo wangu *frère ou soeur*/ wesu/ kanshi	*6* Watu wengine wanaoishi ndani ya nyumba hii /*autre membre de la famille*/ lupwa	*99* Siwezi kusema *ne sait pas* shishibe

Transcrivez le récit:____

____

____

2.10 Je, kwa kawaida ni nani hupata [ugonjwa huu] mara nyingi? Wanaume au wanawake? Watu wazima au watoto? Matajiri au masikini?
En général quelles sont les personnes les plus susceptibles d'attrapper cette maladie? Ce sont des hommes ou des femmes? Des adultes ou des enfants? Des riches ou des pauvres?
Bantu nshi bakwata ubu bulwele abaume, abanakashi, abana, abakankala abapina?

Questionner les catégories suivantes, si les réponses ne sont pas claires et veuillez ne cocher qu'une réponse pour chacune des trios questions

**Jinsia sexe/ ubwamba**	*1* Wanaume homme / umwaume	*2* Wanawake *femme*/ umwanakashi	*3* Si yeyote ni l'un ni l'autre/ tapali nelyo umo
**Umri âge/ imyaka**	*1* Watu wazima adulte/mukalamba	*2* Watoto enfants/ abana baice	*3* Si wowote ni l'un ni l'autre/ nelyo umo
**Hali ya maisha *classe sociale* / ubwikashi ki**	*1* Matajiri riche/umukankala	*2* Watu masikini pauvre/ abapina	*3* Si yoyote ni l'un ni l'autre/ nelyo umo

Transcrivez le récit:____

____

2.11 Kila mmoja wetu anaweza kueleza mambo yanayotokea kwa njia tofauti tofauti. Je, unafikiria ni nini kimesababisha tatizo la Musonda/Ngoie?
Chacun d'entre nous a sa propre manière d'expliquer ce qui lui arrive. Selon vous qu'est ce qui est à l'origine du problème qui est arrivé à Musonda/Ngoie?
Kila muntu alasosa ifyo amona. Kuli mwebo ninshi yalengele pakuti Musonda/Ngoie alwale?

Transcrivez le récit:____

____

.En vous basant sur le récit du répondant, veuillez cocher ce qui convient sur la colonne Spon, colonne qui indique une réponse spontannée aux questions ouvertes ci-dessous. **.** *Continuez en suscitant les réponses aux catégories non encore mentionnées par le répondant et cocher dans la colonne Prob, ce qui indique une réponse suscitée. Veuillez marquer d'une croix pour une réponse "non" ou " ne sait pas" lors d'une réponse suscitée.*

**Causes apparentes**	**Spon**	**Prob**		**Spon**	**Prob**
Indigestion			*9* Nzi Mouches/ Ni balunshi		
1 Kunywa maji machafu<br>Boire de l'eau contaminée/ kunwa amenshi ya fiko			*10* Malaria malaria/*Paludisme/*<br>maleria		
2 Chakula ambacho hakijahifadhiwa/kimeoza<br>Nourriture non-couverte ou avariée/ kulya ifyakulya ifyabola nangu ifishifimbilwe			*11* Minyoo Vers intestinaux/ imisanda		
3 Chakula kilichokatazwa *food (taboo)* Nourriture interdite (tabou)/ ifyakulya ba kanya			*Magico-religious causes* Causes magico-religieuses		
4 Kula udongo Manger de la terre/ ukulya iloba			*12* Uchawi Sorcellerie/ ubuloshi		
Comportement			*13* Kupenda kwa Mungu La volonté de Dieu/ bufwayo bwa kwa Lesa		
5 Kugusa maji machafu<br>Contact avec de l'eau contaminée/ ukukwata amenshi ya bucafu			*14* Kutofuata mila na desturi Violation d'interdits /coutumes/ kukana sunga itambi		
6 Kutoosha mikono *Not washing hands* /Ne pas se laver les mains/ ukutasamba minwe			Autre		
apo tuleikala Environnement			*98* Mengine, eleza<br>Autre à préciser/ sosa fimbi:______		
7 Mazingira machafu *Dirty environment*<br>Environnement peu salubre/ apo tuleikala ubucafu			*99* Siwezi kusema *Cannot say*<br>Ne sait pas dire/ shishibe		
8 Ukosefu wa choo Manque de toilettes/ ukubulwa ikimbusu					

Transcrivez le récit:____

____

____

si plus d'une catégorie sont cochées dans le tableau ci-dessus, alors continuez à questioner; autrement veuillez entrer le numéro de catégorie ci-dessus et continuez avec la Q 2.13

2.12 Je, kati ya hizo sababu ulizotaja, ni ipi sababu kuu?
Laquelle des causes que vous avez mentionnée considerez-vous comme la cause principale?
Ninshi yalenga pakuti ubu bulwele bwise?

Transcrivez le récit: ____

____

____

veuillez coder la catégorie la plus importante des causes énumérées sur la liste numérotée ci-dessus: ☐

2.13 Je, mtu kama Musonda/Ngoie husaidiwa aje nyumbani kabla ya kutafuta matibabu ya nje?
Que font les gens à domicile pour une personne qui souffre de la maladie de Musonda/Ngoie avant de chercher un traitement ou de l'aide en dehors du ménage?
Abantu bapanga shani ku nganda kuli ubu bulwele bwa kwa Musonda/Ngoie pantanshi yakuya kumbi?

Transcrivez le récit:____

____

____

En vous basant sur le récit du répondant, veuillez cocher ce qui convient sur la colonne Spon, colonne qui indique une réponse spontannée aux questions ouvertes ci-dessous. Continuez en suscitant les réponses des catégories non encore mentionnées par le répondant et cocher dans la colonne Prob, ce qui indique une réponse suscitée. Veuillez marquer d'une croix pour une réponse "non" ou " ne sait pas" lors d'une réponse suscitée. :

**Home-based treatment Traitement à domicile**	**Spon**	**Prob**
1 Kunywa maji mengi au vinywaji vingine Boire beaucoup de liquide / eau/ ukunwa amenshi ayengi nangu fimbi		
2 Dawa za mitishamba (mizizi, magamba, majani) Traitement à base de plantes (racines, écorces, feuilles, etc)/ ukundapwa ne mishila ya panga		
3 Kunywa dawa za vipaketi (ORS) zinazouzwa madukani Traitement de réhydratation par voie oral (SRO)/ ukunwa serum oral		
4 Kuomba dua amasali nangu amapepo *Prayers* Prières		
5 Kutumia dawa za antibiotics za kujinunulia mwenyewe Automédication par antibiotiques / medicaments/ ukunwa imiti we mwine		
6 Hapana kwabula Aucun traitement/ ubundapishi		
7 Kinywaji kilicho na pombe Boissons alcoohlisées/ ukunwa ubwalwa		
98 Mengine, eleza Autre à préciser/ fimbipo:______		
99 Siwezi kusema Ne sais pas/ shishibe iyoo		

Transcrivez le récit:____

____

____

si plus d'une catégorie sont cochées dans le tableau ci-dessus, alors continuez à questioner; autrement veuillez entrer le numéro de catégorie ci-dessous et continuez avec la Q 2.15:

2.14 Je, kati ya matibabu haya, ni tiba ipi inayofaa zaidi kuliko zote?
Laquelle de toutes ces mesures que les gens prennent à la maison vous semble être la plus utile?
Pali ifi fyonse ninshi ili bwino?

Transcrivez le récit: ____

____

veuillez coder la catégorie la plus utile des traitements à domicile énumerées sur la liste numérotée ci-dessus :: ☐

2.15 Kwa kawaida mtu kama Musonda/Ngoie ataenda wapi kupata matibabu nje ya nyumba yake?
Habituellement, où est-ce que Musonda/Ngoie vont chercher le traitement en dehors du ménage?
Nipi Musonda/Ngoie aya fwaya kundapwa kunse ya nganda?

Transcrivez le récit: ______________________________

____

____

En vous basant sur le récit du répondant veuillez cocher ce qui convient sur la colonne Spon, colonne qui indique une réponse spontannée aux questions ouvertes ci-dessous. Continuez en suscitant les réponses des catégories non encore mentionnées par le répondant et cocher dans la colonne Prob, ce qui indique une réponse suscitée. Veuillez marquer d'une croix pour une réponse "non" ou " ne sait pas" lors d'une réponse suscitée.:

**Traitement hors du ménage/ kundapwa kunse ya nganda**	**Spon**	**Prob**
1 Hospitali Structure de santé/ ku lupitalo		
2 Waganga wa kienyeji Tradipraticiens/ kuli bashi nganga		
3 Maduka ya madawa Pharmacie ou pharmacie de rue/ mumashitolo ya miti		
4 Viongozi wa kidini Spiritualistes (pasteur, prêtre, imame, marabout …?) kuli ba shimapepo		
5 Ushauri kutoka kwa ndugu/jamaa na marafiki wanaofanya kazi vituo vya afya Aide prodigué par un parent, ami ou un agent de santé/ ifyo aumfwa ku ba fyashi umunankwe nangu munganga		
98 Sehemu nyinginezo, eleza Autre à préciser / fimbipo:______		
99 Siwezi kueleza Ne sais pas/ shishibe iyoo		

Transcrivez le récit: ______________________________

____

____

si plus d'une catégorie sont cochées dans le tableau ci-dessus, alors continuez à questioner; autrement veuillez entrer le numéro de catégorie ci-dessous et continuez avec la Q 2.17:

2.16 Je, kati ya watu hawa wanaotowa ushauri, ni yupi anayefaa zaidi kuliko wote?
Laquelle des personnes qui peuvent être consultées pensez-vous être la plus utile?
Mu muntu nshi bengamona uwakabaila?

Transcrivez le récit: ______________________________

____

____

veuillez coder la catégorie la plus utile des traitements à l'éxtérieur dans la liste ci-dessus: ☐

2.17 Unafikiria Musonda/Ngoie hafai kuambia watu wengine ila jamii yake kuhusu [ugonjwa huu]?
Pensez-vous que Musonda/Ngoie ne devrait pas divulger cette (maladie) audelà de sa famille proche?
Mulentontokanya ukuti Musonda teti asose ubulwele bwakwe kuli bambi?

Cocher une case seulement

Ndio<br>*Oui*<br>E mukwai *3*	Labda/mchanganyiko<br>*Possible*<br>Limbi *2*	Sidhani<br>I*ncertain*<br>Shishibe *1*	Hapana<br>N*on*<br>Iyoo *0*

Transcrivez le récit:____

____

____

2.18 Watu wengine wangejua kuhusu [ugonjwa] ya Musonda/Ngoie, unafikiria angeaibika au aone haya?
Si les gens savaient, pensez vous que certaines personnes pouvaient faire en sorte que Musonda/Ngoie se sentent honteux ou embarrassé à cause de cette (maladie)?
Abantu bambi nga baumfwa kuti Musonda/Ngoie fyamuletela insoni nangu kutwishika nobu bulwele?

Cocher une case seulement

Ndio<br>*Oui*<br>E mukwai *3*	Labda/mchanganyiko<br>*Possible*<br>Limbi *2*	Sidhani<br>I*ncertain*<br>Shishibe *1*	Hapana<br>N*on*<br>Iyoo *0*

Transcrivez le récit:____

____

____

2.19 Je, watu wengine wakipata kujua kuhusu [ugonjwa huu], unafikiria ingesababisha shida kwa Musonda/Ngoie?
Si d'autres personnes découvraient cette (maladie), pouraient-elles créer des problèmes à Musonda/Ngoie?
Abantu bambi nga baishiba ubu ubulwele kuti bapanga bwafwa kuli Musonda/Ngoie?

Cocher une case seulement

Ndio<br>*Oui*<br>E mukwai *3*	Labda/mchanganyiko<br>*Possible*<br>Limbi *2*	Sidhani<br>I*ncertain*<br>Shishibe *1*	Hapana<br>N*on*<br>Iyoo *0*

Transcrivez le récit:____

____

____

2.20 Je watu wengine wangepata kujua kuhusu [ugonjwa huu], ingesababisha shida kwa familia ya Musonda/Ngoie?
Si d'autrespersonnes découvraient cette (maladie), pourraient-elles créer des problèmes à la famile de Musonda/Ngoie?
Abantu bambi nga baishiba ubu bulwele kuti baleta bwafwa ku lupwa lwakwa Musonda/Ngoie?

Cocher une case seulement

Ndio<br>*Oui*<br>E mukwai *3*	Labda/mchanganyiko<br>*Possible*<br>Limbi *2*	Sidhani<br>I*ncertain*<br>Shishibe *1*	Hapana<br>N*on*<br>Iyoo *0*

Transcrivez le récit:____

2.21 Je, kuna mtu yeyote katika familia hii ambaye angekataa kumpeleka Musonda/Ngoie kupata matibabu kwasababu hawataki wengine wajue kwamba anaugua [ugonjwa huu]?
Y aurait-il quelqu'un dans le ménage qui hésiterait à accomagner Musonda/Ngoie à un centre de traitement parce qu'il ne souhaiterait pas que cette maladie soit connue?
Kuti kwaba lupwa uwingakana ukutwala Musonda/Ngoie ku lupitalo pantu alefwaya ukuti ubu bulwele bwi ishibikwa?

Cocher une case seulement

Ndio<br>*Oui*<br>E mukwai *3*	Labda/mchanganyiko<br>*Possible*<br>Limbi *2*	Sidhani<br>I*ncertain*<br>Shishibe *1*	Hapana<br>N*on*<br>Iyoo *0*

Transcrivez le récit:____

2.22 Je, inawezekana watu wengine nje ya familia wangesaidia, wakijua kwamba Musonda/Ngoie anaugua [maradhi haya]?
Est-il possible que d'autres personnes en dehors de la famille pourraient aider Musonda/Ngoie si elles étaient au courant de cette maladie?
Kuli musango wakuti abashili ba lupwa lwa kwa Musonda bamwafwa nga nabeshiba ubu bulwele?

Cocher une case seulement

Ndio<br>*Oui*<br>E mukwai *3*	Labda/mchanganyiko<br>*Possible*<br>Limbi *2*	Sidhani<br>I*ncertain*<br>Shishibe *1*	Hapana<br>N*on*<br>Iyoo *0*

Transcrivez le récit:____

2.23 Je, nini inawezafanywa kujikinga na [ugonjwa huu]?
Que peut-on faire pour prévenir cette maladie?
Ninshi twingapanga pakuti tuikinge nobu bulwele?

veuillez résumer le récit du répondant sur les options de prévention dans ses propres mots

Transcrivez le récit:____

____

____

En vous basant sur le récit du répondant veuillez cocher ce qui convient sur la colonne Spon, colonne qui indique une réponse spontannée aux questions ouvertes ci-dessous. Continuez en suscitant les réponses des catégories non encore mentionnées par le répondant et cocher dans la colonne Prob, ce qui indique une réponse suscitée. Veuillez marquer d'une croix pour une réponse "non" ou " ne sait pas" lors d'une réponse suscitée.

Prevention kuikingilila	Spon	Prob
1 Kuosha mikono *laver les mains /* kusamba minwe		
2 Maji yaliyochemshwa au yenye dawa eau salubre/ amenshi ayasuma		
3 Chakula safi na salama aliments prepares proprement/ kwipika no busaka ifyakulya		
4 Utupaji na uwekaji wa takataka vizuri elimination correcte des déchets/ ukupwisha ubusali		
5 Uhifadhi wa kinyesi vizuri elimination correcte des selles/ ukusola amafi bwino		
6 Dawa za kinga médicaments à titre préventif/ umuti wa kuikinga		
7 Chanjo vaccins/ indubi		
8 Elimu ya afya education à la santé/ amafundisho pa bumi		
9 Kujikinga kutokana na nguvu za uchawi *protection contre les influences surnaturelles (grisgris)/* ukuikinga ku finkuka		
98 Mengine, eleza *: autres à specifier/* na fimbipo ______		
99 Siwezi kusema/hapana ne sait pas/ shishibe		

Transcrivez le récit:____

____

____

si plus d'une catégorie sont cochées dans le tableau ci-dessus, alors continuez à questioner; autrement veuillez entrer le numéro de catégorie ci-dessous et continuez avec la Q 3:

2.24 Kati ya hizo njia za kujikinga ni ipi inafaa zaidi kuliko zote?
Lequel de ses moyes de prevention vous-semble le plus utile?
Ninshi pali iyi misango ya kuikingilila ili bwino?

Transcrivez le récit: __________________________________

____

____

veuillez coder la mesure de prevention la plus utile de la liste numerotée ci-dessus*:* ☐

3 Vaccins (général et choléra)

3.1 Je, wewe au jamii mumeshawahi kupata chanjo ya aina yoyote (shindano, vidonge au ya maji) hata kama hukuwa unaugua wakati huo?
Avez-vous déjà vous-même ou un membre de votre famille reçu un quelconque type de vaccins (injection, liquide ou pillule) pour vous empêcher de tomber malade, même si vous n'étiez pas malade à ce moment-là ?
Mwalipokapo indubi webo nangu lupwa umulandu wakuikinga kubulwele nangu tamulwele iyo nshita?

Cocher une case seulement

Ndio<br>*Oui*<br>E mukwai *3*	Labda/mchanganyiko<br>*Possible*<br>Limbi *2*	Sidhani<br>*Incertain*<br>Shishibe *1*	Hapana<br>*Non*<br>Iyoo *0*

Transcrivez le récit:____________________

____

____

Si "oui" ou "possible" aller à la question suivante, sinon passez à Q3.3:

3.2 Nani alichanjwa?
Qui l'a reçu?
Nani wapokapo indubi?

Cocher uniquement ce qui convient:

1 Mimi mwenyewe<br>*Moi-même*<br>nebo	*2* Watoto *Children*<br>*Enfants*<br>abana	*3* Watu wazima wanaokaa nyumba hii<br>*Autre adulte du ménage*<br>balupwa abakalamba

Transcrivez le récit:____________________

____

3.3 Kutokana na uzoefu wako, je, unafikiria chanjo husaidia?
Selon votre propre expérience, pensez-vous que les vaccins sont généralement utiles?
Bushe indubi shisuma?

Cocher une case seulement

Ndio<br>*Oui*<br>E mukwai *3*	Wakati mwingine<br>*Quelques fois*<br>Limo *2*	Sidhani<br>*Incertain*<br>Shishibe *1*	Hapana<br>*Non*<br>Iyoo *0*

Transcrivez le récit:____________________

____

____

____

3.4 Je, unafikiria chanjo inaweza kuleta shida yoyote?
Pensez-vous que certains vaccins sont également susceptibles de causer des problèmes?
Indubi shimbi kuti shaleta ubwafya?

Cocher une case seulement

Ndio<br>*Oui*<br>E mukwai *3*	Wakati mwingine<br>*Quelques fois*<br>Limo *2*	Sidhani<br>*Incertain*<br>Shishibe *1*	Hapana<br>*Non*<br>Iyoo *0*

Transcrivez le récit:____________________

____

____

Si "oui" ou "possible" aller à la question suivante, sinon passez à Q3.6:

3.5 Tafadhali nieleze zaidi?
Veuillez svp me parler à ce sujet?
Londololenipo pali uyu mulandu?

Cocher uniquement ce qui convient

**Problems caused by vaccines Les problèmes causés par les vaccins**	**Tick**
1 Kufura/maumivu sehemu iliyochomwa Douleur ou gonflement au point d'injection sindano ubukali no kufimba pa kutungwa	
2 Homa Fièvre Umubili ukukaba	
3 Majipu/kidonda i Abcès:filonda	
4 Kovu Cicatrice umukofu	
5 Mtoto kulia sana bébé qui pleure umwana ukulila sana	
98 Mengine, eleza *:* Autre à préciser:_ na fimbipo _____	
99 Siwezi kueleza Ne sait pas shishibe	

Transcrivez le récit:____

3.6 Ikiwa kuna chanjo ya kumezwa ya kujikinga na kipindupindu bila ya malipo, utakuwa tayari kuitumia?
Si un vaccin buvable gratuit était disponible pour vous protéger du choléra, le prendriez-vous?
Nga bamiletela indubi ya kunwa pa kuikinga na kolera kuti mwasumina*?*

Cocher une case seulement

Ndio<br>*Oui*<br>E mukwai *3*	Labda/mchanganyiko<br>*Possible*<br>Limbi *2*	Sidhani<br>*Incertain*<br>Shishibe *1*	Hapana<br>N*on*<br>Iyoo *0*

Transcrivez le récit:____

Si "oui" ou "possible" aller à la question suivante, sinon passez à Q4:

3.7 Kama chanjo ya kipindupindu ingegharimu 1000 FC bado ungeitumia?
Si le vaccin coûtait 1000 Francs congolais, le prendriez-vous toujours?
Indubi nga ili na 1000 FC kuti mwasumina fye?

Cocher une case seulement

Ndio<br>*Oui*<br>E mukwai *3*	Labda/mchanganyiko<br>*Possible*<br>Limbi *2*	Sidhani<br>*Incertain*<br>Shishibe *1*	Hapana<br>N*on*<br>Iyoo *0*

Transcrivez le récit:____

Si "oui" ou "possible" aller à la question suivante, sinon passez à Q4:

3.8 Kama chanjo ya kipindupindu ingegharimu 5000 FC bado ungeitumia?
Si le vaccin coûtait 5000 Francs congolais, le prendriez-vous toujours?
Indubi nga ili na 5000 FC kuti mwasumina fye?

Cocher une case seulement

Ndio<br>*Oui*<br>E mukwai 3	Labda/mchanganyiko<br>*Possible*<br>Limbi 2	Sidhani<br>I*ncertain*<br>Shishibe 1	Hapana<br>N*on*<br>Iyoo 0

Transcrivez le récit:______________________________

____

Si "oui" ou "possible" aller à la question suivante, sinon passez à Q4:

3.9 Kama chanjo ya kipindupindu ingegharimu 10000 FC bado ungeitumia?
Si le vaccin coûtait 10000 Francs congolais, le prendriez-vous toujours?
Indubi nga ili na 10000 FC kuti mwasumina fye?

Cocher une case seulement

Ndio<br>*Oui*<br>E mukwai 3	Labda/mchanganyiko<br>*Possible*<br>Limbi 2	Sidhani<br>I*ncertain*<br>Shishibe 1	Hapana<br>N*on*<br>Iyoo 0

Transcrivez le récit:______________________________

____

4 Vignette B (enfant)

Tumekuwa tukizungumza juu ya ugonjwa wa Musonda/Ngoie ambaye ni mtu mzima, lakini sasa nitakuambia juu ya hadithi ya ugonjwa wa Kilufya/Chansa ambaye ni mtoto….
Nous venons de parler d'un adulte malade, Musonda/Ngoie, maintenant laissez-moi vous raconter une histoire d'un enfant malade qui s'appelle Kilufya/Chansa ….
Twafuma mukulanda umulandu wa mu kalamba Musonda/Ngoie umulwele nomba nala mishimikila ikishimi kya mwana Kilufya/Chansa umulwele….

4.1 Ugonjwa huu unaitwa aje? (Ni jina gani utatutumia kuelezea mtu mwingine kuhusu ugonjwa huu?)
Quel est le nom de cette maladie? (quel nom utiliseriez-vous pour décrire cette maladie à quelqu'un d'autre?)
Bulweleki lishina nshi mwingapela umbi ulwele ubu bulwele?

veuillez préciser le nom, la terminologie ou un bref descriptif en utilisant les mots tels que utilisés par l'enquêté. Si "autre" veuillez préciser le terme et l'expliquer ici

Transcrivez le récit:______________________________

____

**Types de diarrhées / imisango ya shiki**	
1 Kuharisha kawaida diarrhée normale/ imisango ya shiki	*5* Kipindupindu cholera/ kolera
2 Kuharisha maji maji diarrhée acqueuse/ ukupolomya amenshi	*6* Mchanganyiko mixte/ amalwele ayengi
3 Kuharisha kamasi diarrhée mucoide/ukupolomya ululenda lulenda	*98* Mengineyo, eleza:*autre à spécifier/* ishina limbi
4 Kuharisha damu diarrhée sanguinolante/ ukupolomya umulopa	*99* Siwezi kusema ne sait pas / *shishibe*

veuillez coder un seul nom de la liste numerotée ci-dessus: ☐

.Pour les questions suivantes, veuillez utiliser le nom de la maladie tel que mentionné par la personne en référence à cette maladie/problème, et veuillez utiliser le nom de la personne qui figure dans la vignette.

4.2 Je unaweza kufikiria dalili nyingine ambazo Kilufya/Chansa anaweza kuwa nazo mbali na tulizozitaja hapo juu?
Pouvez-vous penser à d'autres symptomes que Kilufya/Chansa *pourrait presenter en plus de ceux déjà mentionnés?*
Kuti mwaishiba ifishibilo fimbi ifyo Kilufya/Chansa engaba nafyo ifyo tatulandile?

Transcrivez le récit: ____

____

____

En vous basant sur le récit du répondant veuillez cocher ce qui convient sur la colonne Spon, colonne qui indique une réponse spontannée aux questions ouvertes ci-dessous. ***.*** *Continuez en suscitant les réponses des catégories non encore mentionnées par le répondant et cocher dans la colonne Prob, ce qui indique une réponse suscitée. Veuillez marquer d'une croix pour une réponse "non" ou " ne sait pas" lors d'une réponse suscitée. Les cellules grises ne doivent pas être suscitées:*

**symptoms physiques**	**Spon**	**Prob**		**Spon**	**Prob**
1 Kusokotwa na tumbo *douleurs abdominales intenses* / ifumo ubukali sana			*13* Kunyauka, kukauka ngozi peau sèche, flétrie/ umubili ukuma		
2 Kuumwa na tumbo ifumo ubukali mwakubela *douleurs abdominales modérées*			*14* Kutokuwa na hamu ya kula perte d'appetit/ ukutomfwa amu wa kulya		
3 Kuumwa na misuli *crampes musculaires/* imishipa ubukali			*15* Maumiva ya kitchwa maux de tête/ umutwe ubukali		
4 Kutapika vômissements/ ukuluka			*16* Kuchafuka roho *nausées/* umulengu lengu		
5 Choo kingi kupita kiasi selles abondantes / ukunya sana			*17* Joto jingi mwilini *fièvre/* umubili ukukaba		
6 Kuharisha mara kwa mara *passage de selles fréquentes /* ukumfwa amafi lyonse lyonse			*18* Udhaifu faiblesse / ukunaka		
7 Choo kama maji ya mchele selles eau de riz/ amafi nga menshi ya laishi			*19* Kudunda roho palpitations/ umutima ukuntuta		
8 Choo yenye kamasi *muqueuse dans les selles/* amafi ya lulenda lulenda			*20* Kuchanganyikiwa confusion / ukuteshiba bwino ikintu		
9 Choo yenye damu sang dans les selles/ amafi ya mulopa			*21* Kupoteza fahamu perte de conscience / ukulufya kapingu		
10 Maumivu sehemu ya kunyea douleurs anales/ ubukali pa kunya			*98* Dalili nyingine mwilini autres symptomes physiques/ ifishibilo fimbi pa mubili		
11 Kiu kali très assoiffé / ikilaka sana			*99* Siwezi kusema ne sait pas/ shishibe		
12 Macho kuingia ndani yeux excavés/ mu menso ukufonka					

Transcrivez le récit: ____

____

____

si plus d'une catégorie sont cochées dans le tableau ci-dessus, alors continuez à questionner; autrement veuillez entrer le numéro de catégorie ci-dessous et continuez avec la Q 4.4:

4.3 Je, kati ya hizo dalili ni ipi inayosumbua kushinda zote?
Parmi tous ces symptomes, lequel pensez-vous est le plus dérangeant?
Pali ifi fishibilo ninshi icusha sana?

Transcrivez le récit: ____________________

____

____

veuillez coder la catégorie la plus dérangeante de la liste de détresses numérotée ci-dessus ☐

4.4 Je, unafikiria [ugonjwa huu] yatamuathiri vipi Kilufya/Chansa na jamii yake kimawazo, kijamii na kiuchumi katika maisha yake ya kila siku?
Comment pensez-vous que cette maladie va affecter Kilufya/Chansa et les personnes qui les soignent sur le plan émotionnel, social et financier dans leur vie quotidienne?
Mwishiba sha ni ati ubu bulwele kuti bwa sakamika sana Kilufya / Chansa na bantu aba lemundapa mumatontokanyo mu bwikashi ne ndalama?

Transcrivez le récit: ____________________

____

____

.En vous basant sur le récit du répondant veuillez cocher ce qui convient sur la colonne Spon, colonne qui indique une réponse spontannée aux questions ouvertes ci-dessus. ***.*** *Continuez en suscitant les réponses des catégories non encore mentionnées par le répondant et cocher dans la colonne Prob, ce qui indique une réponse suscitée. Veuillez marquer d'une croix pour une réponse "non" ou " ne sait pas" lors d'une réponse suscitée. Les celules grises ne doivent pas être suscitées:*

**impact (enfant ou soignant)**	**Spon**	**Prob**		**Spon**	**Prob**
impact social			*impact émotionel*		
1 Kutengwa na watu wengine / Ukumutalusha			*6* Huzuni, kukosa raha, wasiwasi *y* tristesse, anxiété, craintes / ikumba bulili ubulanda no mwenso		
2 Woga wa kuambukiza wengine *crainte d'infecter d'autres /* Umwenso wakwa mbukisha bambi			*impact financier*		
3 Kusitisha huduma za afya perturbation des services de santé / Ukufulunganya incito ya bundapishi			*7* Kuongezeka kwa gharama za maisha (kusafiri, vyakula, madawa) *coût (transport, nourriture, medicaments) /* umutengo wailindako mu milile no muti		
4 Kuathiri shughuli za kila siku *l interferences avec lels activités quotidiennes / travail /* Ukufulunganya imilimo ya kila bushiku ne ncito			*8* Kupoteza kipato cha familia *perte de revenu de la famille /* ukulufya impiya sha lupwa		
5 Kuathiri uhusiano na watu wengine katika jamii interference dans les relations socials / Ukufulunganya imikale ya bantu			*autres*		
			98 Mengine, eleza *autres à préciser/* na fimbipo ______		
			99 Siwezi kusema , ne sait pas / shishibe		

Transcrivez le récit: ____________________

____

____

4.5 Kati ya shida zilizotajwa hapo juu, ni lipi linalosumbua zaidi kuliko zote?
Lequel des problèmes que vous avez mentionnés ci-dessus, jugez-vous le plus inquiètant?
Bwafya ki pafyo mwalumbula bukililepo?

Transcrivez le récit:________________________________

____

veuillez coder la cathégorie la plus ennuyeuse (une seulke réponse possible) de la liste des impacts numerotés ci-dessus: ☐

4.6 Unaonaje ubaya wa [ugonjwa huu] kwa Kilufya/Chansa?
Quel est la gravité de cette maladie pour Kilufya/Chansa?
Bwafya nshi bwa ubu bulwele kuli Kilufya/Chansa?

Tick one only: cocher une case uniquement

Mbaya sana très grave / *Bwafya sana sana 3*	Mbaya kiasi modérément grave/ bwafyako *2*	Sifikirii incertain shishibe *1*	Si mbaya pas grave/ tali bwafya sana *0*

Transcrivez le récit:________________________________

____

____

4.7 Je, Kilufya/Chansa akikosa matibabu ya nje ya [ugonjwa huu] ni nini kitatendeka kwa afya yake?
Quel est l'issue de cette maladie pour Kilufya/Chansa sans traitement approprié en dehors du ménage?
Ninshi ingafika kuli Kilufya/Chansa nga taundepwe kunse ya nganda?

Cocher une case uniquement

Kifo habituellement mortel / Nimfwa *4*	Wakati mwingine inaweza kusababisha kifo quelque fois mortel / *Kuti afwa 3*	Hali mbaya sana, lakini haisababishi kifo grave mais pas mortel / Bwafya lelo teti afwe *2*	Sifikirii incertain *shishibe 1*	Kupona vizuri na haraka kangu kangu *remission complete/ rapide/* Kuti aumfwa bwino *0*

Transcrivez le récit:________________________________

____

____

4.8 Je, wewe au jamii yako yeyote mumewahi pata [ugonjwa huu]?
Connaissez-vous quelqu'un de votre famille qui a souffert de cette maladie?
Na mwishiba lupwa lwenu uwacushiwe nobu bulwele?

Cocher une case seulement

Ndio<br>*Oui*<br>E mukwai *3*	Labda/mchanganyiko<br>*Possible*<br>Limbi *2*	Sidhani<br>I*ncertain*<br>Shishibe *1*	Hapana<br>N*on*<br>Iyoo *0*

Transcrivez le récit:________________________________

____

____

si "oui" ou "possible", continuez d'enquêter, sinon allez à la Q 4.10:

4.9 Je, alikuwa nani?
C'était qui?
Ni nani?

Cocher tout ce qui convient

1 Mimi mwenyewe moi-même/ Nebo	*2* Mke/Mume époux/ épuse/ Umukashi Umulume	*3* Wazazi parent/ Abafyshi	*4* Watoto enfants	*5* Dada/kaka/mdogo wangu frère ou soeur/ Ba wesu	*6* Watu wengine wanaoishi ndani ya nyumba hii autre membre de la famille/ Balupwa	*99* Siwezi kusema ne sait pas/ shishibe

Transcrivez le récit:____

____

____

4.10 Je, kwa kawaida ni nani hupata [ugonjwa huu] mara nyingi? Wanaume au wanawake? Watu wazima au watoto? Matajiri au masikini?
En général quelles sont les personnes les plus susceptibles d'attrapper cette maladie? Ce sont des hommes ou des femmes? Des adultes ou des enfants? Des riches ou des pauvres?
Ni bani bengakwata ubu bulwele abaume nangu abanakashi abakalamba nangu abaice abakankala nangu abapina?

Questionnez les catégories suivantes, si les réponses ne sont pas claires et veuillez ne cocher qu'une réponse pour chacune des trois questions

**Jinsia / Sexe ubwamba**	*1* Wanaume male/ Umwaume	*2* Wanawake feminine/ Umwanakashi	*3* Si yeyote *ni l'un ni l'autre* /Nelyo umo
**Umri / Age/ Imyaka**	*1* Watu wazima *Adulte* Mukalamba	*2* Watoto enfant / Abana	*3* Si wowote Nelyo umo *ni l'un ni l'autre*
**Hali ya maisha /classe sociale / Ubwikashi**	*1* Matajiri riche/ Mukankala	*2* Watu masikini pauvre/ mupina	*3* Si yoyote *ni l'un ni l'autre* / Nelyo umo

Transcrivez le récit:____

____

____

4.11 Kila mmoja wetu anaweza kueleza mambo yanayotokea kwa njia tofauti tofauti. Je unafikiria nini kimeleta shida la Kilufya/Chansa?
Chacun d'entre nous a sa propre manière d'expliquer ce qui lui arrive. Selon vous qu'est ce qui est à l'origine du problème qui est arrivé à Kilufya/Chansa?
Kila muntu aliba no musango wa kulondolwelamo ifimufikila kuli imwe ni tulo ki ku bucushi bwa Kilufya/Chansa?

Transcrivez le récit:____

____

____

En vous basant sur le récit du répondant veuillez cocher ce qui convient sur la colonne Spon, colonne qui indique une réponse spontannée aux questions ouvertes ci-dessus. ***Continuez en suscitant les réponses des catégories non encore mentionnées par le répondant et cocher dans la colonne Prob, ce qui indique une réponse suscitée. Veuillez marquer d'une croix pour une réponse "non" ou " ne sait pas" pour une réponse suscitée.***

**Perceived causes causes apparentes**	**Spon**	**Prob**		**Spon**	**Prob**
ingestion			*9* Nzi mouches / Ni balunshi		
1 Kunywa maji machafu boire de l'eau contaminée / Kunwa amenshi ya bu cafu			*10* Malaria malaria/paludisme/ Ni maleria		
2 Chakula ambacho hakijahifadhiwa/kimeoza*)* aliments non-protégés/déterioré/ Kulya icakulya ca bola nangu caonaika			*11* Minyoo /vers / Misanda nangu ifishishi		
3 Chakula kilichokatazwa aliments interdits (tabou) / Kulya ifyo tambi sha kanya			*Magico-religious causes causes magico-religieuses*		
4 Kula udongo *manger de la terre/* Kulya iloba			*12* Uchawi *sorcellerie/* Buloshi		
comportement			*13* Kupenda kwa Mungu *volonté de Dieu /* Bufwayo bwa kwa Lesa		
5 Kugusa maji machafu *contact avec de l'eau contaminée /* Kwikata amenshi ya bucafu			*14* Kutofuata mila na desturi *violation de tabou/coutumes* Kukana konka itambi		
6 Kutoosha mikono *Not washing hands ne pas se laver le mains /* Kukana samba ku minwe			*autres*		
environnement			*15* Kunyonyesha *allaitement maternel /* Konsha umwana		
7 Mazingira machafu *environement sâle/* Ni ncende ibi			*16* Kuota meno *"brosser les dents"* Kumenwa ameno		
8 Ukosefu wa choo *manque de latrines/toilettes /* Kubulwa ikimbusu			*98* Mengine, eleza*:_autre à spécifier /* Nafimbipo ______________________________		
			99 Siwezi kusema *ne sait pas /* shishibe		

Transcrivez le récit:____

____

____

si plus d'une catégorie sont cochées dans le tableau ci-dessus, alors continuez à questionner; autrement veuillez entrer le numéro de catégorie ci-dessous et continuez avec la Q 4.13:

4.12 Je, kati ya hizo sababu ulizotaja, ni ipi sababu kuu?
Laquelle des causes que vous avez mentionnées considerez-vous comme la cause principale?
Ni ntulo nshi kufyo mwalumbula ayalenga makamaka?

Transcrivez le récit:____

____

____

:veuillez coder lacatégorie la lus importante (une seule réponse possible) sur la liste des causes apparentes ci-dessus ☐

4.13 Je, mtu kama Kilufya/Chansa husaidiwa aje nyumbani kabla ya kutafuta matibabu ya nje?
Que font les gens à domicile pour une personne qui souffre de la maladie de Kilufya/Chansa avant de chercher un traitement ou de l'aide en dehors du ménage?
Abantu bapanga shani nga bali no bulwele bwa kwa Kilufya/Chansa pantanshi ya kuyaundapwa nangu ukufwaya umusango kunse ya nganda?

Transcrivez le récit:____________________________

____

____

En vous basant sur le récit du répondant veuillez cocher ce qui convient sur la colonne Spon, colonne qui indique une réponse spontannée aux questions ouvertes ci-dessus. Continuez en suscitant les réponses des catégories non encore mentionnées par le répondant et cocher dans la colonne Prob, ce qui indique une réponse suscitée. Veuillez marquer d'une croix pour une réponse "non" ou " ne sait pas" pour une réponse suscitée.

**Home-based treatment traitement à domicile**	**Spon**	**Prob**
1 Kunywa maji mengi au vinywaji vingine boire plus d'eau ou de iquide/ Kunwa amenshi ayengi ne fya kunwa fimbi		
2 Dawa za mitishamba (mizizi, magamba, majani) traitements aux plantes/ Umuti wa panga imishila		
3 Kunywa dawa za vipaketi (ORS) zinazouzwa madukani réhydratation par voie orale Kunwa serum oral		
4 Kuomba dua priers / Amapepo		
5 Kutumia dawa za antibiotics za kujinunulia mwenyewe auto-administration d' antibiotiques/medicaments / Umuti wa maka wa ku lupitalo		
6 Hapana *Nothing* rien / Tapali		
7 Kinywaji kilicho na pombe *boisson alcoohlisée/* Ubwalwa		
98 Mengine, eleza *autre à spécifier* Nafimbipo*:*______		
99 Siwezi kusema ne sait pas / shishibe		

Transcrivez le récit:____________________________

____

____

si plus d'une catégorie sont cochées dans le tableau ci-dessus, alors continuez à questionner; autrement veuillez entrer le numéro de catégorie ci-dessous et continuez avec la Q 4.15:

4.14 Je, kati ya matibabu haya, ni tiba ipi inayofaa zaidi kuliko zote?
Laquelle de toutes ces mesures que les gens prennent à la maison vous semble être la plus utile?
Pali ifi fyonse ninshi ilipo ikankala?

Transcrivez le récit:____________________________

____

____

veuillez coder la catégorie la plus utile les traitements à domicile énumerées sur la liste numérotée ci-dessus : ☐

4.15 Ni wapi Kilufya/Chansa hupelekwa kupata matibabu nje ya nyumba yao?
Habituellement, où est-ce que l'on va amener Kilufya/Chansa pour recevoir un traitement en dehors du ménage?
Ilingi nipi bengatwala Kilufya/Chansa umulandu ondapwe kunse ya nganda?

Transcrivez le récit:____________________

En vous basant sur le récit du répondant veuillez cocher ce qui convient sur la colonne Spon, colonne qui indique une réponse spontannée aux questions ouvertes ci-dessus. Continuez en suscitant les réponses des catégories non encore mentionnées par le répondant et cocher dans la colonne Prob, ce qui indique une réponse suscitée. Veuillez marquer d'une croix pour une réponse "non" ou " ne sait pas" pour une réponse suscitée.

**traitement en dehors du domicile**	**Spon**	**Prob**
1 Hospitali structure de santé/ Kulupitalo		
2 Waganga wa kienyeji tradipraticien/Kuli ba shinganga		
3 Maduka ya madawa pharmacie ou pharmacie de rue/ Ku mashitolo ya miti		
4 Viongozi wa kidini *spiritualistes (pasteur, imam, sheik)* Kuli bashi mapepo		
5 Ushauri kutoka kwa ndugu/jamaa na marafiki wanaofanya kazi vituo vya afya aide prodigué par un parent , ami ou un agent de santé / Ifyo tumfwa ku ba fyashi ku ba nensu nangu munganga		
98 Sehemu nyinginezo, eleza *autre à préciser/* Nafimbipo ______		
99 Siwezi kueleza ne sait pas / shishibe		

Transcrivez le récit:____________________

si plus d'une catégorie sont cochées dans le tableau ci-dessus, alors continuez à questionner; autrement veuillez entrer le numéro de catégorie ci-dessous et continuez avec la Q 4.17:

4.16 Je, kati ya watu hawa wanaotowa ushauri, ni yupi anayefaa zaidi kuliko wote?
Laquelle des personnes qui peuvent être consultées pensez-vous être la plus utile?
Ni nani bengamona uwa cishinka?

Transcrivez le récit:____________________

veuillez coder la catégorie la plus utile des traitements à l'éxtérieur du ménage dans la liste ci-dessus: ☐

4.17 Je, unafikiria mamake au familia ya Kilufya/Chansa hawafai kuambia watu wengine nje ya familia yake kuhusu [ugonjwa huu]?
Pensez-vous que Kilufya/Chansa ne devrait pas divulger cette (maladie) au-delà de sa famille proche?
Mulemona ukuti Kilufya/Chansa teti asose ubu bulwele kubatali balupwa

Cocher une case seulement

Ndio<br>*Oui*<br>E mukwai *3*	Labda/mchanganyiko<br>*Possible*<br>Limbi *2*	Sidhani<br>I*ncertain*<br>Shishibe *1*	Hapana<br>N*on*<br>Iyoo *0*

Transcrivez le récit:____

4.18 Je, watu wengine wangejua kuhusu [ugonjwa] ya Kilufya/Chansa, wangemwaibisha au kumfanya aone haya?
Si les gens étaient au courant de la maladie, pensez vous que certaines personnes pourraient faire en sorte que Kilufya/Chansa se sente honteux ou embarrassé à cause de cette (maladie)?
Abantu bambi nga baishiba ubulwele bwa kwa Kilufya/Chansa kuti fyamutela insoni nangu amasakamika?

Cocher une case seulement

Ndio<br>*Oui*<br>E mukwai *3*	Labda/mchanganyiko<br>*Possible*<br>Limbi *2*	Sidhani<br>I*ncertain*<br>Shishibe *1*	Hapana<br>N*on*<br>Iyoo *0*

Transcrivez le récit:____

4.19 Je, watu wengine wangejua kuhusu [ugonjwa] ya Kilufya/Chansa, ingesababisha shida kwake?
Si d'autres personnes découvraient cette (maladie),pensez-vous qu'elles pourraient créer des problèmes à Kilufya/Chansa?
Abantu bambi nga tabeshibe ubu bulwele kuti ba mulenga imilandu Kilufya/Chansa?
Cocher une case seulement

Ndio<br>*Oui*<br>E mukwai *3*	Labda/mchanganyiko<br>*Possible*<br>Limbi *2*	Sidhani<br>I*ncertain*<br>Shishibe *1*	Hapana<br>N*on*<br>Iyoo *0*

Transcrivez le récit:____

4.20 Je watu wengine wangejua kwamba anaugua [ugonjwa huu], wangeletea shida familia ya Kilufya/Chansa?
Si d'autres personnes découvraient cette (maladie), pensez-vous qu'elles pourraient créer des problèmes à la famile de Kilufya/Chansa?
Abantu bambi nga baishiba ubulwele bwa kwa Kilufya/Chansa kuti baleta ubwafya ku lupwa lwakwe?

Cocher une case seulement

Ndio<br>*Oui*<br>E mukwai *3*	Labda/mchanganyiko<br>*Possible*<br>Limbi *2*	Sidhani<br>I*ncertain*<br>Shishibe *1*	Hapana<br>N*on*<br>Iyoo *0*

Transcrivez le récit:____

4.21 Je, kuna mtu yeyote katika jamii yake ambaye angekataa kumpeleka Kilufya/Chansa kupata matibabu kwa vile hataki wengine wajue kwamba anaugua [ugonjwa huu]?
Y aurait-il quelqu'un dans le ménage qui hésiterait à accomagner Kilufya/Chansa à un traitement parce qu'il ne souaiterait pas que cette (maladie) soit connue?
Kuti kwaba lupwa wa Kilufya/Chansa uwingafwaya ukuti ubu bulwele bwishibikwe?

Cocher une case seulement

Ndio<br>*Oui*<br>E mukwai *3*	Labda/mchanganyiko<br>*Possible*<br>Limbi *2*	Sidhani<br>I*ncertain*<br>Shishibe *1*	Hapana<br>N*on*<br>Iyoo *0*

Transcrivez le récit:____

4.22 Je, inawezekana kwamba watu wengine nje ya familia wangesaidia, wakijua kwamba mtoto huyu ameugua [ugonjwa huu]?
Est-il possible que des personnes de l'exterieur du ménage puissent-être utiles pour l'enfant ou la famille, s'ils étaient au courrant de cette maladie?
Kuti abantu ba kunse ya nganda ba afwilisha umwana nangu ulupwa lwakwe?

Cocher une case seulement

Ndio<br>*Oui*<br>E mukwai *3*	Labda/mchanganyiko<br>*Possible*<br>Limbi *2*	Sidhani<br>I*ncertain*<br>Shishibe *1*	Hapana<br>N*on*<br>Iyoo *0*

Transcrivez le récit:____

4.23 Je, nini inawezafanywa kujikinga na [ugonjwa huu]?
Que peut-on faire pour prévenir cette (maladie)?
Ninshi mwingapanga pakuikinga nobu bulwele?

Transcrivez le récit:____

En vous basant sur le récit du répondant veuillez cocher ce qui convient sur la colonne Spon, colonne qui indique une réponse spontannée aux questions ouvertes ci-dessus. ***.*** *Continuez en suscitant les réponses des catégories non encore mentionnées par le répondant et cocher dans la colonne Prob, ce qui indique une réponse suscitée. Veuillez marquer d'une croix pour une réponse "non" ou " ne sait pas" pour'une réponse suscitée. Les celules grises ne doivent pas être suscitées:*

Prevention	Spon	Prob
1 Kuosha mikono ya (mtoto au wakumchunga mtoto) laver les mains (de l'enfant ou du soignant) Kusamba imnwe ya mwana no ulemundapa		
2 Maji yaliyochemshwa au yenye dawa eau saine Amenshi yasuma		
3 Chakula safi na salama aliments préparé proprement / Ukwipika ifyakulya bwino		
4 Utupaji na uwekaji wa takataka vizuri elimination correcte des déchets / Ukupwisha bwino ubusali		
5 Uhifadhi wa kinyesi vizuri elimination correcte des selles Ukupipa bwino amafi		
6 Dawa za kinga médicaments à titre préventif / Imiti ya kuikinga		
7 Chanjo *Vaccines* vaccins / Indubi		
8 Elimu ya afya éducation à la santé / Amasambililo ba bwikashi		
9 Kujikinga kutokana na nguvu za uchawi (uganga) protection contre les influences supranaturelles (grigris etc) Kuikinga ku fikuko		
98 Mengine, eleza autre à spécifier / Na fimbipo____		
99 Siwezi kusema/hapana ne sait pas / shishibe		

Transcrivez le récit:____

____

____

si plus d'une catégorie sont cochées dans le tableau ci-dessus, alors continuez à questionner; autrement veuillez entrer le numéro de catégorie ci-dessous et continuez avec la Q 5:

4.24 Kati ya hizo njia za kujikinga ni ipi inafaa zaidi kuliko zote?
Lequel de ses moyens de prevention vous-semble le plus utile?
Musango nshi pali iyi yonse wa cishinka ?

Transcrivez le récit:____

____

____

veuillez coder la mesure de prevention la plus utile de la liste numerate ci-dessus ☐

5 Vaccins

5.1 Ikiwa kuna chanjo ya kumezwa ya kujikinga na kipindupindu bila ya malipo, utakuwa tayari kuitumia?
Si un vaccin buvable gratuit était disponible pour protéger votre enfant de cette diarrhée aigue, le prendriez-vous?
Nga bamiletela indubi ya kunwa pa kuikinga umwana wenu na shiki ukalamba kuti mwasumina?

Cocher une case seulement

Ndio<br>*Oui*<br>E mukwai *3*	Labda/mchanganyiko<br>*Possible*<br>Limbi *2*	Sidhani<br>I*ncertain*<br>Shishibe *1*	Hapana<br>N*on*<br>Iyoo *0*

Transcrivez le récit:____

____

Si "oui" ou "possible" aller à la question suivante, sinon passez à Q4:

5.2 Kama chanjo ya kipindupindu ingegharimu 1000 FC ungeitumia?
Si le vaccin coûtait 1000 Francs congolais, le lui donneriez-vous toujours?
Indubi nga ili na 10 FC kuti mwamupela fye?

Cocher une case seulement

Ndio<br>*Oui*<br>E mukwai *3*	Labda/mchanganyiko<br>*Possible*<br>Limbi *2*	Sidhani<br>I*ncertain*<br>Shishibe *1*	Hapana<br>N*on*<br>Iyoo *0*

Transcrivez le récit:

Si "oui" ou "possible" aller à la question suivante, sinon passez à Q4:

5.3 Kama chanjo ya kipindupindu ingegharimu 5000 FC ungeitumia?
Si le vaccin coûtait 5000 FC, le lui donneriez -vous toujours?
Indubi nga ili na 5000 FC kuti mwamupela fye?

Cocher une case seulement

Ndio<br>*Oui*<br>E mukwai *3*	Labda/mchanganyiko<br>*Possible*<br>Limbi *2*	Sidhani<br>I*ncertain*<br>Shishibe *1*	Hapana<br>N*on*<br>Iyoo *0*

Transcrivez le récit:

Si "oui" ou "possible" aller à la question suivante, sinon passez à Q4:

5.4 Kama chanjo ya kipindupindu ingegharimu 10000 FC ungeitumia?
Si le vaccin coûtait 10000 Francs congolais le lui donneriez -vous toujours?
Indubi nga ili na 10000 FC kuti mwamupela fye?

Cocher une case seulement

Ndio<br>*Oui*<br>E mukwai *3*	Labda/mchanganyiko<br>*Possible*<br>Limbi *2*	Sidhani<br>I*ncertain*<br>Shishibe *1*	Hapana<br>N*on*<br>Iyoo *0*

Transcrivez le récit:

6 Remarques finales du répondant

6.1 Sasa tume kuuliza maswali mengi kuhusu haya magonjwa mawili ya kuharisha na jinsi chanjo inaweza kutumika kuzikinga. Ukifikiria hapo awali tulizungumzia shida ya mtu mzima Musonda/Ngoie, na shida ya pili ya mtoto Kilufya/Chansa, kati ya hizo mbili, ni ipi unafikiria chanjo itakuwa na umuhimu zaidi?
Nous venons de vous posez de nombreuses questions concernant ces deux cas de maladies diarrhéiques, nous vous avons également demandé comment vous pensiez qu'un vaccin pouvait être utile pour prévenir ces deux maladies. Si vous repensez au premier problème qui affectait l'adult e Musonda/Ngoie, ainsi qu'au deuxième problème qui affectait l'enfant

Kapambwe/Kabobo, pour quel problème pensez-vous qu'un vaccin pourrait être plus utile?
Twa fuma mukumipusha amepusho ayengi umulandu wa malwele ya kupolomya twakipusha ifyo mulentontokanya umulandu wa ndubi pa kukinga aya malwele. Nga mwabwelela ku bwafya bwa mukalamba Musonda no bwa mwaice Kapambwe ni ku bwafyaki mwamona ukuti indubi kuti ya afwa sana

Cocher une case seulement:

Musonda/Ngoie vignette A (adultes) 3	Kilufya/Chansa vignette B (enfant) 2	Zote muhimu les deux sont importants Kuli bonse babili *1*	Hakuna ile muhimu aucun n'est important Tapali nelyo kimo 0	Sijui/siwezi sema ne sait pas shishibe 99

Transcrivez le récit:______________________________________

____

6.2 Je, kuna mambo mengine unaweza kuniambia kuhusu shida za kiafya ambazo tumesema ama uzoefu wako na chanjo? Nitashukuru kama una mawaidha mengine yakuongezea.
Pouvez-vous me dire autre chose concernant le problème de santé que nous avons discuté ou concernant votre expérience avec la vaccination? Tout autre commentaire, avis ou suggestion sont les biens venus.
Kuti mwangebako fimbi umulandu wa bwafya bwa bumi ubo twafuma mukwambaulapo nangu amatontokanyo yenu mu bwikashi umulandu wa ndubi? Fyonse pali uyu mulandu kuti fyaba ifisuma:

Transcrivez le récit:______________________________________

____

<table>
<tr><td colspan="4">équipe (entourer ce qui convient)</td><td>A B ;C D</td></tr>
<tr><td colspan="4">heure de fin d'interview (heure: minutes)</td><td></td></tr>
<tr><td colspan="4">enquêteur (nom/ signature)</td><td></td></tr>
<tr><td colspan="4">données enregistrées par (nom/signature)</td><td rowspan="3"></td></tr>
<tr><td>enregistré?</td><td>oui</td><td>non</td><td>partiel</td></tr>
<tr><td>Nom du fichier sur enregistrateur</td><td colspan="3"></td></tr>
<tr><td colspan="4">controllé par (date/initiales)</td><td></td></tr>
<tr><td colspan="4">traductions et saisie des récits transcripts (date)</td><td></td></tr>
<tr><td colspan="4">1ère entrée de données (dates/initiales)</td><td></td></tr>
<tr><td colspan="4">2ème entrée de données (dates/initiales)</td><td></td></tr>
</table>

Commentaires additionnels de l'équipe d'enquêteurs

Notes par rapport à l'intérêt des répondants et sur la qualité de l'interview, ainsi que d'autres détails et informations d'intérêt de cet inteview

____
