# Supplementary material for: Comparing sociocultural features of cholera in three endemic African settings
Source: BMC Med. 2013 Sep 18;11:206. doi: 10.1186/1741-7015-11-206 (PMC4016292; doi:10.1186/1741-7015-11-206)
Supplement: Additional file 2 — EMIC interview for study of community views of cholera in western Kenya. [file 1741-7015-11-206-S2.pdf]

Diarrhoeal disease vaccination study: Explanatory Model Interview Catalogue (EMIC)

Maseno University

in collaboration with
World Health Organization and Swiss Tropical & Public Health Institute

Dholuo/English Version of 11/03/2010 (Final)

**Date interview (dd-mm-yyyy)**		**EMIC ID (KX-###)** *X: U=Kisumu, R=Siaya*	K __-___ ___ ___
**Start time of interview (hh:mm)**			

General information

Tick appropriate: *Tick one only:*

**Sex**	*1* F	*2* M	**Approximate age (years)**		**Site**	U Kisumu	R Siaya

Introduction

"Erokamano kuom miya thuolo mar wuoyo kodi. Abiro penji weche molure kod ngima ma e gweng ka. Gi mabropenji olure gi weche ma samoro ing'eyo to samoro ok ing'eyo. Kata gin gi ma samoro iseneno kata pok ineno, to maduong' en ni imiya pachi kalure kod weche ma wadwa wuoye gi. Duoko ma imiyowa kachiel gi pachi biro konyowa e konyo jo mamoko mani gi chandruok machalo kod mawadwa wuoye gi. Ka wachako, to adwa penji penjo ma olure kodi."
Thank you for letting me speak with you today. I will be asking you questions about health problems that could affect people in your community. You may recognise these conditions, or they may be unfamiliar. In either case I would like to understand your ideas about it. Your answers and thoughts will help us to assist people who have these problems. But first, a few questions about your background.

1 Socio-economic and demographic information

1.1 Ise kendo/ose kendi *Marital status*

Tick one only:

1 Pok akendo/okenda nyakanene *Never married*	*2* Osekenda/ asekendo *Married*	*3* Sani ok wadak kanyakla *Separated*	*4* Wawere *Divorced*	*5* Wadak kanyakla to pok wakendre *Living together*	*6* Jaoda no nindo/an chi liel *Widowed*	*7* Ok anyal wacho Cannot say

1.2 To kwan jo ma udakgo e ot achiel *Household size*

Kwan joma udakgo e ot achiel *Number of people living in the household* ☐

1.3 Kwan nyithindo ma udak go e ot achiel *Children living in the household*

Enter 0 when answer is none for a given category:

Kwan nyithindo *Number of children*	Matindo ne higni abich *< 5 yrs*	Ma e kind higni abich gi apar *5-10 yrs*	Ma e kind higni a par to gi a par gi abich abiryo *10-17 yrs*
Yawuoyi *Boys*			
Nyiri *Girls*			

Copy for ☐ **quantitative** data / ☐ **qualitative** data (narratives)

1.4 Wat manie kindi gi wuon ot ma idake *Relationship with household head*

Tick one only: *Tick sex of household head:*

1 An *Self*	*2* En jaoda *Spouse*	*3* En janyuol na *Parent*	*4* En Nyathiwa *Sibling*	*5* En Nyathina *Offspring*	*6* Mamoko *Other Specify:______*

Sex	*1* F	*2* M

1.5 Itiyo tich mane/ Yori mar yuto en mane *Main occupational status*

Tick one only:

1 Pur *Agriculture*	*2* A jamak rech *Fishing*	*3* Andikora *Self-employment (not 1&2)*	*4* Ondika msara *Formally employed*	*5* An chi ot *Housewife*
6 Aja tich ot *Housemaid*	*7* A jakibarua/jua kali/ajatij luedo *Casual labourer*	*8* A ja skul *Student*	*9* Nase ritaya *Not active/retired*	*10* Mamoko, ler *Other specify:______*

1.6 Sombi *Education*

Tick highest level of educational experience:

1 Ok a somo *No education*	*2* A gik e primari *Primary school*	*3* An ja sekondari *Secondary school*	*4* Atimo kos *Vocational school*	*5* Kolej *College*	*6* Mbalariany University

1.7 Isomo kuom higni adi *Years of education*

☐ Ok anyal wacho *Cannot say* ☐

1.8 Ilemo gi jomage *Religion*

Tick one only:

1 Jamuslam *Muslim*	*2* Jakristo *Christian*	*3* Mamoko, ler *Other specify:______*	*4* Ok odwa wacho *Undisclosed*

Denomination / other detail: ______________________________

1.9 In ja piny mane *Nationality*

Tick one only:

1 Jakenya *Kenyan*	*2* An jaoko, an ja, ler *Other specify:______*

1.10 “E odu, bende nitie yuto ma ogurore (kendo moromo)?” *Is your household income usually reliable (and dependable)?*

Tick one only, not disclosed means uncertain:

Eeh/kamano *Yes 3*	Dibedi *Possibly 2*	Ok ong’ere *Uncertain 1*	Ooyo *No 0*

Narrative:______________________________

____

____

____

1.11 “Yo maduong’ mar yuto e ot ma idake en mane?” *What main sources of income are there in your household?*

Narrative:______________________________

____

____

____

Tick all that apply:

Sources of income	Own	Others
1 Tich michula msara *Employment for cash*		
2 Tich michule eyoremamoko maok gi pesa *Employment in kind*		
3 Atiyo tija maok mar pur *Non-farm self-employment*		
4 Aloko gi puodho *Selling agricultural produce*		
5 Aloko gi nam (rech/omena) *Selling fish and seafood*		
6 Ichula kodi *Rent*		
7 Ikopona *(money sent from outside the household)*		
8 Aja penson *Pension*		
98 Mamoko, ler *Other specify:______* /______		
99 Ok anyal wacho *Cannot say*		

1.12 "E dwe mokadho, niloso pesa adi in iwuon? To jaodi ka chiel gi jomamoko ma udak go?"
How much money did you make during the last month on your own? And what about your spouse and other household members?

Narrative:

____

____

____

Query for items not mentioned, clarify if needed. If there is no income, enter 0 in KSh column. If respondent is widowed, then tick "cannot say" for category 2:

Monthly income	KSh		
1 An a wuon<br>*Own*		Ok anyal wacho<br>*Cannot say*	
2 Jaoda<br>*Living spouse*		Ok anyal wacho<br>*Cannot say/widowed*	
3 Joma moko ma adak go<br>*Additional household income*		Ok anyal wacho<br>*Cannot say*	

Introduction to vignettes

"Amor gi bedo ni ikawo thuolo mondo mi walose e wi weche mag ngima ma e gweng'u ka. Adwa ng'eyo pachi koum weche gi. Daher mondo wang'e pachi, omiyo bed thuolo mondo ipim gi ma iparo kuom weche gi. Adwa goni mbekni ariyo ewi joma ni gi chandruokge mopogore."
I appreciate your willingness to talk to me about a few health problems that affect people in your community. I want to understand how you think about them. It is your ideas that I am interested in, so please don't feel shy to tell me your personal opinion. I will tell you two stories about persons who are having two different problems.

2 Vignette C

"We apimni mbaka Otieni/Atieno manenigi chandruok mar ngima..."
Let me tell you the story about Otieno/Atieno who recently had a health problem …

2.1 "Tuo ni iluongo nang'o? (Nying mane mar tuoni ma di pim ne go ng'at machielo?)"
What is the name of this disease? (By what name would you describe the condition to someone else?)

Specify name, summary term or short description in his/her own words. If 'other', specify term and explain here:

***Narrative**:____

____

____

____

**Types of diarrhoea**	
1 Mano mana diep *Normal diarrhoea*	*5* Kipindupindu/Nyaldiema *Cholera*
2 Karenda renda *Watery diarrhoea*	*6* Tuoche moriwre *Multiple*
3 Orianyancha *Mucous diarrhoea*	*98* Mamoko, ler *Other, specify:______*
4 Odiewo remo *Bloody diarrhoea*	*99* Ok anyal wacho *Cannot say*

Code the name from the above numbered list: ☐

Use the name as identified for this disease instead of referring to disease/problem and use the name of the person mentioned in the vignette in the following questions.

2.2 "Bende inyalo ny'isa ranyisi mamoko ma Otieno/Atieno nyalo bedo go ka waweyo ma wase wuoeyegi?"
Can you think of any other symptoms that this Otieno/Atieno is likely to experience besides the ones we already mentioned?

Summarize the respondent's account of problem in his/her own words:

***Spontaneous narrative**: ____

____

____

____

Based on the respondent's account tick problems which are mentioned under the Spon *column indicating a* spontaneous *response to the open-ended question above. Continue by probing for any categories not yet mentioned and tick them in the* Prob *column, indicating a* probed *response to screening. Make a cross when "no" or "cannot say" was the reply to probed categories. Shaded cells must not be probed.*

**Physical symptoms**	**Spon**	**Prob**		**Spon**	**Prob**
1 Ich maremo kabisa *Severe pain/abdominal cramps*			*13* Pien joure kendo tuo *Skin (loose, dry, shrivelled)*		
2 Ich kach e piny ich *Low-level abdominalpain/discomfort*			*14* Dhok marach *Loss of appetite*		
3 Ich ma muorore *Muscle cramps*			*15* Wich bar *Headache*		
4 Ng'ok *Vomiting*			*16* Chuny malepo *Nausea*		
5 Dhi oko ma ng'eny *Large amounts of stool*			*17* Del machwakre *Fever*		
6 Dhi oko kinde ka kinde *Frequent passing of stool*			*18* Jony *Weakness*		
7 Diep machalo mchele/marochere *Rice water-like stool*			*19* Adundo gore matek *Palpitations*		
8 Diep motimo othinyo *Mucus in stool*			*20* Paro ok chung' kare *Confusion*		
9 Diep motimo remo *Bloody stool*			*21* Paro lal *Unconsciousness*		
10 Rem e sianda *Rectal pain*			*98* Ranyisi mamoko *Other physical symptoms*		
11 Riyo mang'eny *Very thirsty*			*99* Ok anyal wacho *Cannot say*		
12 Wang' modonjo iye *Sunken eyes*					

***Probed narrative**: ____

____

____

____

If more than one category is coded in the above table, enquire further; otherwise enter the single coded category number below, and proceed with Q 2.4:

2.3 "Kuom ranyisi gi duto, ere ma ineno ka rach moloyo?"
Among all these symptoms which one do you consider the single most troubling?

Narrative: ____

____

____

____

Code the most troubling category from the above numbered list of patterns of distress: ☐

2.4 "Iparo ni [tuoni] nyalo yungo Otieno/Atieno nade eparo, e tudruok gi jo mamoko, kata eyore mar pesa kata e dakne ma pile?"
How do you think that this [disease] will affect Otieno/Atieno emotionally, socially and financially in his/her daily life?

Summarize the respondent's account of problem in his/her own words:

***Spontaneous narrative**: ______________________________

____

____

____

Based on the respondent's account tick problems which are mentioned under the Spon *column indicating a* spontaneous *response to the open-ended questions above. Continue by probing for any categories not yet mentioned and tick them in the* Prob *column, indicating a* probed *response to screening. Make a cross when "no" or "cannot say" was the reply to probed categories.*

**Impact**	**Spon**	**Prob**		**Spon**	**Prob**
Social impact			*Emotional impact*		
1 Riuruok gi jomoko *Isolation from others*			*6* Ok obed ng'ama mor, obedo gi buok ka chiel gi paro *Sadness, anxiety, worry*		
2 Luoro mar lando tuo ne jomamoko *Fear of infecting others*			*Financial impact*		
3 Kelo pek e kuonde mag thieth *Disruption of health services*			*7* Tiyo gi pese (e wuoth, chiemo to gi yath) *Costs (transportation, foods, drugs)*		
4 Nyalo mone timo tije mapile *Interference with work/daily activities*			*8* Yuto mar jo ot dok piny *Loss of family income*		
5 Nyalo miye luoro mar tudruok gi jowadgi *Interference with social relationships*			*Miscellaneous*		
			98 Mamoko, ler *Other, specify:*______		
			99 Ok anyal wacho *Cannot say*		

***Probed narrative**: ______________________________

____

____

____

If more than one category is coded in the above table, enquire further; otherwise enter the single coded category number below, and proceed with Q 2.6:

2.5 "Kuom chandruok ma ichiwo gi ere malich moloyo gitee?"
Which of these problems that you have mentioned do you consider the single most troubling?

Narrative: ______________________________

____

____

____

Code the most troubling category from the above numbered list of impacts: ☐

2.6 "[Tuoni] lich maromo nade ne Otieno/Atieno?"
How serious is this [disease] for Otieno/Atieno?

Tick one only:

Lich kabisa *Very serious 3*	Olich moromo *Moderately serious 2*	*Ok ang'eyo Uncertain 1*	Okolich *Not serious 0*

Narrative: ______________________________

____

____

____

2.7 “Ang’o manyalo timre ne Otieno/Atieno kapo ni [tuoni] ok othiedhi?”
What is the most likely health outcome of this [disease] for Otieno/Atieno without appropriate treatment from outside?

Tick one only:

Tho<br>*Usually fatal 4*	Samoro tuo ni nyalo nege/tuoni ja negogaji<br>*Sometimes fatal 3*	Tuoni lich to ok oneki<br>*Serious but not fatal 2*	Ok ong’ere<br>*Uncertain 1*	Ochango ga kendo piyo<br>*Full/quick recovery 0*

Narrative:____

2.8 “Bende in, kata ng’at ma udakgo osega bedie gi [tuoni]?”
Have you or somebody else in your household ever had this [disease]?

Tick one only:

Eeh *Yes 3*	Nyalo betie *Possibly 2*	Ok an gi adier *Uncertain 1*	Ooyo *No 0*

Narrative:____

If* yes *or* possibly*, enquire further otherwise go to Q 2.10:

2.9 “Mano ne en ng’a?”
Who was that?

Tick all that apply:

1 An *Self*	*2* Jaoda<br>*Spouse*	*3* Janyuolna<br>*Parent*	*4* Nyithindo<br>*Children*	*5* Nyithindwa<br>Sibling	*6* Jo mamoko ma wadakgo<br>*Other household member*	*7* Ok anyal wacho<br>*Cannot say*

Narrative: ____

2.10 “Ka ing’iyo, to joma nade ma hinyoga bedo gi tuo ni? Mon koso chwo? Jomadongo koso nyithindo? Jomomewo koso jo chan?”
In general, who is most likely to get this [disease]? Is it males or females? Adults or children? Rich or poor people?

Enquire about the following categories if not clear from response and tick one response only for each of the three following queries:

**Chuech *Sex***	*1* Chwo *Male*	*2* Mon *Female*	*3* Ng’ato a ng’ata *Neither*
**Higni *Age***	*1* Jomadongo *Adults*	*2* Nyithindo *Children*	*3* Ng’ato a ng’ata *Neither*
**Okang’ mar yuto *Social class***	*1* Joma omeo *Rich*	*2* Joma odhier *Poor*	*3* Ng’ato a ng’ata *Neither*

Narrative:____

2.11 “Ji neno ga gik moko e yore mopogre o opogre. Iparo ni ang’o ma okelo ne Otieno/Atieno tuo ni?”
Each of us may explain something that happens in various ways. What do you think has caused Otieno/Atieno’s problem?

Summarize the respondent’s ideas about causes in his/her own words:

***Spontaneous narrative**: ______________________________

____

____

____

Based on the respondent’s account tick perceived causes in the Spon *column indicating a* spontaneous *response to the open-ended question above. Continue by probing for any category not yet mentioned and tick them in the* Prob *column, indicating a* probed *response to screening. Make a cross when “no” or “cannot say” was the reply to probed categories.*

**Perceived causes**	**Spon**	**Prob**		**Spon**	**Prob**
Ingestion			*9* Lwang’ni *Flies*		
1 Modho pi ma ok ler *Drinking contaminated water*			*10* Malaria		
2 Chamo chiemo ma owichore kata okler *Unprotected/spoiled food (biological)*			*11* Njofni *Worms*		
3 Ochamo chiemo mar kwero *Forbidden food (taboo)*			*Magico-religious causes*		
4 Chamo loo *Eating Soil*			*12* Juok *Witchcraft*		
Behaviour			*13* Dwach Nyasaye *God’s will*		
5 Tiyo gi pi maok ler *Contact with contaminated water*			*14* Koso luo chik *Violation of taboo/tradition*		
6 Ok olog ga *Not washing hands*			*Miscellaneous*		
Environment			*98* Mamoko, ler *Other, specify:______*		
7 Aluora molil *Dirty environment*			*99* Ok anyal wacho *Cannot say*		
8 Onge choo *Lack of latrines/toilets*					

***Probed narrative**: ______________________________

____

____

____

If more than one category is coded in the above table, enquire further; otherwise enter the single coded category number below, and proceed with to Q 2.13:

2.12 “Kuom yore ma isewachonagi, ere ma ineno ka en yoo maduong manyalo kelo tuoni?”
Which one of these causes that you have mentioned do you consider the main cause?

Narrative: ______________________________

____

____

____

Code the most important category from the above numbered list of perceived causes: ☐

2.13 “Ang’o ma ji timo ne ga ji manigi tuo kamar Otieno/Atieno ei dala kapok gidhi yudo thieth kata kony oko mar dala?”
What do people do at home for someone like Otieno/Atieno with this [disease] before looking for treatment or help outside their homes?

Summarize the respondent’s account of home-based treatment in his/her own words:

***Spontaneous narrative**: ______________________________

____

____

____

Based on the respondent's account tick home-based treatment categories in the Spon *column indicating a* spontaneous *response to the open-ended question above. Continue by probing for any home-based treatment categories not yet mentioned and tick them in the* Prob *column, indicating a* probed *response to screening. Make a cross when "no" or "cannot say" was the reply to probed categories.*

**Home-based treatment**	**Spon**	**Prob**
1 Omodho pi kata gik mimadho mang'eny *Drinking more water or liquids*		
2 Omadho yadh nyaluo *Herbal treatment (roots, bark, leaves)*		
3 Omadho yedhe mag duka ma medo pi kaka ORS *Oral rehydration therapy*		
4 Lamo *Prayers*		
5 Ngato ngiewo yath e duka kata famasi to muonyo *Self-administered antibiotics/drugs*		
6 Onge *Nothing*		
7 Madho kong'o kata chang'aa *Drink containing alcohol*		
98 Mamoko, ler *Other, specify:______*		
99 Ok anyal wacho *Cannot say*		

***Probed narrative**: ____________________

____

____

____

If more than one category is coded in the above table, enquire further; otherwise enter the single coded category number below, and proceed with Q 2.15:

2.14 "Kuom thieth ma ji chiwo ei dala, mane ma iparo ni nigi kony moloyo?"
Which one of all these things people do at home do you think is likely to be most helpful?

Narrative: ____________________

____

____

____

Code the most helpful category from the above numbered list of home-based treatments: ☐

2.15 "Ji kaka Otieno/Atieno nyalo ga yudo thieth kanye kopogore gi dala?"
Where will someone like Otieno/Atieno usually go for treatment outside his/her home?

Summarize the respondent's account of outside treatment in his/her own words:

***Spontaneous narrative**: ____________________

____

____

____

Based on the respondent's account tick outside treatment categories in the Spon *column indicating a* spontaneous *response to the open-ended question above. Continue by probing for any outside treatment categories not yet mentioned and tick them in the* Prob *column, indicating a* probed *response to screening. Make a cross when "no" or "cannot say" was the reply to probed categories.*

**Outside treatment**	**Spon**	**Prob**
1 Osiptal *Health facilities*		
2 Jo thieth nyaluo *Traditional healers*		
3 Famisi kata kuonde mi use yath *Pharmacy or over-the-counter drugs*		
4 Jolemo *Faith healers (Pastors, Imams, Sheikhs)*		
5 Yudo thieth kuom nyamreche, wede kata osiepe *Informal help from health-worker, friend/relative*		
98 Kuonde ma moko, ler *Other, specify:______*		
99 Ok anyal wacho *Cannot say*		

***Probed narrative**: ____________________

____

____

____

If more than one category is coded in the above table, enquire further; otherwise enter the single coded category number below, and proceed with Q 2.17:

2.16 “Kuom jogi duto, ere ngato manyalo chiwo kony kabisa mohingo mokogo?”
Which one of these people they might consult do you think is most helpful?

Narrative: ____

____

____

____

Code the most helpful category from the above numbered list of outside treatments: [ ]

2.17 “Iparo ni Otieno/Atieno ok onego opimne jokmamoko maok jo odi wach [tuoni] kose?”
Do you think Otieno/Atieno should not disclose this [disease] beyond his/her closest family?

Tick one only:

Eeh *Yes 3*	Nyalore/jomoko *Possible/mixed 2*	Ok ang’eyo *Uncertain 1*	Ooyo/ahah *No 0*

Narrative: ____

____

____

2.18 “Ka jomoko ong’eyo, iparo ni ginyalo miyo Otieno/Atieno obed gi wich kuot kuom bedo gi [tuoni] kose?”
If they knew, do you think some people might make Otieno/Atieno feel ashamed or embarrassed because of this [disease]?

Tick one only:

Eeh *Yes 3*	Nyalore/jomoko *Possible/mixed 2*	Ok ang’eyo *Uncertain 1*	Ooyo *No 0*

Narrative: ____

____

____

2.19 “Kaponi jomamoko ofwenyo wach [tuoni], iparoni ma nyalo miyo Otieno/Atieno chandruok?”
Would others finding out about this [disease] cause problems for Otieno/Atieno?

Tick one only:

Eeh *Yes 3*	Nyalore/jomoko *Possible/mixed 2*	Ok ang’eyo *Uncertain 1*	Ooyo *No 0*

Narrative: ____

____

____

2.20 “Kaponi jomamoko ofwenyo wach [tuoni], iparo ni mano nyalo kelo chandruok ne joodgi Otieno/Atieno?”
Would others finding out about this [disease] cause problems for the family of Otieno/Atieno?

Tick one only:

Eeh *Yes 3*	Nyalore/jomoko *Possible/mixed 2*	Ok ang’eyo *Uncertain 1*	Ooyo *No 0*

Narrative: ____

____

____

2.21 “Bende nyalo bedo ni nitiere ngato ei od gi Otieno/Atieno manyalo neno pek mar tere e thieth nikech ok gi dwa ni wach [tuoni] olandre?”
Might there be someone in the household who would hesitate to bring Otieno/Atieno to treatment because they did not want the [disease] to be known?

Tick one only:

Eeh *Yes 3*	Nyalore/jomoko *Possible/mixed 2*	Ok ang’eyo *Uncertain 1*	Ooyo *No 0*

Narrative:____

____

____

2.22 "Bende iparo ni joma moko nyalo chiwo kony ne Otieno/Atieno ka ging'eyo wach [tuoni]?"
Is it likely that others outside the family finding out about this [disease] would be helpful to Otieno/Atieno?"

Tick one only:

Eeh *Yes 0*	Nyalore/jomoko *Possible/mixed 1*	Ok ang'eyo *Uncertain 2*	Ooyo *No 3*

Narrative:____

____

____

2.23 "Ang'o ma inyalo tim mondo ogeng' [tuoni]?"
What can be done to prevent this [disease]?

Summarize the respondent's account of prevention options in his/her own words:

***Spontaneous narrative**: ____

____

____

____

Based on the respondent's account tick prevention categories in the Spon *column indicating a* spontaneous *response to the open-ended question above. Continue by probing for any prevention categories not yet mentioned and tick them in the* Prob *column, indicating a* probed *response to screening. Make a cross when "no" or "cannot say" was the reply to probed categories.*

**Prevention**	**Spon**	**Prob**
1 Luoko lwedo *Wash hands*		
2 Modho pi mochwaki kata moket yath *Safe water*		
3 Chamo chiemo maler *Clean/safe food*		
4 Wito yugi e yo ma kare *Safe disposal of garbage*		
5 Losruok eyo makare *Safe disposal of stool*		
6 Muony yien mageng'o tuoni *Preventive drugs*		
7 Chanjo *Vaccines*		
8 Puonjruok e weche molure gi ngima *Health education*		
9 Thieth gi yien nyaluo *Protection from supernatural influence (charms etc)*		
98 Mamoko, ler *Other, specify:______*		
99 Ok anyal wacho/onge *Cannot say/Nothing*		

***Probed narrative**: ____

____

____

____

If more than one category is coded in the above table, enquire further; otherwise enter the single coded category number below, and proceed with Q 3:

2.24 "Kuom yore gi duto, ere yo miparo ni ber kabisa e geng'o [tuoni]?"
Which one of these ways of prevention do you think is most useful?

Narrative: ____

____

____

____

Code the most useful way from the above numbered list of preventive measures: [ ]

3 Vaccines [General and C]

3.1 "Bende in, kata ng'ato ang'ata e odu osega yudo chanjo (sindan, amadha, amuonya) moro a mora mar geng'o tuo kata ka ne okituo?"
Have you or anyone in your household received any type of vaccine, that is, something you were given (injection, liquid or pill) to prevent you from getting sick, even though you were not sick at the time?

Tick one only:

Eeh *Yes 3*	Nyalore *Possibly 2*	Ok ang'eyo *Uncertain 1*	Ooyo *No 0*

Narrative:____

____

____

____

If "yes" or "possibly", enquire further, otherwise go to Q 3.3:

3.2 "Mano ng'amanochanji?"
Who got it?

Tick all that apply:

1 An *Self*	2 Nyithindo *Children*	3 Joma dongo e ot *Other adult(s) in household*

Narrative: ____

____

____

____

3.3 "Kalure gi ng'eyoni, bende iparo ni chanjo ber?"
Based on your experience, do you think vaccines are generally helpful?

Tick one only:

Eeh *Yes 3*	Samoro *Sometimes 2*	Ok ang'eyo *Uncertain 1*	Ooyo *No 0*

Narrative:____

____

____

____

3.4 "Bende iparo ni nitiere chanjo moko manyalo kelo chandruok?"
Do you think some vaccines are also likely to cause problems?

Tick one only:

Eeh *3*	Nyalore *Possibly 2*	Ok ang'eyo *Uncertain 1*	Ooyo *No 0*

Narrative:____

____

____

____

If "yes" or "possibly", enquire further, otherwise go to Q 3.6:

3.5 “Pimna kaka ma nyalore.“
Please tell me about that.

Tick all that apply:

Problems caused by vaccines	Tick
1 Rem kata kuot mar kama nochuo *Pain/swelling at injection site*	
2 Del machwakore *Fever*	
3 Adhola *Infection/abscess*	
4 Mbala *Scar*	
5 Nyathi nyalo ywagre *Crying baby*	
98 Mamoko, ler *Other, specify:______*	
99 Ok onyal wacho *Cannot say*	

Narrative: ____

____

____

____

3.6 “Kaponi ni chanjo mar kipindupindu/nyaldiema ma imadho oyudre, bende di madhe ka ichiwe nono?”
If a vaccine that you swallow becomes available to prevent cholera, would you take it if it was made available without charge?

Tick one only:

Eeh *Yes 3*	Nyalore *Possibly 2*	Ok ang’eyo *Uncertain 1*	Ooyo *No 0*

***Narrative**: ____

____

____

____

If “yes” or “possibly”, enquire further, otherwise go to Q 4:

3.7 “To kapo ni chanjo ni ichiwo gi siling 65 bende di ti kode?”
If the vaccine were to cost 65 Ksh would you still take it?

Tick one only:

Eeh *Yes 3*	Nyalore *Possibly 2*	Ok ang’eyo *Uncertain 1*	Ooyo *No 0*

***Narrative**: ____

____

____

If “yes” or “possibly”, enquire further, otherwise go to Q 4.

3.8 “To kapo ni chanjo ni ichiwo gi siling 325 bende di ti kode?”
If the vaccine were to cost 325 Ksh would you still take it?

Tick one only:

Eeh *Yes 3*	Nyalore *Possibly 2*	Ok ang’eyo *Uncertain 1*	Ooyo *No 0*

***Narrative**: ____

____

____

If “yes” or “possibly”, enquire further, otherwise go to Q 4.

3.9 “To kapo ni chanjo ni ichiwo gi siling 650 bende di ti kode?”
If the vaccine were to cost 650 Ksh would you still take it?

Tick one only:

Eeh *Yes 3*	Nyalore *Possibly 2*	Ok ang’eyo *Uncertain 1*	Ooyo *No 0*

***Narrative**: ____

____

4 Vignette R

“Wasebedo ka wawuoyo etuo mar Otieno/Atieno ma en ng’ama duong’, koro adwaro goyoni mbak nyathi matuo ma nyinge Omondi/Amondi…”
We have just been talking about a sick adult, Otieno/Atieno, but now let me tell you another story about a sick child, named Omondi/Amondi…

4.1 “Tuo ni iluongo nang’o? (Nying mane mar tuoni ma di pim ne go ng’at machielo?)”
What is the name of this disease? (What is the name by which you would describe the problem to someone else?)

Specify name, summary term or short description in his/her own words. If ‘other’, specify term and explain here:

***Narrative**:____

____

____

____

Types of diarrhoea	
1 Mano mana diep *Normal diarrhoea*	*5* Kipindupindu/Nyaldiema *Cholera*
2 Karenda renda *Watery diarrhoea*	*6* Tuoche moriwre *Multiple*
3 Orianyancha *Mucous diarrhoea*	*98* Mamoko, ler *Other, specify:______*
4 Odiewo remo *Bloody diarrhoea*	*99* Ok anyal wacho *Cannot say*

Code the name (one only) from the above numbered list: [ ]

Use the name as identified for this disease instead of referring to disease/problem and use the name of the person mentioned in the vignette in the following questions.

4.2 “Bende inyalo ny’isa ranyisi mamoko ma Omondi/Amondi nyalo bedo go ka waweyo ma wase wuoeyegi?”
Do *you think there are any other symptoms that Omondi/Amondi is likely to experience besides the ones we already mentioned?*

Summarize the respondent’s account of problem in his/her own words:

***Spontaneous narrative**: ____

____

____

____

Based on the respondent’s account tick problems which are mentioned under the Spon *column indicating a* spontaneous *response to the open-ended question above. Continue by probing for any categories not yet mentioned and tick them in the* Prob *column, indicating a* probed *response to screening. Make a cross when “no” or “cannot say” was the reply to probed categories. Shaded cells must not be probed.*

Physical symptoms	Spon	Prob		Spon	Prob
1 Ich maremo kabisa<br>*Severe pain/abdominal cramps*			*13* Pien joure kendo tuo<br>*Skin (loose, dry, shrivelled)*		
2 Ich kach e piny ich<br>*Low-level abdominalpain/discomfort*			*14* Dhok marach *Loss of appetite*		
3 Ich ma muorore *Muscle cramps*			*15* Wich bar *Headache*		
4 Ng’ok *Vomiting*			*16* Chuny malepo *Nausea*		
5 Dhi oko ma ng’eny *Large amounts of stool*			*17* Del machwakre *Fever*		
6 Dhi oko kinde ka kinde<br>*Frequent passing of stool*			*18* Jony<br>*Weakness*		
7 Diep machalo mchele/marochere *Rice water-like stool*			*19* Adundo gore matek *Palpitations*		
8 Diep motimo othinyo *Mucus in stool*			*20* Paro ok chung’ kare *Confusion*		
9 Diep motimo remo *Bloody stool*			*21* Paro lal *Unconsciousness*		
10 Rem e sianda<br>*Rectal pain*			*98* Ranyisi mamoko<br>*Other physical symptoms*		
11 Riyo mang’eny *Very thirsty*			*99* Ok anyal wacho *Cannot say*		
12 Wang’ modonjo iye *Sunken eyes*					

***Key question for narrative: verbatim text of voice recording required**

***Probed narrative**: ____

____

____

____

If more than one category is coded in the above table, enquire further; otherwise enter the single coded category number below, and proceed with Q 4.4:

4.3 "Kuom ranyisi gi duto, ere ma ineno ka rach moloyo?"
Among all these symptoms which one do you think would be the single most troubling?

Narrative: ____

____

____

____

Code the most troubling category from the above numbered list of patterns of distress: [ ]

4.4 "Ere kaka tuo ni nyalo yungo Omondi/Amondi gi jonyuolne e yor paro, tudruok gi jo mamoko, kata e yor pesa kalure gi dakne ma pile?"
How do you think that this [disease] will affect Omondi/Amondi and his/her household caretakers emotionally, socially and financially in their daily lives?

Summarize the respondent's account of problem in his/her own words:

***Spontaneous narrative**: ____

____

____

____

Based on the respondent's account tick problems which are mentioned under the Spon *column indicating a* spontaneous *response to the open-ended questions above. Continue by probing for any categories not yet mentioned and tick them in the* Prob *column, indicating a* probed *response to screening. Make a cross when "no" or "cannot say" was the reply to probed categories.*

**Impact (child or caretakers)**	**Spon**	**Prob**		**Spon**	**Prob**
Social impact			*Emotional impact*		
1 Riuruok gi jomoko<br>*Isolation from others*			*6* Ok obed ng'ama mor, obedo gi buok ka chiel gi paro<br>*Sadness, anxiety, worry*		
2 Luoro mar lando tuo ne jomamoko<br>*Fear of infecting others*			*Financial impact*		
3 Kelo pek e kuonde mag thieth<br>*Disruption of health services*			*7* Tiyo gi pese (e wuoth, chiemo to gi yath)<br>*Costs (transportation, foods, drugs)*		
4 Nyalo mone timo tije mapile<br>*Interference with work/daily activities*			*8* Yuto mar jo ot dok piny<br>*Loss of family income*		
5 Nyalo miye luoro mar tudruok gi jowadgi<br>*Interference with social relationships*			*Miscellaneous*		
			98 Mamoko, ler *Other, specify:______*		
			99 Ok anyal wacho *Cannot say*		

***Probed narrative**: ____

____

____

____

If more than one category is coded in the above table, enquire further; otherwise enter the single coded category number below, and proceed with Q 4.6:

4.5 "Kuom chandruok ma ichiwo gi ere malich moloyo gitee?"
Which of these problems that you have mentioned do you consider the single most troubling?

Narrative: ___

Code the most troubling category (one only) from the above numbered list of impacts: ☐

4.6 "[Tuoni] lich maromo nade ne Omondi/Amondi?"
How serious is this [disease] for Omondi/Amondi?

Tick one only:

Olioch ahinya *Very serious 3*	Olich moromo *Moderately serious 2*	Ok angeyo *Uncertain 1*	Ok olich *Not serious 0*

Narrative: ___

4.7 "Ang'o manyalo timre ne Omondi/Amondi kapo ni [tuoni] ok othiedhi?"
What is the most likely health outcome of this [disease] for Omondi/Amondi without appropriate treatment from outside?

Tick one only:

Tho<br>*Usually fatal 4*	Samoro tuo ni nyalo nege/tuoni ja negogaji<br>*Sometimes fatal 3*	Olich to ok oneki<br>*Serious but not fatal 2*	Ok ang'eyo<br>*Uncertain 1*	Ochango maber kendo piyo<br>*Full/quick recovery 0*

Narrative: ___

4.8 "Bende in, kata ng'at ma udakgo osega bedie gi [tuoni]?"
Have you or somebody else in your household ever had this [disease]?

Tick one only:

Eeh *Yes 3*	Nyalo betie *Possibly 2*	Ok an gi adier *Uncertain 1*	Ooyo *No 0*

Narrative: ___

If* yes *or* possibly, *enquire further otherwise go to Q 4.10:

4.9 "Mano ne en ng'a?"
Who was that?

Tick all that apply:

1 An *Self*	*2* Jaoda<br>*Spouse*	*3* Janyuolna<br>*Parent*	*4* Nyithindo<br>*Children*	*5* Nyithindwa<br>Sibling	*6* Jo mamoko ma wadakgo<br>*Other household member*	*7* Ok anyal wacho<br>*Cannot say*

Narrative: ___

4.10 “Ka ing’iyo, to joma nade ma hinyoga bedo gi tuo ni? Mon koso chwo? Jomadongo koso nyithindo? Jomomewo koso jo chan?”
In general, who is most likely to get this [disease]? Is it males or females? Adults or children? Rich or poor people?

Enquire about the following categories if not clear from response and tick one response only for each of the three following queries:

**Chuech *Sex***	*1* Chwo *Male*	*2* Mon *Female*	*3* Ng’ato a ng’ata *Neither*
**Higni *Age***	*1* Jomadongo *Adults*	*2* Nyithindo *Children*	*3* Ng’ato a ng’ata *Neither*
**Okang’ mar yuto *Social class***	*1* Joma omeo *Rich*	*2* Joma odhier *Poor*	*3* Ng’ato a ng’ata *Neither*

Narrative:____

4.11 “Ji neno ga gikmoko e yore mopogre o opogre. Iparo ni ang’o ma okelo ne Omondi/Amondi tuo ni?”
Each of us may explain something that happens in various ways. What do you think has caused Omondi/Amondi’s problem?

Summarize the respondent’s ideas about causes in his/her own words:

***Spontaneous narrative**: ____

Based on the respondent’s account tick perceived causes in the Spon *column indicating a* spontaneous *response to the open-ended question above. Continue by probing for any category not yet mentioned and tick them in the* Prob *column, indicating a* probed *response to screening. Make a cross when “no” or “cannot say” was the reply to probed categories*

**Perceived causes**	**Spon**	**Prob**		**Spon**	**Prob**
Ingestion			*9* Lwang’ni *Flies*		
1 Modho pi ma ok ler *Drinking contaminated water*			*10* Malaria		
2 Chamo chiemo ma owichore kata okler *Unprotected/spoiled food (biological)*			*11* Njofni *Worms*		
3 Ochamo chiemo mar kwero *Forbidden food (taboo)*			*Magico-religious causes*		
4 Chamo loo *Eating Soil*			*12* Juok *Witchcraft*		
Behaviour			*13* Dwach Nyasaye *God’s will*		
5 Tiyo gi pi maok ler *Contact with contaminated water*			*14* Koso luo chik *Violation of taboo/tradition*		
6 Ok olog ga *Not washing hands*			*Miscellaneous*		
Environment			*15* Dhoth *Breast feeding*		
7 Aluora molil *Dirty environment*			*16* Lak matii *Teething*		
8 Onge choo *Lack of latrines/toilets*			*98* Mamoko, ler *Other, Specify*		
			99 Ok anyal wacho *Cannot say*		

***Probed narrative**: ____

If more than one category is coded in the above table, enquire further; otherwise enter the single coded category number below, and proceed with Q 4.13:

4.12 “Kuom yore ma isewachonagi, ere ma ineno ka en yoo maduong manyalo kelo tuoni?”
Which one of these causes that you have mentioned do you consider the main cause?

Narrative:____

Code the most important category (one only) from the above numbered list of perceived causes: ☐

***Key question for narrative: verbatim text of voice recording required**

4.13 “Ang’o ma ji timo ne ga ji manigi tuo kamar Omondi/Amondi ei dala kapok gidhi yudo thieth kata kony oko mar dala?”
What do people do at home for someone with this [disease] like Omondi/Amondi before looking for treatment or help outside their homes?

Summarize the respondent’s account of home-based treatment in his/her own words:

***Spontaneous narrative**: ____________________

____

____

____

Based on the respondent’s account tick home-based treatment categories in the Spon *column indicating a* spontaneous *response to the open-ended question above. Continue by probing for any home-based treatment categories not yet mentioned and tick them in the* Prob *column, indicating a* probed *response to screening. Make a cross when “no” or “cannot say” was the reply to probed categories.*

Home-based treatment	Spon	Prob
1 Omodho pi kata gik mimadho mang’eny *Drinking more water or liquids*		
2 Omadho yadh nyaluo *Herbal treatment (roots, bark, leaves)*		
3 Omadho yedhe mag duka ma medo pi kaka ORS *Oral rehydration therapy*		
4 Lamo *Prayers*		
5 Ngato ngiewo yath e duka kata famasi to muonyo *Self-administered antibiotics/drugs*		
6 Onge *Nothing*		
7 Madho kong’o kata chang’aa *Drink containing alcohol*		
98 Mamoko, ler Other, specify:______		
99 Ok anyal wacho Cannot say		

***Probed narrative**: ____________________

____

____

____

If more than one category is coded in the above table, enquire further; otherwise enter the single coded category number below, and proceed with Q 4.15:

4.14 “Kuom thieth ma ji chiwo ei dala, mane ma iparo ni nigi kony moloyo?”
Which one of all these things people do at home do you think is likely to be most helpful?

Narrative: ____________________

____

____

____

Code the most helpful category (one only) from the above numbered list of home-based treatments: ☐

4.15 “Ere kama Omondi/Amondi inyalo ter e thieth oko mar dala?”
Where will Omondi/Amondi usually be taken for treatment outside his/her home?

Summarize the respondent’s account of outside treatment in his/her own words:

***Spontaneous narrative**: ____________________

____

____

____

Based on the respondent's account tick outside treatment categories in the Spon *column indicating a* spontaneous *response to the open-ended question above. Continue by probing for any outside treatment categories not yet mentioned and tick them in the* Prob *column, indicating a* probed *response to screening. Make a cross when "no" or "cannot say" was the reply to probed categories.*

**Outside treatment**	**Spon**	**Prob**
1 Osiptal *Health facilities*		
2 Jo thieth nyaluo *Traditional healers*		
3 Famisi kata kuonde mi use yath *Pharmacy or over-the-counter drugs*		
4 Jolemo *Faith healers (Pastors, Imams, Sheikhs)*		
5 Yudo thieth kuom nyamreche, wede kata osiepe *Informal help from health-worker, friend/relative*		
98 Kuonde ma moko, ler *Other, specify:*______		
99 Ok anyal wacho *Cannot say*		

***Probed narrative**: ____

____

____

____

If more than one category is coded in the above table, enquire further; otherwise enter the single coded category number below, and proceed with Q 4.17:

4.16 "Kuom jogi duto, ere ngato manyalo chiwo kony kabisa mohingo mokogo?"
Which one of these people they might consult do you think is most helpful?

Narrative: ____

____

____

____

Code the most helpful category from the above numbered list of outside treatments: ☐

4.17 "Bende iparo ni min Omondi/Amondi kata jo dalagi ok onego ogol wach [tuoni] ne jo maok jodalano?"
Do you think Omondi/Amondi's mother or family should not disclose this [disease] outside the closest family?

Tick one only:

Eeh *Yes 3*	Nyalore/jomoko *Possible/mixed 2*	Ok ong'ere *Uncertain 1*	Ooyo *No 0*

Narrative: ____

____

____

4.18 "To ka po ni ji ong'eyo, iparo ni jomoko nyalo miyo jood gi Omondi/Amondi wich kuot kalure gi [tuoni]?"
If they knew, do you think some people might make the family of [this child] feel ashamed or embarrassed because of this [disease]?

Tick one only:

Eeh *Yes 3*	Nyalore/jomoko *Possible/mixed 2*	Ok ong'ere *Uncertain 1*	Ooyo *No 0*

Narrative: ____

____

____

4.19 "Iparoni ka jomoko ofwenyo wach [tuoni] to nyalo kelo ne Omondi/Amondi chandruok
Would others finding out about this [disease] cause problems for Omondi/Amondi?

Tick one only:

Eeh *Yes 3*	Nyalore/jomoko *Possible/mixed 2*	Ok ong'ere *Uncertain 1*	Ooyo *No 0*

Narrative:____

____

____

4.20 "To kaponi jomoko ong'eyo wach [tuoni], iparo ni ginyalo miyo joodgi Omondi/Amondi chandruok?"
Would others finding out about this [disease] cause problems for the family of Omondi/Amondi?

Tick one only:

Eeh *Yes 3*	Nyalore/jomoko *Possible/mixed 2*	Ok ong'ere *Uncertain 1*	Ooyo *No 0*

Narrative:____

____

____

4.21 "Bende nyalo bedo ni nitiere ngato ei od gi Omondi/Amondi manyalo neno pek mar tere e thieth nikech ok gi dwa ni wach [tuoni] olandre?"
Might there be someone in the household who would hesitate to bring Omondi/Amondi to treatment because they did not want the [disease] to be known?

Tick one only:

Eeh *Yes 3*	Nyalore/jomoko *Possible/mixed 2*	Ok ong'ere *Uncertain 1*	Ooyo *No 0*

Narrative:____

____

____

4.22 "Kajomamoko ma ok jodalano ong'eyo wach [tuoni], iparo ni mano nyalo kelo ne Omondi/Amondi kachiel gi joodgi konyruok?"
Is it likely that others outside the family finding out about this [disease] would be helpful to the child and the family?"

Tick one only:

Eeh *Yes 0*	Nyalore/jomoko *Possible/mixed 1*	Ok ong'ere *Uncertain 2*	Ooyo *No 3*

Narrative:____

____

____

4.23 "Ang'o ma inyalo tim mondo ogeng' [tuoni]?"
What can be done to prevent this [disease]?

Summarize the respondent's account of prevention options in his/her own words:

***Spontaneous narrative**: ____

____

____

____

Based on the respondent's account tick prevention categories in the Spon *column indicating a* spontaneous *response to the open-ended question above. Continue by probing for any prevention categories not yet mentioned and tick them in the* Prob *column, indicating a* probed *response to screening. Make a cross when "no" or "cannot say" was the reply to probed categories.*

**Prevention**	**Spon**	**Prob**
1 Luoko lwedo *Wash hands*		
2 Modho pi mochwaki kata moket yath *Safe water*		
3 Chamo chiemo maler *Clean/safe food*		
4 Wito yugi e yo ma kare *Safe disposal of garbage*		
5 Losruok eyo makare *Safe disposal of stool*		
6 Muony yien mageng'o tuoni *Preventive drugs*		
7 Chanjo *Vaccines*		
8 Puonjruok e weche molure gi ngima *Health education*		
9 Thieth gi yien nyaluo *Protection from supernatural influence (charms etc)*		
98 Mamoko, ler *Other, specify:______*		
99 Ok anyal wacho/onge *Cannot say/Nothing*		

***Probed narrative**: ____

____

____

____

If more than one category is coded in the above table, enquire further; otherwise enter the single coded category number below, and proceed with Q 5:

4.24 "Kuom yore gi duto, ere yo miparo ni ber kabisa e geng'o [tuoni]?"
Which one of these ways of prevention do you think is most useful?

Narrative: ____

____

____

____

Code the most useful way from the above numbered list of preventive measures: ☐

5 Vaccines – R

5.1 "Kapo ni Omondi/Amondi en nyathini, to nitie chanjo ma itone e dhoge momuonyo mondo ogeng' tuo mar diep mar nyithindo, be di tere Omondi/Amondi mondo omiye yathni?"
If Omondi/Amondi was your child, and if the child could have been given a vaccine, a few drops to swallow, as an infant to prevent this illness, would you have taken Omondi/Amondi to get this vaccine?

Tick one only:

Eeh *Yes 3*	Nyalore *Possibly 2*	Ok ong'ere *Uncertain 1*	Ooyo *No 0*

***Narrative**: ____

____

____

____

If "yes" or "possibly", enquire further, otherwise go to Q 6.

5.2 "Kaponi ni chanjo ni en siling 65, bende pod diter nyathini mondo ochanje?"
If the vaccine were to cost 65 Ksh would you still take the child to get it?

Tick one only:

Eeh *Yes 3*	Nyalore *Possibly 2*	Ok ong'ere *Uncertain 1*	Ooyo *No 0*

***Narrative**: ____

____

____

If "yes" or "possibly", enquire further, otherwise go to Q 6.

5.3 "Kaponi chanjo ni en siling 325, bende pod diter nyathini mondo ochanje?"
If the vaccine were to cost 325 Ksh, would you still take the child to get it?

Tick one only:

Eeh *Yes 3*	Nyalore *Possibly 2*	Ok ong'ere *Uncertain 1*	Ooyo *No 0*

***Narrative**: ______________________________

____

____

If "yes" or "possibly", enquire further, otherwise go to Q 6.

5.4 "Kaponi chanjo ni en siling 650, bende pod diter nyathini mondo ochanje?"
If the vaccine were to cost 650 Ksh would you still take the child to get it?

Tick one only:

Eeh *Yes 3*	Nyalore *Possibly 2*	Ok ong'ere *Uncertain 1*	Ooyo *No 0*

***Narrative**: ______________________________

____

____

6 Concluding advice from respondent

6.1 "Wase penji penjo mang'eny molure gi touché mag diep embekni ariyo gi, to gi pachi ewi tiyo gi chanjo e geng'o gi. King'iyo mbaka mokwongo ewi, Otieno/Atieno to gi mar ariyo ewi Omondi/Amondi maen mar nyathi. Kuom magi duto, mane ma iparo ni ber gi chanjo ahinya."
We have now asked you many questions about these two cases of diarrheal illness and how important you think a vaccine might be to prevent each of them. If you think back about the first problem affecting the adult Otieno/Atieno, and the second problem of the child Omondi/Amondi, for which of these do you think a vaccine would be most important.

Tick one only:

Mbaka mar Otieno/Atieno<br>Vignette C *3*	Mbaka mar Omondi/Amondi<br>Vignette R *2*	Mbekni go duto<br>All Important *1*	Onge<br>None Important 0	Ok angeyo<br>No idea/cannot say 99

***Narrative**: ______________________________

____

____

6.2 "Be nde nitie gimoro machielo madi wachna kaluore gi chandruoge mag ngima machalo gi mawawuoyegi kachiel gi ngeyoni ewi chanjo? Warwako wach moro amora madi medi, puonj kata mana paro"
Is there anything else you can tell me about the health problems we have discussed or about your experience with vaccinations? Any further comments, advice or suggestions will be appreciated.

***Narrative**: ______________________________

____

____

Team (*circle appropriate*)	Kisumu: A B ; Siaya: C D
Interview end time (hh:mm)	
Interviewer (name/signature)	
Data recorder (name/signature)	
Taped? (tick) — Yes / No / partial	
File name on recorder	
Controlled (date/initials)	
Narratives translated & typed (date)	
1st Data entry (date/initials)	
2nd Data entry (date/initials)	

Additional comments from the interview team

Notes concerning the participant's interest and the quality of the interview, and other noteworthy features and details of this interview:
