# Supplementary material for: Comparing sociocultural features of cholera in three endemic African settings
Source: BMC Med. 2013 Sep 18;11:206. doi: 10.1186/1741-7015-11-206 (PMC4016292; doi:10.1186/1741-7015-11-206)
Supplement: Additional file 3 — EMIC interview for study of community views of cholera in Zanzibar. [file 1741-7015-11-206-S3.pdf]

OCV SEB Study: Explanatory Model Interview Catalogue (EMIC)

Survey Phase: 1, Study Level: IV

Ministry of Health and Social Welfare of Zanzibar,
in collaboration with
World Health Organization, Geneva, and Swiss Tropical Institute, Basel

Swahili/English Version of 27/06/2008 (Final)

Date interview (dd-mm-yyyy)	

EMIC ID (1-X-yyy) *X: 1=Unguja, 2=Pemba*	1-___-___ ___ ___

General information

Tick appropriate:

Sex	*1* F	*2* M

Approximate age (years)	

Tick one only:

Site	*1* Unguja	*2* Pemba
Pregnant	*1* Yes	*2* No

Introduction

"Asante kwa kukubali kuzungumza na mimi leo. Ningependa kuuliza maswali kuhusu matatizo ya kiafya, ambayo yanaweza kuathiri jamii yako. Inawezekana kwamba unayajua matatizo haya au hujawahi kuyaona. Kwa vyovyote ningependa kujua mawazo yako kuhusu swala hilo. Lakini kwanza ningependa kukuuliza maswali kuhuhu maisha yako."
Thank you for letting me speak with you today. I will be asking you questions about health problems that could affect people in your community. You may recognise these conditions, or they may be unfamiliar. In either case I would like to understand your ideas about it. Your answers and thoughts will help us to assist people who have these problems. But first, a few questions about your background.

1 Socio-economic and demographic information

1.1 Hali ya ndoa *Marital status*

Tick one only:

1 Sijawahi kuoa au kuolewa *Never married*	*2* Nimeoa au nimeolewa *Married*	*3* Nimetengana *Separated*	*4* Nimeachika *Divorced*	*5* Tunaishi pamoja *Living together*	*6* Nimefiliwa na mume au mke *Widowed*	*7* Siwezi kusema *Cannot say*

1.2 Ukubwa wa nyumba *Household size*

Idadi ya watu unaishi nao *Number of people living in the household* ☐

1.3 Watoto wanaoishi katika nyumba yako *Children living in the household*

Enter 0 when answer is none for a given category:

Idadia ya watoto *Number of children*	Chini ya miaka 5 *< 5 yrs*	Miaka 5 mpaka 10 *5-10 yrs*	Miaka 10 mpaka 17 *10-17 yrs*
Wavulana *Boys*			
Wasichana *Girls*			

1.4 Uhusiano na msimamizi wa nyumba *Relationship with household head*

Tick one only:

1 Mwenyewe *Self*	*2* Mke/mume *Spouse*	*3* Wazazi *Parent*	*4* Ndugu *Sibling*	*5* Mtoto *Offspring*	*6* Uhusiano mwingine *Other specify:*

Tick sex of household head:

Sex	*1* F	*2* M

1.5 Kazi au ajira *Main occupational status*

Tick one only:

1 Kilimo *Agriculture*	*2* Uvuvi *Fishing*	*3* Nimejiajiri *Self-employment (not 1&2)*	*4* Kuajiriwa rasmi *Formally employed*	*5* Mama wa nyumbani *Housewife*
6 Mfanyakazi wa nyumbani *Housemaid*	*7* Kibarua *Casual labourer*	*8* Mwanafunzi *Student*	*9* Sifanji kazi/nimestaafu *Not active/retired*	*10*Mengine, eleza *Other specify:*

1.6 Elimu *Education*

Tick highest achieved level only:

1 Sijasoma *No education*	*2* Madrassa ya Koran *Koranic school*	*3* Elimu ya msingi *Primary school*	*4* Elimu ya sekondari *Secondary school*	*5* Elimu amali *Vocational school*	*6* Elimu juu *Higher education*

1.7 Miaka ya kusoma *Years of education*

☐ Siwezi kusema *Cannot say* ☐

1.8 Dini *Religion*

Tick one only:

1 Mwislamu *Muslim*	*2* Mkristo *Christian*	*3* Mengine, eleza *Other specify:*	*4* Sielezi *Undisclosed*

1.9 Uraia *Nationality*

Tick one only:

1 Mtanzania *Tanzanian*	*2* Raia wa nchi nyingine, eleza *Other specify:*

1.10 "Kipato cha watu wanaoishi nyumbani kwako ni cha uhakika?" *Is your household income usually reliable (and dependable)?*

Tick one only, not disclosed means uncertain:

Ndio *Yes 3*	Labda *Possibly 2*	Hakijulikani *Uncertain 1*	Hapana *No 0*

Narrative:____

____

____

____

If "yes" or "possibly", enquire further, otherwise go to Q 1.12:

1.11 "Ni kipi chanzo kikuu cha kipato katika nyumba yako?" *What main sources of income are there in your household?*

Narrative:____

____

____

____

Tick all that apply:

**Sources of income**	**Own**	**Others**
1 Kuajiriwa kwa mshahara *Employment for cash*		
2 Kuajiriwa kwa namna nyingine *Employment in kind*		
3 Kujiajiri mwenyewe katika sekta yoyote isiyo ya kilimo *Non-farm self-employment*		
4 Kuuza mayao ya kilimo *Selling agricultural produce*		
5 Kuuza samaki na mazao ya baharini *Selling fish and seafood*		
6 Kukodisha (nyumba, shamba, duka) *Rent*		
7 Msaada kututoka nje *Remittances (money sent from outside)*		
8 Pensheni *Pension*		
98 Nyinginezo, eleza *Other specify:* /		
99 Siwezi kusema *Cannot say*		

1.12 "Ni kiasi gani cha pesa umepata wewe mwenyewe mwezi uliopita? Na mume wako je? Na watu wengine wanaokaa nyumbani kwako?"
How much money did you make during the last month on your own? And what about your spouse and other household members?

Narrative:____

____

____

____

Query for items not mentioned, clarify if needed. If there is no income, enter 0 in TSh column. If respondent is widowed, then tick "cannot say" for category 2:

Monthly income	TSh		
1 Chake mwenyewe *Own*		Siwezi kusema *Cannot say*	☐
2 Cha mume/mke wake *Spouse*		Siwezi kusema *Cannot say*	☐
3 Cha watu wengine wanaoishi katika nyumba hii hii *Additional household income*		Siwezi kusema *Cannot say*	☐

Introduction to vignettes

"Nakushukuru kwa kukubali kuzungumza na mimi kuhusu matatizo ya afya yanayoweza kuathiri jamii. Sasa ningependa sana kujua maoni yako kuhusu swala hilo. Tafadhali usione haya kujieleza. Nitakusimulia hadithi mbili tofauti ya watu ambao wana matatizo maalum."
I appreciate your willingness to talk to me about a few health problems that affect people in your community. I want to understand how you think about it. It is your ideas that I am interested in so please don't feel shy to tell me your personal opinion. I will tell you two different stories about persons who are having a particular problem.

2 Vignette A

"Sikiliza hadithi ya [mtu huyu]…" *Let me tell you the story about this [person]…*

2.1 "Maradhi haya yanaitwaje?"
What is the name of this disease?

Specify name, summary term or short description in his/her own words. If 'other', specify term and explain here:

Narrative:____

____

____

____

Types of diarrhoea	
1 Kuharisha kawaida *Normal diarrhoea*	*5* Kipindupindu *Cholera*
2 Kuharisha maji *Watery diarrhoea*	*6* Mchanganyiko *Multiple*
3 Kuharisha marenda *Mucous diarrhoea*	*98* Mengineyo, eleza *Other, specify*:
4 Kuharisha damu *Bloody diarrhoea*	*99* Siwezi kusema *Cannot say*

Code the name from the above numbered list: ☐

Use the name as identified for this disease instead of referring to disease/problem and use the name of the person mentioned in the vignette in the following questions.

2.2 "Je unaweza kufikiria dalili nyingine ambazo [mtu huyu] anaweza kuwa nazo mbali na tulizozitaja hapo juu?"
Can you think of any other symptoms that this [person] is likely to experience besides the ones we already mentioned?

Summarize the respondent's account of problem in his/her own words:

Spontaneous narrative: ____

____

____

____

Based on the respondent's account tick problems which are mentioned under the Spon *column indicating a* spontaneous *response to the open-ended question above. Continue by probing for any categories not yet mentioned and tick them in the* Prob *column, indicating a* probed *response to screening. Make a cross when "no" or "cannot say" was the reply to probed categories. Shaded cells must not be probed.*

**Physical symptoms**	**Spon**	**Prob**		**Spon**	**Prob**
1 Kusokotwa na tumbo *Abdominal cramps*			*13* Kunyauka, kukauka ngozi *Skin (loose, dry, shrivelled)*		
2 Kuumwa na tumbo *Abdominal pain/discomfort*			*14* Kutokuwa na hamu ya kula *Loss of appetite*		
3 Kuumwa na misuli *Muscle cramps*			*15* Maumiva ya kitchwa *Headache*		
4 Kutapika *Vomiting*			*16* Kichefuchefu *Nausea*		
5 Choo kingi kupita kiasi *Large amounts of stool*			*17* Homa *Fever*		
6 Kuharisha mara kwa mara *Frequent passing of stool*			*18* Udhaifu *Weakness*		
7 Choo kama maji ya mchele *Rice water-like stool*			*19* Kupaparikwa na moyo *Palpitations*		
8 Choo chenye usaha *Pus in stool*			*20* Fadhaisha *Confusion*		
9 Choo chenye damu *Bloody stool*			*21* Kupoteza fahamu *Unconsciousness*		
10 Maumivu sehemu ya kunyea *Rectal pain*			*98* Dalili nyingine mwilini *Other physical symptoms*		
11 Kiu kali *Very thirsty*			*99* Siwezi kusema *Cannot say*		
12 Macho kuingia ndani *Sunken eyes*					

Probed narrative: ____

____

____

____

If respondent has mentioned more than one category, enquire further, otherwise go to Q 2.4:

2.3 "Je, kati ya hizo dalili ipi inayosumbua zaidi kuliko zote?"
Among all these symptoms which one do you consider the single most troubling?

Narrative: ____

____

____

____

Code the most troubling category from the above numbered list of patterns of distress: [ ]

2.4 "Je unafikiri maradhi haya yatamuathiri vipi [mtu huyu] kimawazo, kijamii na kiuchumi katika maisha yake ya kila siku?"
How do you think that this [disease] will affect [this person] emotionally, socially and financially in his/her daily life?

Summarize the respondent's account of problem in his/her own words:

Spontaneous narrative: ____

____

____

____

Based on the respondent's account tick problems which are mentioned under the Spon *column indicating a* spontaneous *response to the open-ended questions above. Continue by probing for any categories not yet mentioned and tick them in the* Prob *column, indicating a* probed *response to screening. Make a cross when "no" or "cannot say" was the reply to probed categories.*

**Impact**	**Spon**	**Prob**		**Spon**	**Prob**
Social impact			*Emotional impact*		
1 Kutengwa na watu wengine<br>*Isolation from others*			*6* Huzuni, kukosa raha, wasiwasi<br>*Sadness, anxiety, worry*		
2 Woga wa kuambukiza wengine<br>*Fear of infecting others*			*Financial impact*		
3 Kusitisha huduma za afya<br>*Disruption of health services*			*7* Kuongezeka kwa gharama za maisha<br>*Costs (transportation, foods, drugs)*		
4 Kuathiri shudhuli za kila siku<br>*Interference with work/daily activities*			*8* Kupoteza kipato cha familia<br>*Loss of family income*		
5 Kuathiri uhusiano na watu wengine katika jamii<br>*Interference with social relationships*			*Miscellaneous*		
			98 Mengine, eleza *Other, specify:*		
			99 Siwezi kusema *Cannot say*		

Probed narrative: ______________________________

____

____

____

If respondent has mentioned more than one category, enquire further, otherwise go to Q 2.6:

2.5 "Kati ya matatizo yaliyotajwa hapo juu, tatizo lipi haswa linasumbua zaidi kuliko yote?"
Which of these problems that you have mentioned do you consider the single most troubling?

Narrative: ______________________________

____

____

____

Code the most troubling category from the above numbered list of impacts: [ ]

2.6 "Unaonaje ubaya wa [maradhi haya] kwa [mtu huyu]?"
How serious is this [disease] for [this person]?

Tick one only:

Mbaya sana *Very serious 3*	Mbaya kiasi *Moderately serious 2*	Haitabiriki *Uncertain 1*	Si mbaya *Not serious 0*

Narrative: ______________________________

____

____

____

2.7 "Je afya ya [mtu huyu] itakuwaje ikiwa atakosa matibabu sahihi ya [maradhi haya] kutoka nje ya nyumba yake?"
What is the most likely health outcome of this [disease] for [this person] without appropriate treatment from outside?

Tick one only:

Kifo<br>*Usually fatal 4*	Wakati mwingine inaweza kusababisha kifo<br>*Sometimes fatal 3*	Hali mbaya sana, lakini haisababishi kifo<br>*Serious but not fatal 2*	Haitabiriki<br>*Uncertain 1*	Kupona vizuri na haraka<br>*Full/quick recovery 0*

Narrative: ______________________________

____

____

____

2.8 "Je, kuna yeyote nyumbani kwako aliyewahi kupata [maradhi haya]?"
Have you or somebody else in your household ever had this [disease]?

Tick one only:

Ndio *Yes 3*	Inawezekana *Possibly 2*	Hakuna hakika *Uncertain 1*	Hapana *No 0*

Narrative:____

If yes *or* possibly, *enquire further otherwise go to Q 2.10:*

2.9 "Je, alikuwa nani?"
Who was that?

Tick all that apply:

1 Mimi mwenyewe *Self*	*2* Mke/Mume *Spouse*	*3* Wazazi *Parent*	*4* Watoto *Children*	*5* Dada/kaka/mdogo wangu *Sibling*	*6* Watu wengine wanaoishi ndani ya nyumba hii *Other household member*	*99* Siwezi kusema *Cannot say*

Narrative:____

2.10 "Je, kwa kawaida ni nani mara nyingi hupata [maradhi haya]? Akina baba au akina mama? Watu wazima au watoto? Matajiri au watu masikini?"
In general, who is most likely to get this [disease]? Is it men or women? Adults or children? Rich or poor people?

Enquire about the following categories if not clear from response and tick all that apply:

**Jinsia *Sex***	*1* Wanaume *Men*	*2* Wanawake *Women*	*3* Si yeyote *Neither*
**Umri *Age***	*1* Watu wazima *Adults*	*2* Watoto *Children*	*3* Si wowote *Neither*
**Hali ya maisha *Social class***	*1* Matajiri *Rich*	*2* Watu masikini *Poor*	*3* Si yoyote *Neither*

Narrative:____

2.11 "Kila mmoja wetu anaweza kueleza mambo yanayotokea kwa njia tofauti tofauti. Je unafikiria nini kimesababisha tatizo la [mtu huyu]?"
Each of us may explain something that happens in various ways. What do you think has caused this [person]'s problem?

Summarize the respondent's ideas about causes in his/her own words:

Narrative:____

Based on the respondent's account tick perceived causes in the Spon *column indicating a* spontaneous *response to the open-ended question above. Continue by probing for any category not yet mentioned and tick them in the* Prob *column, indicating a* probed *response to screening. Make a cross when "no" or "cannot say" was the reply to probed categories.*

**Perceived causes**	**Spon**	**Prob**		**Spon**	**Prob**
Ingestion			*8* Nzi *Flies*		
1 Kunywa maji machafu *Drinking contaminated water*			*9* Malaria		
2 Chakula ambacho hakijahifadhiwa/kimeoza *Unprotected/spoiled food (biological)*			*10* Minyoo *Worms*		
3 Chakula kilichokatazwa *Forbidden food (taboo)*			*Magico-religious causes*		
4 Kula udongo *Eating Soil*			*11* Uchawi *Witchcraft*		
Behaviour			*12* Rehema ya *Mungu God's will*		
5 Kugusa maji machafu *Contact with contaminated water*			*Miscellaneous*		
6 Kutoosha mikono *Not washing hands*			*98* Mengine, eleza *Other, specify:*		
Environment			*99* Siwezi kusema *Cannot say*		
7 Mazingira machafu *Dirty environment*					

Probed narrative: ____

____

____

____

If respondent has mentioned more than one category, enquire further, otherwise go to Q 2.13:

2.12 "Je, kati ya hizo sababu, ipi ni sababu kuu?"
Which one of these causes that you have mentioned do you consider the main cause?

Narrative:____

____

____

____

Code the most important category from the above numbered list of perceived causes: [ ]

2.13 "Je, watu humsaidia nini nyumbani [mtu] mwenye [maradhi haya] kabla ya kutafuta matibabu ya nje ya nyunbani kwao?"
What do people do at home for a [person] with this [disease] before looking for treatment or help outside their homes?

Summarize the respondent's account of home-based treatment in his/her own words:

Spontaneous narrative:____

____

____

____

Based on the respondent's account tick home-based treatment categories in the Spon *column indicating a* spontaneous *response to the open-ended question above. Continue by probing for any home-based treatment categories not yet mentioned and tick them in the* Prob *column, indicating a* probed *response to screening. Make a cross when "no" or "cannot say" was the reply to probed categories.*

**Home-based treatment**	**Spon**	**Prob**
1 Kunywa maji mengi au vinywaji vingine *Drinking more water or liquids*		
2 Dawa za mitishamba (mizizi, magamba, majani) *Herbal treatment (roots, bark, leaves)*		
3 Kunywa dawa za vipaketi (ORS) zinazouzwa madukani *Oral rehydration therapy*		
4 Kuomba dua *Prayers*		
5 Kutumia dawa za antibiotics za kujinunulia mwenyewe *Self-administered antibiotics/drugs*		
6 Hapana *Nothing*		
98 Mengine, eleza *Other, specify:*		
99 Siwezi kusema *Cannot say*		

Probed narrative: ____

____

____

____

If respondent has mentioned more than one category, enquire further, otherwise go to Q 2.15:

2.14 "Je, kati ya haya matibabu, tiba ipi inafaa zaidi kuliko yote?"
Which one of all these things people do at home do you think is likely to be most helpful?

Narrative:____

Code the most helpful category from the above numbered list of home-based treatments: ☐

2.15 "[Mtu] mwenye [maradhi haya] kwa kawaida ataenda wapi kupata matibabu nje ya nyumba yake?"
Where will this person usually go for treatment outside his/her home?

Summarize the respondent's account of outside treatment in his/her own words:

Spontaneous narrative:____

Based on the respondent's account tick outside treatment categories in the Spon *column indicating a* spontaneous *response to the open-ended question above. Continue by probing for any outside treatment categories not yet mentioned and tick them in the* Prob *column, indicating a* probed *response to screening. Make a cross when "no" or "cannot say" was the reply to probed categories.*

**Outside treatment**	**Spon**	**Prob**
1 Hospitali *Health facilities*		
2 Waganga wa kienyeji *Traditional healers*		
3 Maduka ya madawa *Pharmacy or over-the-counter drugs*		
4 Viongozi vya dini *Faith healers (Imams, Sheikh)*		
5 Ushauri kutoka kwa ndugu/jamaa na marafiki wanaofanya kazi vituo vya afya<br>*Informal help from health-worker, friend/relative*		
98 Sehemu nyinginezo, eleza *Other, specify:*		
99 Siwezi kueleza *Cannot say*		

Probed narrative: ____

If respondent has mentioned more than one category, enquire further, otherwise go to Q 2.17:

2.16 "Je, kati ya watu hawa wanaoombwa ushauri (na wagonjwa) yupi anafaa zaidi kuliko wote?"
Which one of these people they might consult do you think is most helpful?

Narrative:____

Code the most helpful category from the above numbered list of outside treatments: ☐

2.17 "Je, unafikiri [mtu huyu] ni vizuri asiwaambie watu wengine zaidi ya watu wa familia yake kuhusu [maradhi haya]?"
Do you think [this person] should not disclose [this disease] beyond his/her closest family?

Tick one only:

Ndio *Yes 3*	Labda/mchanganyiko *Possible/mixed 2*	Haijulikani *Uncertain 1*	Hapana *No 0*

Narrative:____

2.18 “Je, watu wengine wangejua habari za [mtu huyu] wangemfanya aone aibu kwa sababu ya [maradhi haya]?”
If they knew, do you think some people might make [this person] feel ashamed or embarrassed because of [this disease]?

Tick one only:

Ndio *Yes 3*	Labda/mchanganyiko *Possible/mixed 2*	Haijulikani *Uncertain 1*	Hapana *No 0*

Narrative:____

2.19 “Je, watu wengine wangejua kwamba ameugua [maradhi haya] ingesababisha matatatizo kwa [mtu huyu]?”
Would others finding out about [this disease] cause problems for [this person]?

Tick one only:

Ndio *Yes 3*	Labda/mchanganyiko *Possible/mixed 2*	Haijulikani *Uncertain 1*	Hapana *No 0*

Narrative:____

2.20 “Je watu wengine wangejua kwamba ameugua [maradhi haya] ingesababisha matatizo kwa familia ya [huyu mtu]?”
Would others finding out about [this disease] cause problems for the family of [this person]?

Tick one only:

Ndio *Yes 3*	Labda/mchanganyiko *Possible/mixed 2*	Haijulikani *Uncertain 1*	Hapana *No 0*

Narrative:____

2.21 “Je, kuna mtu yeyote katika familia yake ambaye angewacha kumpeleka kupata matibabu kwa vile hataki watu wengine wajue kwamba [mtu huyu] ameugua [maradhi haya]?”
Might there be someone in the household who would hesitate to bring [this person] to treatment because they did not want the [disease] to be known?

Tick one only:

Ndio *Yes 3*	Labda/mchanganyiko *Possible/mixed 2*	Haijulikani *Uncertain 1*	Hapana *No 0*

Narrative:____

2.22 “Je, inawezekana kwamba watu wengine nje ya familia wakasaidia, wakijua kwamba [mtu huyu] ameugua [maradhi haya]?”
Is it likely that others outside the family finding out about [this disease] would be helpful to [this person]?”

Tick one only:

Ndio *Yes 0*	Labda/mchanganyiko *Possible/mixed 1*	Haijulikani *Uncertain 2*	Hapana *No 3*

Narrative:____

2.23 “Je, nini kitafanyika kujikinga na [maradhi haya]?“
What can be done to prevent this [disease]?

Summarize the respondent’s account of prevention options in his/her own words:

Spontaneous narrative: ____________________

____

____

____

Based on the respondent’s account tick prevention categories in the Spon *column indicating a* spontaneous *response to the open-ended question above. Continue by probing for any prevention categories not yet mentioned and tick them in the* Prob *column, indicating a* probed *response to screening. Make a cross when “no” or “cannot say” was the reply to probed categories.*

**Prevention**	**Spon**	**Prob**
1 Kuosha mikono *Wash hands*		
2 Maji yaliyochemshwa au yenye dawa *Safe water*		
3 Chakula safi na salama *Clean/safe food*		
4 Utupaji na uwekaji wa takataka vizuri *Safe disposal of garbage*		
5 Uhifadhi wa kinyesi vizuri *Safe disposal of stool*		
6 Dawa za kinga *Preventive drugs*		
7 Chanjo *Vaccines*		
8 Elimu ya afya *Health education*		
98 Mengine, eleza *Other, specify:*		
99 Siwezi kusema/hapana *Cannot say/Nothing*		

Probed narrative: ____________________

____

____

____

If respondent has mentioned more than one category, enquire further, otherwise go to Q 3.1:

2.24 “Je, kati ya hizo njia ya kujikinga na [maradhi haya], ni njia ipi inafaa zaidi kuliko zote?”
Which one of these ways of prevention do you think is most useful?

Narrative: ____________________

____

____

____

Code the most useful way from the above numbered list of preventive measures: ☐

3 Vaccines

3.1 “Je, wewe umeshawahi kupata chanjo ya aina yoyote au mtu mwingine yeyote katika familia yako?”
Have you or anyone in your household received any type of vaccine?

Tick one only:

Ndio *Yes 3*	Labda *Possibly 2*	Hakijulikani *Uncertain 1*	Hapana *No 0*

Narrative: ____________________

____

____

____

If “yes” or “possibly”, enquire further, otherwise go to Q 3.3:

3.2 “Nani alipata chanjo?“
Who got it?

Tick all that apply:

1 Mimi mwenyewe *Self*	*2* Watoto *Children*	*3* Watu wazima wanaokaa nyumba hii *Adults in household*

Narrative:____

____

____

____

3.3 “Je, unafikiri kutokana na uzoefu wako, chanjo husaidia?”
Based on your experience, do you think vaccines are generally helpful?

Tick one only:

Ndio *Yes 3*	Wakati mwingine *Sometimes 2*	Hakijulikani *Uncertain 1*	Hapana *No 0*

Narrative:____

____

____

____

3.4 “Je, unafikiri chanjo inaweza kusababisha matatizo?”
Do you think some vaccines are also likely to cause problems?

Tick one only:

Ndio *Yes 3*	Labda *Possibly 2*	Hakijulikani *Uncertain 1*	Hapana *No 0*

Narrative:____

____

____

____

If “yes” or “possibly”, enquire further, otherwise go to Q 3.6:

3.5 “Tafadhali nieleze zaidi kuhusu hilo.“
Please tell me about that.

Tick all that apply:

**Problems caused by vaccines**	**Tick**
1 Kuvimba/maumivu sehemu iliyochomwa sindano *Pain/swelling at injection site*	
2 Homa *Fever*	
3 Majipu/kidonda *Infection/abscess*	
4 Kovu *Scar*	
5 Mtoto kulia sana *Crying baby*	
98 Mengine, eleza *Other, specify:*	

Narrative:____

____

____

____

3.6 “Ikiwa chanjo kwa ajili ya kujikinga na kipindupindu itatolewa bila ya malipo utakuwa tayari kunywa?”
If a vaccine that you swallow becomes available to prevent cholera, would you take it if it was made available without charge?

Tick one only:

Ndio *Yes 3*	Labda *Possibly 2*	Hakijulikani *Uncertain 1*	Hapana *No 0*

Narrative:____

If "yes" or "possibly", enquire further, otherwise go to Q 4.1:

3.7 "Kama chanjo ya kipindupindu ingegharimu 1,000 TSh ungeitumia?"
If the vaccine were to cost 1,000 TSh would you still take it?

Tick one only:

Ndio *Yes 3*	Labda *Possibly 2*	Hakijulikani *Uncertain 1*	Hapana *No 0*

Narrative:

If "yes" or "possibly", enquire further, otherwise go to Q 4.1:

3.8 "Kama chanjo ya kipindupindu ingegharimu 5,000 TSh ungeitumia?"
If the vaccine were to cost 5,000 TSh would you still take it?

Tick one only:

Ndio *Yes 3*	Labda *Possibly 2*	Hakijulikani *Uncertain 1*	Hapana *No 0*

Narrative:

If "yes" or "possibly", enquire further, otherwise go to Q 4.1:

3.9 "Kama chanjo ya kipindupindu ingegharimu 10,000 TSh ungeitumia?"
If the vaccine were to cost 10,000 TSh would you still take it?

Tick one only:

Ndio *Yes 3*	Labda *Possibly 2*	Hakijulikani *Uncertain 1*	Hapana *No 0*

Narrative:

4 Vignette B

"Sikiliza hadithi ya [mtu huyu]…" *Let me tell you the story about this [person]…*

4.1 "Maradhi haya yanaitwaje?"
What is the name of this disease?

Specify name, summary term or short description in the respondent's own words. If 'other', specify term and explain here:

Narrative:

**Types of diarrhoea**	
1 Kuharisha kawaida *Normal diarrhoea*	*5* Kipindupindu *Cholera*
2 Kuharisha maji *Watery diarrhoea*	*6* Mchanganyiko *Multiple*
3 Kuharisha marenda *Mucous diarrhoea*	*98* Mengineyo, eleza *Other, specify*:
4 Kuharisha damu *Bloody diarrhoea*	*99* Siwezi kusema *Cannot say*

Code the name from the above numbered list: ☐

Use the name as identified for this disease instead of referring to disease/problem and use the name of the person mentioned in the vignette in the following questions.

4.2 "Je unaweza kufikiria dalili nyingine ambazo [mtu huyu] anaweza kuwa nazo mbali na tulizozitaja hapo juu?"
Can you think of any other symptoms that this [person] is likely to experience besides the ones we already mentioned?

Narrative:____

Tick all that apply:

Physical symptoms	Tick		Tick
1 Kusokotwa na tumbo *Abdominal cramps*		*13* Kunyauka, kukauka ngozi *Skin (loose, dry, shrivelled)*	
2 Kuumwa na tumbo *Abdominal pain/discomfort*		*14* Kutokuwa na hamu ya kula *Loss of appetite*	
3 Kuumwa na misuli *Muscle cramps*		*15* Maumiva ya kitchwa *Headache*	
4 Kutapika *Vomiting*		*16* Kichefuchefu *Nausea*	
5 Choo kingi kupita kiasi *Large amounts of stool*		*17* Homa *Fever*	
6 Kuharisha mara kwa mara *Frequent passing of stool*		*18* Udhaifu *Weakness*	
7 Choo kama maji ya mchele *Rice water-like stool*		*19* Kupaparikwa na moyo *Palpitations*	
8 Choo chenye usaha *Pus in stool*		*20* Fadhaisha *Confusion*	
9 Choo chenye damu *Bloody stool*		*21* Kupoteza fahamu *Unconsciousness*	
10 Maumivu sehemu ya kunyea *Rectal pain*		*98* Dalili nyingine mwilini *Other physical symptoms*	
11 Kiu kali *Very thirsty*		*99* Siwezi kusema *Cannot say*	
12 Macho kuingia ndani *Sunken eyes*			

4.3 "Je unafikiri maradhi haya yatamuathiri vipi [mtu huyu] kimawazo, kijamii na kiuchumi katika maisha yake ya kila siku?"
How do you think that this [disease] will affect [this person] emotionally, socially and financially in his/her daily life?

Narrative:____

Tick all that apply:

Impact	Tick		Tick
Social impact		*Emotional impact*	
1 Kutengwa na watu wengine *Isolation from others*		*6* Huzuni, kukosa raha, wasiwasi *Sadness, anxiety, worry*	
2 Woga wa kuambukiza wengine *Fear of infecting others*		*Financial impact*	
3 Kusitisha huduma za afya *Disruption of health services*		*7* Kuongezeka kwa gharama za maisha *Costs (transportation, foods, drugs)*	
4 Kuathiri shudhuli za kila siku *Interference with work/daily activities*		*8* Kupoteza kipato cha familia *Loss of family income*	
5 Kuathiri uhusiano na watu wengine katika jamii *Interference with social relationships*		*Miscellaneous*	
		98 Mengine, eleza *Other, specify:*	
		99 Siwezi kusema *Cannot say*	

4.4 "Unaonaje ubaya wa [maradhi haya] kwa [mtu huyu]?"
How serious is this [disease] for [this person]?

Tick one only:

Mbaya sana *Very serious 3*	Mbaya kiasi *Moderately serious 2*	Haitabiriki *Uncertain 1*	Si mbaya *Not serious 0*

Narrative:____

4.5 "Je afya ya [mtu huyu] itakuwaje ikiwa atakosa matibabu sahihi ya [maradhi haya] kutoka nje ya nyumba yake?"
What is the most likely health outcome of this [disease] for [this person] without appropriate treatment from outside?

Tick one only:

Kifo<br>*Usually fatal 4*	Wakati mwingine inaweza kusababisha kifo<br>*Sometimes fatal 3*	Hali mbaya sana, lakini haisababishi kifo<br>*Serious but not fatal 2*	Haitabiriki<br>*Uncertain 1*	Kupona vizuri na haraka<br>*Full/quick recovery 0*

Narrative:____

4.6 "Kila mmoja wetu anaweza kueleza mambo yanayotokea kwa njia tofauti tofauti. Je unafikiria nini kimesababisha tatizo la [mtu huyu]?"
Each of us may explain something that happens in various ways. What do you think has caused this [person]'s problem?

Narrative:____

Tick all that apply:

Perceived causes	Tick		Tick
Ingestion		*8* Nzi *Flies*	
1 Kunywa maji machafu *Drinking contaminated water*		*9* Malaria	
2 Chakula ambacho hakijahifadhiwa/kimeoza *Unprotected/spoiled food (biological)*		*10* Minyoo *Worms*	
3 Chakula kilichokatazwa *Forbidden food (taboo)*		*Magico-religious causes*	
4 Kula udongo *Eating Soil*		*11* Uchawi *Witchcraft*	
Behaviour		*12* Rehema ya *Mungu God's will*	
5 Kugusa maji machafu *Contact with contaminated water*		*Miscellaneous*	
6 Kutoosha mikono *Not washing hands*		*98* Mengine, eleza *Other, specify:*	
Environment		*99* Siwezi kusema *Cannot say*	
7 Mazingira machafu *Dirty environment*			

4.7 "Je, watu humsaidia nini nyumbani [mtu] mwenye [maradhi haya] kabla ya kutafuta matibabu ya nje ya nyunbani kwao?"
What do people do at home for a [person] with this [disease] before looking for treatment or help outside their homes?

Narrative:____

Tick all that apply:

Home-based treatment	Tick
1 Kunywa maji mengi au vinywaji vingine *Drinking more water or liquids*	
2 Dawa za mitishamba (mizizi, magamba, majani) *Herbal treatment (roots, bark, leaves)*	
3 Kunywa dawa za vipaketi (ORS) zinazouzwa madukani *Oral rehydration therapy*	
4 Kuomba dua *Prayers*	
5 Kutumia dawa za antibiotics za kujinunulia mwenyewe *Self-administered antibiotics/drugs*	
6 Hapana *Nothing*	
98 Mengine, eleza *Other, specify:*	
99 Siwezi kusema *Cannot say*	

4.8 "[Mtu] mwenye [maradhi haya] kwa kawaida ataenda wapi kupata matibabu nje ya nyumba yake?"
Where will [this person] usually go for treatment outside his/her home?

Narrative:____

Tick all that apply:

Outside treatment	Tick
1 Hospitali *Health facilities*	
2 Waganga wa kienyeji *Traditional healers*	
3 Maduka ya madawa *Pharmacy or over-the-counter drugs*	
4 Viongozi vya dini *Faith healers (Imams, Sheikh)*	
5 Ushauri kutoka kwa ndugu/jamaa na marafiki wanaofanya kazi vituo vya afya *Informal help from health-worker, friend/relative*	
98 Sehemu nyinginezo, eleza *Other, specify:*	
99 Siwezi kueleza *Cannot say*	

Concluding advice from respondent

"Je, kutokana na ujuzi na uzoefu wako unaweza kuniambia zaidi kuhusu matatizo ya afya ambayo tumeyajadili au kuhusu chanjo? Maoni, ushauri na mapendekezo yanakaribishwa."
Is there anything else you can tell me about the health problems we have discussed or about vaccinations from your experience? Any further comments, advice or suggestions will be appreciated.

Narrative:____

Team (*circle appropriate*)	Unguja: A B C; Pemba: D E F
Interviewer (name/signature)	
Recorder (name/signature)	
Controlled (date/initials)	
Narratives translated & typed (date)	
1st Data entry (date/initials)	
2nd Data entry (date/initials)	

Additional comments from the interview team

Notes concerning the participant's interest and the quality of the interview, and other noteworthy features and details of this interview:
