# Supplementary material for: Comparing sociocultural features of cholera in three endemic African settings
Source: BMC Med. 2013 Sep 18;11:206. doi: 10.1186/1741-7015-11-206 (PMC4016292; doi:10.1186/1741-7015-11-206)
Supplement: Additional file 4 — Comparison of priority symptoms for cholera in endemic areas of three African settings, by site and gender. [file 1741-7015-11-206-S4.pdf]

Additional file 4 Comparison of priority symptoms for cholera in endemic areas of three African settings, by site and gender

a. Overall	SE-DRC, n=360		Western Kenya, n=379		Zanzibar, n=356	
	Total rep.	Prom.	Total rep.	Prom.	Total rep.	Prom.
Loss of appetite	86.7	1.24	**91.3**	**1.10**	87.9	1.04
Sunken eyes[a] ***	**97.5**	**1.44**	**93.9**	**1.00**	**94.9**	**1.26**
Unconsciousness***	**90.3**	**1.44**	74.4	0.94	**91.6**	**1.62**
Weakness[b] ***	**98.3**	**2.61**	**94.7**	**1.55**	**97.2**	**1.88**
Cannot say***	4.7	0.12	37.5	1.26	18.5	0.40

b. Site comparison	SE-DRC Urban		SE-DRC Rural			Western Kenya Urban		Western Kenya Rural			Zanzibar Urban		Zanzibar Rural		
	Total rep.	Prom.	Total rep.	Prom.	p value	Total rep.	Prom.	Total rep.	Prom.	p value	Total rep.	Prom.	Total rep.	Prom.	p value
Blood in stool	18.3	0.23	18.3	0.20	0.853	61.1	0.70	55.0	0.77	0.535	**23.5**	**0.28**	**50.3**	**0.60**	**<0.001**
Loose skin	**97.2**	**1.33**	**96.7**	**1.43**	**0.035**	85.8	0.93	83.6	0.93	0.640	90.5	1.09	88.1	1.11	0.737
Loss of appetite	82.2	1.25	91.1	1.23	0.994	**94.2**	**1.14**	**88.4**	**1.06**	**0.042**	**92.2**	**1.16**	**83.6**	**0.92**	**<0.001**
Mucus in stool	39.4	0.47	38.3	0.43	0.716	62.1	0.64	59.8	0.67	0.844	**8.9**	**0.09**	**37.9**	**0.38**	**<0.001**
Nausea	**88.9**	**1.14**	**91.7**	**1.00**	**0.043**	86.8	0.91	84.1	0.92	0.939	87.7	0.93	88.1	0.93	0.991
Palpitations	75.6	0.93	91.1	0.99	0.235	67.9	0.70	62.4	0.77	0.570	**84.4**	**1.16**	**73.4**	**0.80**	**<0.001**
Rectal pain	**63.9**	**0.72**	**52.8**	**0.56**	**0.012**	75.8	0.77	67.7	0.70	0.090	69.3	0.69	73.4	0.79	0.270
Sunken eyes	**96.1**	**1.30**	**98.9**	**1.57**	**<0.001**	95.3	1.03	92.6	0.98	0.105	**93.9**	**1.15**	**96.0**	**1.37**	**<0.001**
Unconsciousness	87.2	1.47	93.3	1.41	0.733	75.3	0.84	73.5	1.05	0.210	**92.7**	**1.94**	**90.4**	**1.30**	**<0.001**
Weakness[b]	**96.7**	**2.37**	**100.0**	**2.86**	**0.003**	98.9	1.61	90.5	1.49	0.257	96.6	1.92	97.7	1.84	0.209
Cannot say	3.9	0.12	5.6	0.13	0.816	**34.7**	**1.09**	**40.2**	**1.42**	**0.036**	15.1	0.32	22.0	0.47	0.090

c. Gender comparison	SE-DRC Female		SE-DRC Male			Western Kenya Female		Western Kenya Male			Zanzibar Female		Zanzibar Male		
	Total rep.	Prom.	Total rep.	Prom.	p value	Total rep.	Prom.	Total rep.	Prom.	p value	Total rep.	Prom.	Total rep.	Prom.	p value
Fever	84.5	0.88	82.7	0.89	0.887	**64.9**	**0.70**	**74.6**	**0.83**	**0.044**	85.5	0.91	84.2	0.98	0.679
Palpitations	84.5	0.96	82.1	0.96	0.956	**70.1**	**0.84**	**60.0**	**0.62**	**0.016**	79.3	1.01	78.5	0.95	0.562
Very thirsty	89.0	1.12	88.3	1.02	0.249	82.0	0.87	86.5	0.93	0.275	**83.2**	**0.93**	**71.8**	**0.79**	**0.021**

Categories ordered alphabetically, except for "cannot say." 'Total rep.'='Total reported,' referring to percentage of categories reported spontaneously and upon probing. 'Prom.'='Prominence,' referring to mean prominence of categories based on how reported (spontaneous=2, probed=1, most troubling=3). [a] This category considered among the top three in Western Kenya although prominence for "cannot say" was higher. [b] One response missing in Western Kenya (n=378). Panel a: Figures in bold designate top three prominent categories; comparison between settings based on Kruskal Wallis test, *** $p<0.001$. Panels b and c: Figures in bold designate significant differences at $p<0.05$ based on Wilcoxon test. SE-DRC: Southeastern Democratic Republic of Congo.

Data for Zanzibar in panel b from: Schaetti C, Khatib AM, Ali SM, Hutubessy R, Chaignat CL, Weiss MG: **Social and cultural features of cholera and shigellosis in peri-urban and rural communities of Zanzibar**. *BMC Infect Dis* 2010, **10**:339. Data for Western Kenya in panel a and b from: Nyambedha EO, Sundaram N, Schaetti C, Akeyo L, Chaignat CL, Hutubessy R, Weiss MG: **Distinguishing social and cultural features of cholera in urban and rural areas of Western Kenya: Implications for public health**. *Glob Public Health* 2013, **8**:534-551.
